# Modeling of Anticancer Sulfonamide Derivatives Lipophilicity by Chemometric and Quantitative Structure-Retention Relationships Approaches

**DOI:** 10.3390/molecules27133965

**Published:** 2022-06-21

**Authors:** Monika Pastewska, Beata Żołnowska, Strahinja Kovačević, Hanna Kapica, Maciej Gromelski, Filip Stoliński, Jarosław Sławiński, Wiesław Sawicki, Krzesimir Ciura

**Affiliations:** 1 Department of Physical Chemistry, Medical University of Gdańsk, Al. Gen. Hallera 107, 80-416 Gdańsk, Poland; monika-pastewska@gumed.edu.pl (M.P.); hanna.kapica@gumed.edu.pl (H.K.); wsawicki@gumed.edu.pl (W.S.); 2 Department of Organic Chemistry, Medical University of Gdańsk, Al. Gen. Hallera 107, 80-416 Gdańsk, Poland; beata.zolnowska@gumed.edu.pl (B.Ż.); jaroslaw.slawinski@gumed.edu.pl (J.S.); 3 Department of Applied and Engineering Chemistry, Faculty of Technology Novi Sad, University of Novi Sad, Bulevar Cara Lazara 1, 21000 Novi Sad, Serbia; strahinjakovacevic@hotmail.com; 4 QSAR Lab Ltd., Trzy Lipy 3 St., 80-172 Gdańsk, Poland; m.gromelski@qsarlab.com (M.G.); f.stolinski@qsarlab.com (F.S.); 5 Laboratory of Environmental Chemoinformatics, Faculty of Chemistry, University of Gdansk, Wita Stwosza 63, 80-308 Gdansk, Poland

**Keywords:** sulfonamide derivatives, quantitative structure-retention relationships, lipophilicity

## Abstract

Sulfonamides are a classic group of chemotherapeutic drugs with a broad spectrum of pharmacological action, including anticancer activity. In this work, reversed-phase high-performance liquid chromatography and biomimetic chromatography were applied to characterize the lipophilicity of sulfonamide derivatives with proven anticancer activities against human colon cancer. Chromatographically determined lipophilicity parameters were compared with obtained log*P*, employing various computational approaches. Similarities and dissimilarities between experimental and computational log*P* were studied using principal component analysis, cluster analysis, and the sum of ranking differences. Furthermore, quantitative structure–retention relationship modeling was applied to understand the influences of sulfonamide’s molecular properties on lipophilicity and affinity to phospholipids.

## 1. Introduction

Due to inadequate pharmacokinetic properties, many drug candidates are rejected during clinical trials. Under those circumstances, besides biological activity, the physicochemical properties of putative drug molecules must be optimized at the early stage of drug development. These actions are carried out to achieve the desired in vivo drug metabolism and pharmacokinetics (DMPK) profile [1]. Among available screening methods, chromatography has a well-established position [2,3], especially in lipophilicity profiling.

The lipophilicity is a crucial physicochemical parameter of every molecule, affecting toxicity, absorption, distribution, metabolism, and drug elimination [4]. Retention in reversed-phase high-performance liquid chromatography (RP-HPLC) is governed by lipophilicity; consequently, retention data can be considered a surrogate of log*P*. The International Union of Pure and Applied Chemistry (IUPAC) and the Organization for Economic Co-operation and Development (OECD) consider RP-HPLC an equivalent method to the traditional shake-flask approach for log*P* estimation. Generally, the chromatographic technique is low-cost, fast, and requires a small amount of target substances that do not need to be pure, as their impurities are readily separated during the chromatographic process. Furthermore, the chromatographic analyses are repeatable and robust. For these reasons, the solid–liquid partitioning methods are very convenient in the early steps of the drug discovery pipeline, when high throughput is more required than accuracy.

Additionally, the current chromatographic approach can be more similar to the biological partition process since available stationary phases are covered on even more similar or similar components of in vivo distribution media. Such an approach is called biomimetic chromatography. Generally, biomimetic chromatography combines the advantages of high throughput analysis, which are being user-friendly, rapid, reproducible, and compound sparing with the ability to reflect physiological conditions [5,6,7]. An immobilized artificial membrane (IAM) and protein-covered stationary phases bed can be distinguished among biomimetic stationary phases. IAM chromatography has been introduced to simulate membrane barrier passage of complex biological barriers, including gut endothelium, skin, and the blood–brain barrier [5,8,9,10]. 

Sulfonamides are a classic group of chemotherapeutic drugs with a broad spectrum of pharmacological action [11]. Historically, this class of compounds derives from the simple sulfanilamide, which was the leading structure for the development of essential drugs such as antibacterial sulfathiazole [11], antiglaucoma acetazolamide [12], diuretic furosemide [13], hypoglycemic agent glibenclamide [14], and antiviral amprenavir [15]. For years, scientific reports have indicated that sulfonamide derivatives show in vitro and in vivo antitumor activity with various mechanisms of action, including carbonic anhydrase inhibition, cell cycle perturbation in the G1 phase, and inhibition of tubulin polymerization, or angiogenesis inhibition (inhibition of extracellular matrix metalloproteinases) [16].

Among the anticancer sulfonamides, the arylsulfonamides with clinical importance in the treatment of severe diseases are pazopanib (malignant neoplasms of the kidneys and soft tissue sarcoma) [17,18], belinostat (lymphoma from peripheral T cells) [19], and dabrafenib (unresectable and malignant melanoma) [20]. These compounds have different mechanisms of action and act as tyrosine kinase inhibitors, histone deacetylase inhibitors, and BRAF inhibitors. At least four *N*-acylsulfonamides have been clinically investigated as drug candidates with cytostatic activity against tumors: LY573636-sodium salt (Tasisulam) [21,22], ABT-737 [23], ABT-263 (Navitoclax) [24,25] and ABT-199 [26]. The last three drugs are inhibitors of antiapoptotic Bcl-2 family proteins and have found application in treating leukemia and lung cancer [23,24,25,26].

Broad-spectrum antitumor activity depends on the substituents at the benzene ring of arylsulfonamide and the moieties attached to the nitrogen atom of the sulfonamide group. Recently, pro-apoptotic activity of *N*-(1,2,4-triazin-3-yl)benzenesulfonamides, *N*-(5-oxo-1,2,4-triazin-3-yl)benzenesulfonamides and *N*-(heteroaryl)-4-(1H-pyrrol-1-yl)benzenesulfonamides has been proven for human colon cancer (HCT-116) cell [27,28,29]. Apoptosis was confirmed by changes in cell morphology, DNA fragmentation, loss of mitochondrial membrane potential, phosphatidylserine translocation into the outer leaflet of the cell membrane, and activation of caspases [29]. The anticancer potential against the HCT-116 cell line has also been demonstrated by *N*-acylated benzenesulfonamides, which contained at the benzene ring the 1-naphthylmethylthio substituent and the sulfonamidic nitrogen atom bonded to acetic acid or cinnamic acid residues [30]. Similarly, fused pyrimidine hybrids of myrrhanone C and Argentatins A–C showed noticeable anticancer activity against several human cancer cell lines [31,32]. The main goal of this study was to assess the sulfonamide derivatives with proven anticancer activities against human colon cancer (HCT-116) using a chromatographic approach. Retention of sulfonamide derivatives was examined on four RP-HPLC stationary phases, various in terms of chemical modification and IAM-HPLC. Using a chemometric method, experimentally determined chromatographic lipophilicity indices have been compared with calculated log*P*. Furthermore, Quantitative Structure–Retention Relationship (QSRR) models, which allow for predicting the retention or lipophilicity of these chemical compounds, have been proposed. Finally, we aimed to analyze which type of lipophilicity indices, computational or chromatographic, provided a better prediction of anticancer activity expressed as pIC_50_. The obtained models were used using the genetic algorithm partial least squares (GA-PLS) approach.

## 2. Results and Discussion

It is well established that the computational approaches for lipophilicity estimation have several advantages over the experimental methods, including short calculation time and saving chemical reagents. Furthermore, it allows for lipophilicity prediction before synthesis; therefore, it can be applied to the design of potential drug candidates. These advantages make computational approaches desirable from economic and environmental points of view. Nevertheless, it should be emphasized that significant differences between the calculated log*P* values for the same molecules using various theoretical approaches are the computational methods’ crucial limitations [33,34,35,36]. 

Similarly, this is also evident in the calculated log*P* values for the investigated sulfonamides derivatives. As can be observed in Table 1, the calculated log*P* values are very diversified within a single substance. On average, within one molecule, the calculated log*P* values vary by four Log*P* units. It is most noticeable for molecule no. 21, where the difference between WLog*P* and iLog*P* states 7.3 clog*P* units. Indicated differences can be explained by the varied nature of the algorithms employed in the applied software programs [37]. The lowest calculated log*P* values are achieved by the iLog*P* descriptor (in 16 cases), and the MLog*P* descriptor follows it in 10 cases. The iLog*P* descriptor is a physics-based algorithm established on free solvation energies, whereas the MLog*P* descriptor investigated the hydride algorithm of topological and molecular properties. Each compound has a log*P* with a value less than five for both mentioned algorithms and reached the Lipinski rule of five [38]. On the other hand, in most cases, higher calculated log*P* is observed for descriptor WLog*P*, based on purely atomistic methods.

Among tested derivatives, the most hydrophilic substance, according to each algorithm, is compound no. **1**. Nevertheless, selecting the most lipophilic substance based on calculated log*P* is difficult. According to the descriptors WLog*P* (atomistic method), Silicos-IT Log*P* (hybrid fragmental/topological method), and Consensus Log*P* (average of all five predictions), the most lipophilic compound is no. **21**. However, compounds **19, 27**, and **11** are the most lipophilic accordingly to the descriptors iLog*P*, XLog*P* (atomistic and knowledge-based method), and KOWWIN Log*P* (atom-based approach and fragmental contribution). Additionally, compound no. **27**, in the case of the Xlog*P* descriptor, is also the most lipophilic substance according to the Mlog*P* descriptor.

The obtained contradiction suggested that it is still worth using experimental methods to assess drug candidates’ lipophilicity. For this reason, the next step of our study concerns the characterization of investigated sulfonamide derivatives using a chromatographic approach. Generally, in RP-HPLC, the interactions between solutes and the stationary phase surface are mainly governed by lipophilicity [36,39,40]. Consequently, RP-HPLC is the primary tool for assessing the lipophilicity of xenobiotics [41]. Nevertheless, the one unique protocol for chromatographic lipophilicity measurement, which can be applied to analyze each chemical group, does not exist. Generally, the chemical character of target solutes determines interactions between the molecule and the stationary phase that may occur. Considering the large variety of RP-HPLC beds and differences in specific surface area, the energy of the sorbent’s intermolecular interactions between solutes and chemical structures, four different reversed-phase stationary phases and IAM column were investigated during this study. The lipophilicity index was presented as a log*k*_w_ value*,* so the retention factor was extrapolated to the pure water after calculation using a protocol based on two gradient measurements and DryLab software [42,43]. In the case of IAM and C_18_ chromatography, we applied protocols proposed by Valko and co-workers [44,45] and subsequently linearly transformed data into log*k*_IAM_ [46] and CHI log D [47] using the following formulas:

log*k*_IAM_ = 0.045 CHI_IAM_ + 0.42 (1)

CHI log D = 0.0525 CHI_C18_ − 1.467 (2)

Since the biomimetic chromatography experiments are carried out at physiological pH to compare obtained results, all chromatographic experiments have been carried out at pH 7.4. Calculated pKa indicates that sulfonamide derivatives molecules are in a charged state. Consequently, the chromatography lipophilicity indices refer to the distribution coefficient (logD) values, which better reflect the lipophilicity of ionized substances under physiological conditions. The chromatographically obtained lipophilicity indices of sulfonamide derivatives are presented in Table 2.

**Figure 1:**
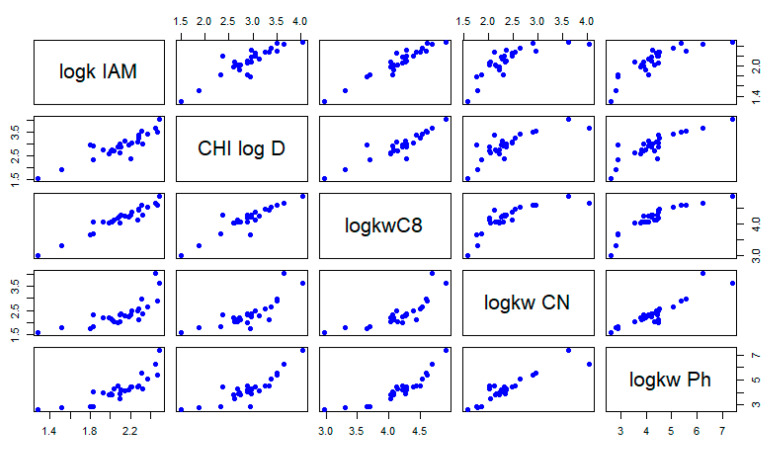
The correlation plot of experimentally determined lipophilicity indexes.

As might be expected, the obtained chromatographic indexes are generally highly correlated (Figure 1). A high correlation was noticed between IAM, C_18_, and C_8_ columns (r < 0.91). On the contrary, significantly lower correlation coefficients were achieved for the Ph and CN columns and the above-mentioned stationary phase (0.76 < r < 0.89), indicating a significant difference in the sulfonamide retention mechanism using these beds.

Based on the experimental data, compound no. **1** turned out to be the least lipophilic substance. The calculation methods provided the same conclusion as experimental methods in selecting the least lipophilic drug candidate from tested sulfonamide derivatives. Contrarily, the most lipophilic properties are in compound no. **4**, taking only the chromatographic experiments. However, none of the calculation algorithms selected this molecule as a highly lipophilic substance. Surprisingly, according to the calculation methods, it is one of the least lipophilic derivatives. These data indicate that experimental methods are still necessary for the drug development pipeline. 

Considering the proportion of lipophilicity and anticancer activity, the assumption that the compound should be characterized by the lowest lipophilicity while maintaining pharmacological action for further tested molecules, **1**–**4** and **26**, can be recommended. Assessing the overall lipophilicity of the tested compounds, it can be said that it is at a reasonably high level. Compounds containing the 1-naphthyl substituent are especially more lipophilic. To obtain a lower, more desirable lipophilicity, the phenyl substituent should be more desirable because the analyzed compounds which comprise it, as a rule, had significantly lower lipophilicity.

Next, we aimed to analyze similarities and dissimilarities between chromatographic and computational lipophilicity indexes of the target molecules; therefore, the principal component analysis (PCA), cluster analysis (CA), and the sum of ranking differences (SRD) were performed. 

**Figure 2:**
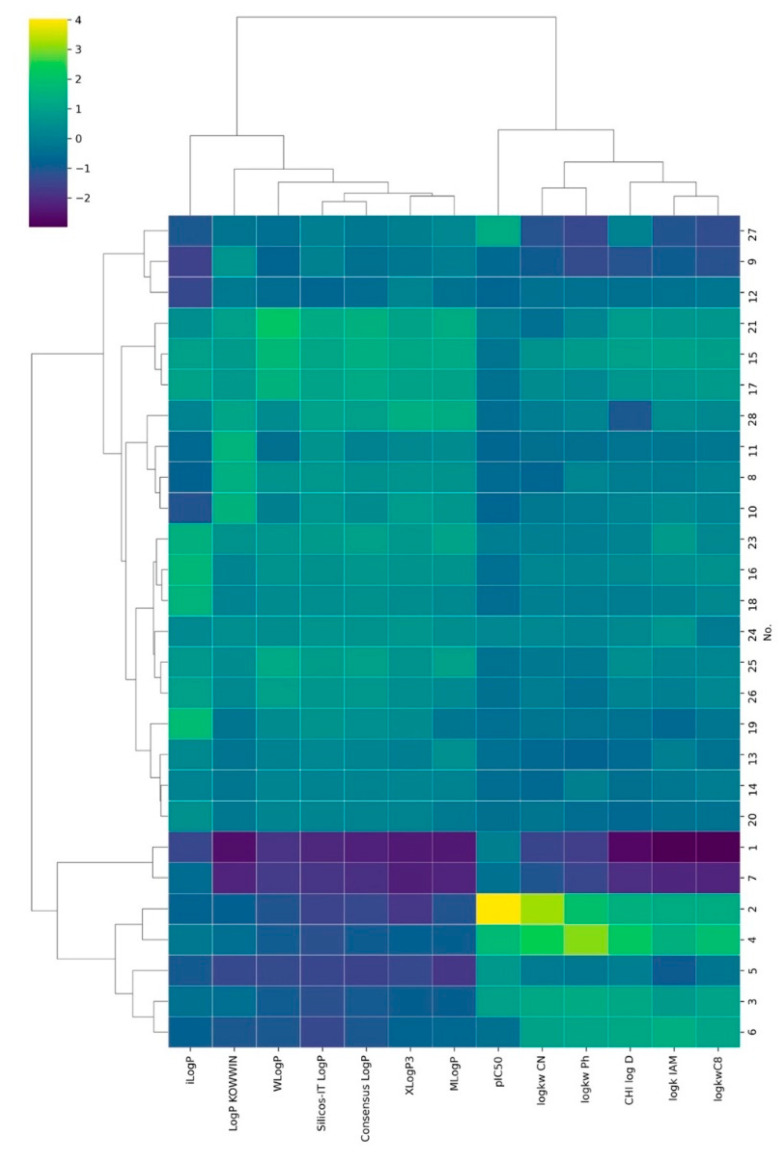
Results according to Ward’s agglomeration rule and the Euclidian distance measure results of CA analysis.

The CA analysis results are presented in Figure 2 as a heatmap. Two main groups of lipophilicity indexes, the computational and the chromatographical, are very clearly marked. One of the obvious explanations for the grouping results obtained may be that the calculation methods did not show the ionization of the compounds that took place during the chromatographic experiments. Additionally, the heatmap of HCA provided information about grouping in the space of the compounds under study. Here, we can easily separate two groups; one included only pirolo derivatives (molecules no **1**–**7**) and the second one where other derivatives are grouped. Our strategy also allows us to compare the similarity of anticancer activity expressed as pC_50_ concerning computational and experimental methods, determining lipophilicity. Interestingly, pIC50 is a group close to chromatographic that determines lipophilicity. Therefore, we cautiously conclude that chromatographic indices can be more effective for predicting anticancer activity than computational lipophilicity indices.

**Figure 3:**
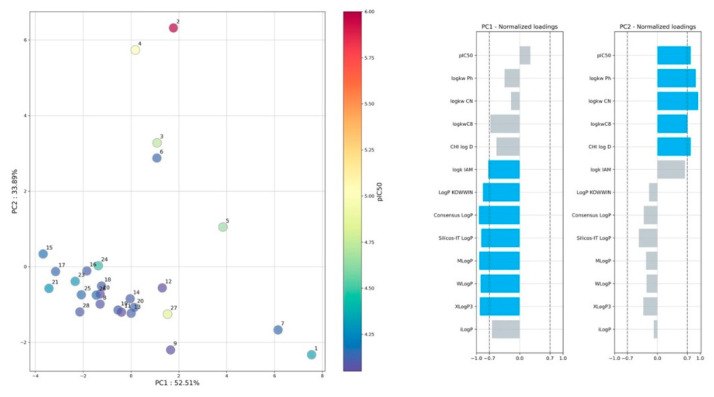
Results of PCA analysis, projection on PC_1_ and PC_2_ space.

Similar results are observed on the PCA (Figure 3), where the first two PCs distinguish the parameters of computational and experimental lipophilicity. A normalized loadings plot (Figure 3) indicated that PC1 affected computational indices, whereas on PC_2_ it mainly affected the mainly chromatographic parameters, except IAM chromatography. Interestingly, the IAM stationary phase has a significant impact on both PCs. The surface of IAM is mostly zwitterionic at pH 7.4, and positively charged choline moieties are located in the outer part of the IAM layer. In contrast, the phosphate groups are negatively charged at the same pH and present in the phase’s inner part. This distinguishes the IAM phase from the rest tested RP-HPLC beds.

The next step of our investigation concerned the application of SRD analysis to select the best and the worst approaches for lipophilicity measurement. The limitations of unsupervised chemometric tools such as PCA and CA are that these methods do not provide any information about statistical figures of performed analysis. Consequently, the SRD analysis was used to complete the data analysis and support the selection of the best lipophilicity indices. 

**Figure 4:**
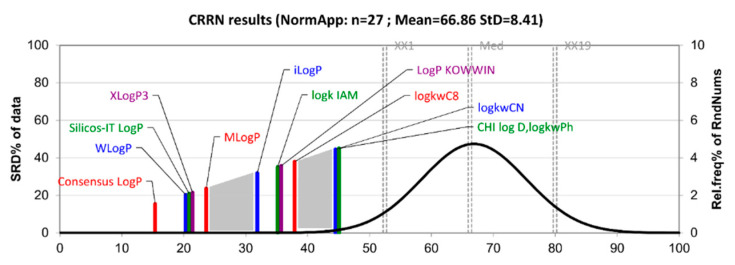
The lipophilicity parameters of sulfonamide derivatives are ranked by the sum of ranking differences and comparison of ranks by random numbers with row average as a reference ranking. The SRD values are normalized between 0 and 100 compared to the random ranking depicted by the black cumulative distribution function. The statistical characteristics of Gaussian fit are the following: first icosaile (5%), XX1 = 192; first quartile, Q1 = 220; median, Mediana (Med) = 242; last quartile, Q3 = 262; last icosaile (95%), XX19 = 292.

The results of the SRD analysis are presented in Figure 4. The scaled SRD values are plotted on the *x*-axis and left *y*-axis, while the right *y*-axis gives the cumulated relative frequencies for random ranking (black curve). The presented graph indicates the following facts: the Consensus Log*P* descriptor is placed closest to the reference ranking and can be considered the right choice for lipophilicity estimation of the studied series of compounds; the parameters log*k*_wCN_, CHI logD_C18_ and log*k*_wPh_ are places furthest from the reference ranking and therefore are depicted as the least suitable lipophilicity descriptors of sulfonamide derivatives; specific groupings of the parameters can be observed; Wlog*P*, Silicos-IT Log*P* and XLog*P*3 have very close SRD values and the same stands for log*k*_IAM_ and Log*P* KOWWIN descriptors as well as for log*k*_wCN_, CHI logD_C18_ and log*k*_wPh_ parameters; looking globally, there are two clearly visible gaps (gray zones in Figure 4) dividing the lipophilicity parameters into three main groups regarding the SRD values; the group consisting of in silico descriptors including Consensus Log*P*, WLog*P*, Silicos-IT Log*P*, XLog*P*3 and MLog*P* is closest to the reference ranking, while all the experimentally determined lipophilicity measures are placed far from the reference ranking.

**Figure 5:**
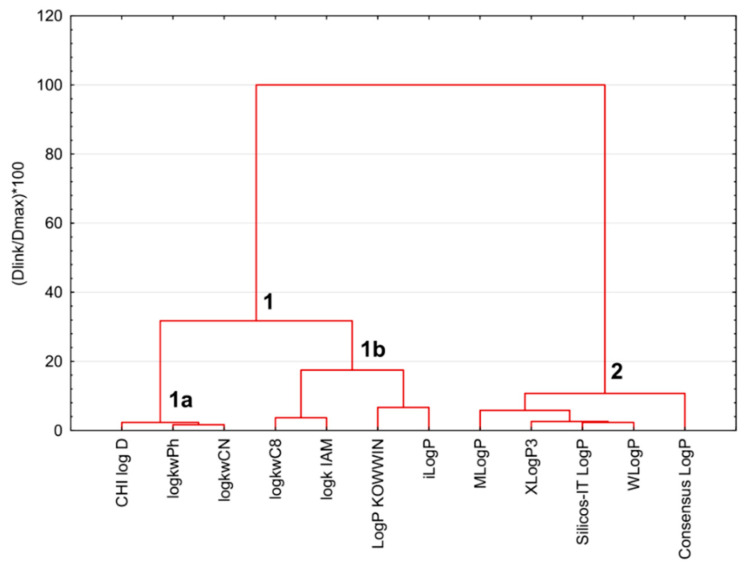
The lipophilicity parameters of sulfonamide derivatives were clustered in the space of the SRD% values by 7-fold cross-validation.

**Figure 6:**
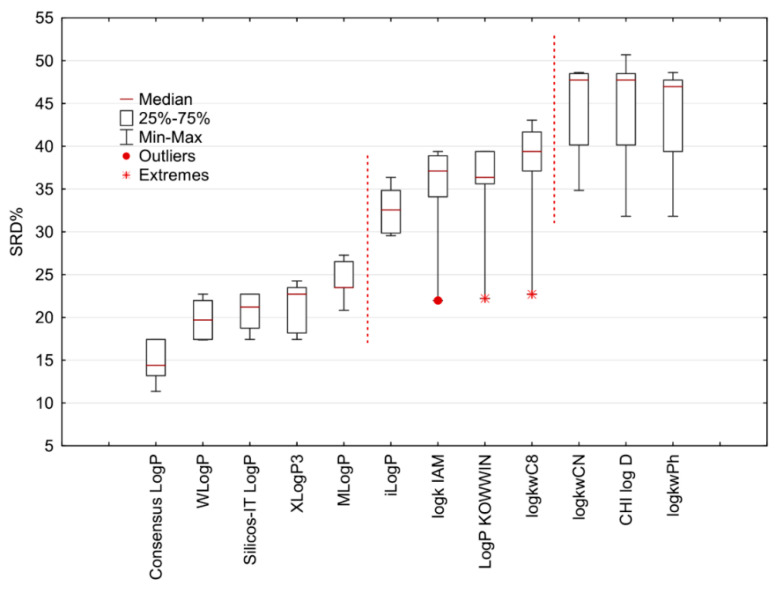
The box and whisker plot shows the normalized SRD values (SRD%) estimated by 7-fold cross-validation procedure.

**Figure 7:**
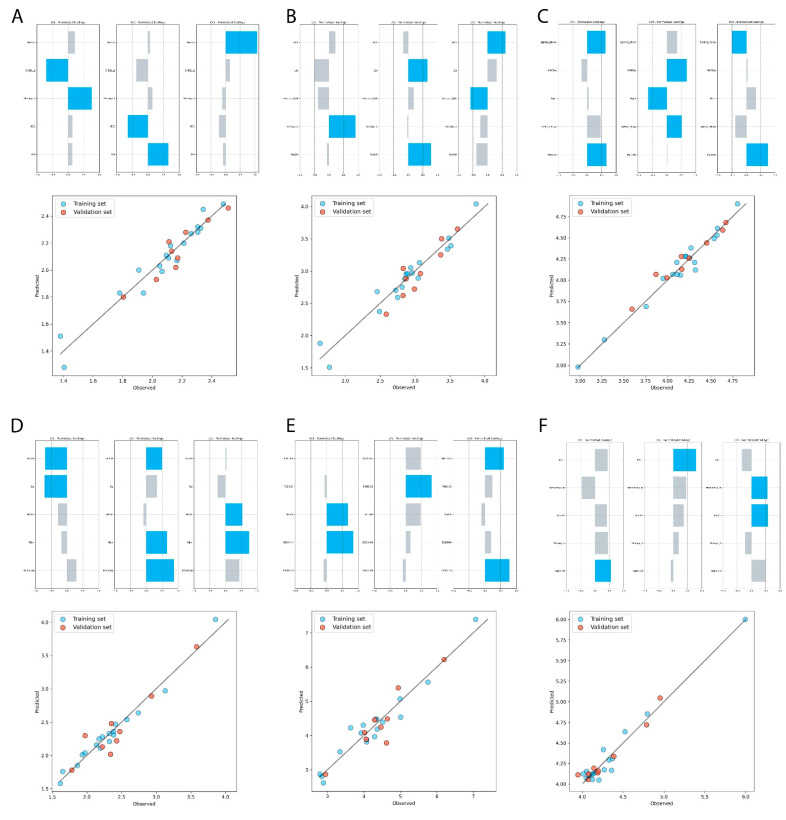
Vertical bar plot for normalized loadings and comparison between the experimental chromatographic indices predicted by GA-PLS QSRR and QSAR models. The numbers correspond to the subsequent stationary phases (**A**) IAM, (**B**) C_18_, (**C**) C_8_, (**D**) CN, (**E**) Ph and (**F**) pIC_50_.

The SRD procedure was validated by 7-fold cross-validation. The results are presented in two ways in Figure 5 and Figure 6. The clustering of the experimental and in silico lipophilicity descriptors of sulfonamide derivatives in the space of the normalized SRD values (SRD%) obtained by 7-fold cross-validation is presented in the form of a dendrogram in Figure 7. Two main clusters are observable: cluster #1 contains two sub-clusters, 1a sub-cluster (CHI logD_C18_, log*k*_wPh_ and log*k*_wCN_) and 1b (log*k*_wC8_, log*k*_IAM_, Log*P* KOWWIN, iLog*P*); cluster #2 contains MLog*P*, XLog*P*3, Silicos-IT Log*P*, WLog*P* and Consensus Log*P* parameters. The groupings of the parameters suggested in the SRD graph and the dendrogram comply with each other.

Additionally, the results of the 7-fold cross-validation of the SRD procedure are presented in Figure 6 in the box and whisker plot; the parameters are arranged in the same order as in the SRD graph. The Consensus Log*P* parameter has the lowest median of the SRD data and is definitely depicted as the best lipophilicity parameter among the other calculated and experimentally determined parameters. There is a significant difference between the lipophilicity parameters separated by vertical dotted lines at the 5% level according to the Wilcoxon matched-pair test, which agrees with the separation of the parameters suggested in the SRD graph (Figure 5) and on the dendrogram (Figure 6). The highest median can be observed for CHI logD_C18_, log*k_w_*_Ph,_ and log*k_w_*_CN_ parameters as the parameters with the highest SRD values. To conclude, the chromatographically determined lipophilicity measures do not outperform the computationally estimated lipophilicity parameters. Nevertheless, among the chromatographic lipophilicity parameters of the analyzed sulfonamide derivatives, log*k*_IAM_ can be considered the best choice.

The next step of our study focuses on QSRR modeling. This methodology, proposed by Kaliszan, presents the relationship between retention and analyte structures [48]. On the one hand, obtained QSRR models allow insights into the molecular mechanism of retention. Therefore, they help us understand what molecular properties govern the chromatographically determined lipophilicity. On the other hand, the established QSRR model supports lipophilicity prediction in similar structures. The selection of theoretical descriptors which influence the retention factors employed GA-PLS. Briefly, GA is a stochastic approach that helps solve the variable selection problem. Therefore, the integration of GA with PLS may be helpful for the development of highly predictive and precise QSPR models. We chose the partial least squares (PLS) as regression mode since it can be used to analyze highly correlated data, which is frequently observed in the case of molecular descriptors.

Finally, five models of GA-PLS QSRR, each describing retention in studied chromatographic systems, were calculated. Statistical figures of obtained models are summarized in Table 3, whereas in Figure 7 the contribution of the descriptors to the individual LVs is presented. The values of theoretical descriptors and their description are listed in Tables S7 and S8, respectively. 

The interpretation of established models can be challenging since many applied descriptors are based on a complex matrix and weighted by different functions. Nevertheless, such holistic descriptors guarantee the coverage of the molecular structure space more efficiently than if limited to only a few mechanical descriptors. Holistic descriptors consider not only the presence of some chemical groups and pharmacophore fragments but also the relative position [49]. Several descriptors of well-recognized molecular properties, which govern retention in reserved phase chromatography, can be found. Great examples are descriptors weighted by polarizability, such as R5p+, R6p+, and SpMin2_Bh(p), or coded lipophilic pharmacophore (SHED_LL). 

Next, we aimed to analyze which type of lipophilicity indices, computational or chromatographic, provided a better prediction of anticancer activity expressed as pIC_50_. The obtained PLS model confirmed findings, which can be concluded after analysis of CA since log*k*_CN_ is one of the most important descriptors. This finding suggested that experimentally determined lipophilicity exceeds that obtained computationally in the screening of biological activity of the tested sulfonamides. As expected, the obtained models also included other types of molecular descriptors. Generally, lipophilicity gives information related to the drug membrane permeability. The steric and electrostatic properties are fundamental to the interaction between molecules and receptors. This statement is also supported by the proposed model where descriptors related to molecule charge (RPCG), polarizability (G2p), and geometrical properties (WHALES00_IR) significantly affect the anticancer activity of this class of chemicals. Nevertheless, comprehensive research is needed on a larger group of compounds to generalize this statement.

The statistical figures of the training and testing set indicated that obtained models are well fitted (*R^2^*,*Q^2,^* and RMSE_CV_) and show suitable predictive parameters (RMSE_P_). Additionally, the applicability domain assessment (AD) and the y-randomization tests were performed for each model.

The applicability domain of predictive models was assessed using a leverage approach (Figure S1 in Supplementary Materials), where the leverages (*x*-axis) are plotted against standardized residuals (*y*-axis) on a so-called William’s plot. The leverages are calculated from the descriptor matrix and then compared to a critical h* value, represented by a vertical dashed line. Additionally, values of 3 (±σ) standardized residual units (horizontal dashed lines) define the cut-off value for acceptable predictions. The study revealed that in three models (B, C, D in Figure S1), one compound from each validation set (6, 26, and 4, respectively) had a higher leverage value than the critical value (h*), indicating that their structure differs significantly. However, these compounds did not exceed the cut-off value for acceptable predictions (±3σ), resulting in very low residuals. Therefore, the developed models will provide correct predictions, even when extrapolated, for compounds that differ significantly in structure.

Each developed model was additionally subjected to a y-randomization test to confirm their robustness and that the linear relationships are not derived by chance (Figure S1 in Supplementary Materials). The performance of the original model is tested by permuting the response variable (y) and then building the model on the primary dataset of descriptors (X). We performed 200 permutations (random models) in the presented study for each developed model. All tests confirmed that in every case, the original model was robust and not derived by chance (the randomly generated models had significantly lower R^2^ and Q^2^ values).

## 3. Materials and Methods

### 3.1. Chemical Reagents

#### 3.1.1. Sulfonamides Derivatives

**Figure 8:**
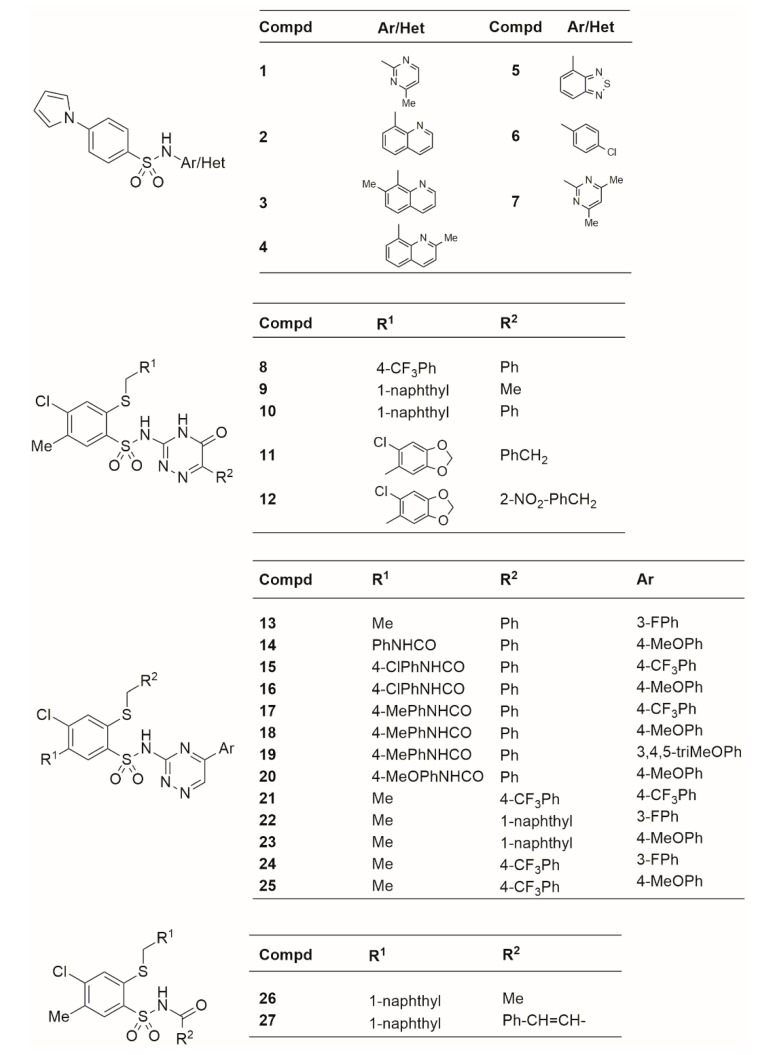
2D structures of investigated sulfonamide derivatives.

The chemical names and SMILES notation of the target sulfonamide derivatives are presented in Table S1, whereas the 2D structures are shown in Figure 8. Their synthesis and characterization were described in the literature [27,28,29,30]. The 1H NMR and 13 C NMR spectra of target molecules are displayed in the Supplementary Materials. All samples were dissolved in DMSO (1 mg/mL). Each stock solution of analytes was stored at 2–8 °C between analyses. During this study, four chemical classes of sulfonamide derivatives were tested, including 1*H*-pyrrole derivatives, 5-oxo-1,2,4-triazine derivatives, 1,2,4-triazine derivatives, and *N*-acylbenzenesulfonamides. All sulfonamide derivatives have confirmed in vitro anticancer activities against human colon cancer (HCT-116) expressed as pIC50 and shown in Table 2.

#### 3.1.2. The Analytical Standards

The model substances were utilized to obtain the chromatographic hydrophobicity index of IAM (CHI_IAM_) and C_18_ (CHI_C18_). The analytical standards of acetaminophen, acetophenone, benzimidazole, colchicine, indole, and theophylline were purchased from Sigma-Aldrich (Steinheim, Germany). Octanonophenone, butyrophenone, and acetanilide were obtained from Alfa Aesar (Haverhill, MA, USA). Heptanophenone, hexanophenone, valerophenone, propiophenone, and acetophenone were bought from Acros Organic (Massachusetts, MA, USA).

#### 3.1.3. Reagents

The water (18.2 MΩ × cm^−1^) was purified and deionized in our laboratory via a Millipore Direct-Q 3 UV Water Purification System (Millipore Corporation, Bedford, MA, USA) to prepare the mobile phase. Ammonium acetate, disodium phosphate (Na_2_HPO_4_), and monosodium phosphate (NaH_2_PO_4_) were purchased from POCH (Gliwice, Poland). Dimethyl sulfoxide (DMSO), used as a solvent, was from Merck (Darmstadt, Germany). Acetonitrile and 2-propanol (LiChrosolv^®^) gradient grade for liquid chromatography was purchased from Sigma-Aldrich (Steinheim, Germany). 

### 3.2. Chromatographic Systems

All HPLC experiments were carried out using a Prominence-1 LC-2030C 3D HPLC system (Shimadzu, Japan) equipped with a DAD detector and controlled by the LabSolution system (version 5.90 Shimadzu, Japan). The stock solutions of solutes were diluted to obtain 100 μg/mL concentrations, and the injected volume was 10 μL. During this study, six different columns in terms of chemical modification of stationary phases were used. Retention times (t_R_) of investigated sulfonamides were collected, and their detection in all systems was performed at the wavelength characteristic for each compound, summarized in Table S1. For all chromatography systems, analysis was carried out at 40 °C, except for IAM chromatography, in which oven temperature was set to 30 °C. The CHI_IAM_ and CHI_18_ indices of the target sulfonamides derivatives were obtained using the protocol proposed by Valko and co-workers [50]. C_18_ chromatography was performed on a Waters-C_18_ column (150 mm × 3.9 mm; 5.0 µm; Symmetry; USA), with a 1.5 mL/min flow rate. The mobile phase was ammonium acetate buffer (50 mM) at pH 7.4 and acetonitrile as an organic modifier. The linear gradient from 2 to 98% ACN was applied from 0 to 30 min. IAM chromatography was executed on an IAM.PC.DD2 column (100 cm × 4.6 mm; 10.0 µm; Regis Technologies, Morton Grove, IL, USA) additionally equipped with an IAM guard column with the same flow rate of 1.5 mL/min. The mobile phase was sodium phosphate buffer (10 mM) at pH 7.4 and acetonitrile as the organic phase. The linear gradient from 0 to 85% was applied within 5.25 min. 

**Figure 9:**
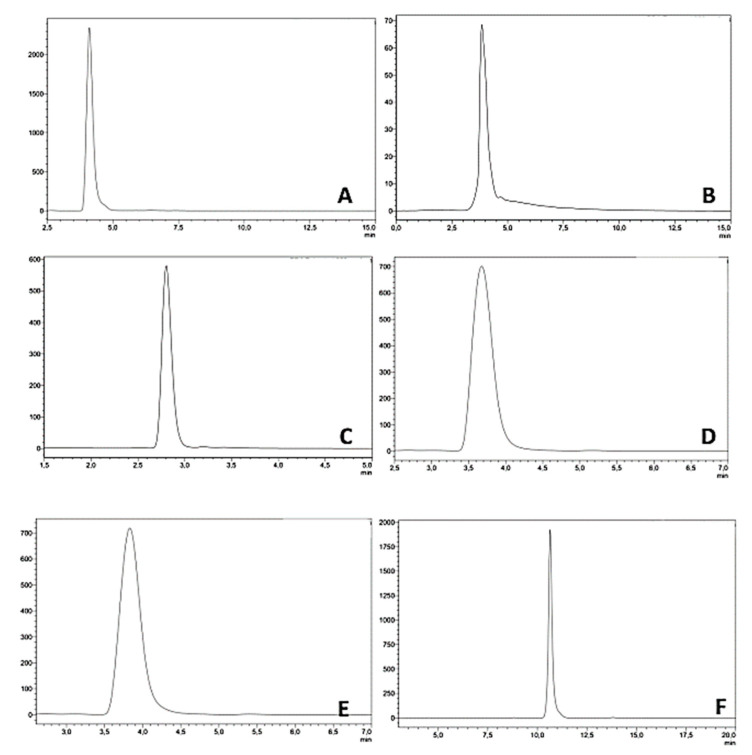
Representative chromatograms for molecule 1 achieved in investigated HPLC systems: (**A**) C_8_, (**B**) CN, (**C**) IAM, (**D**) Ph (long gradient), (**E**) Ph (short gradient), (**F**) C_18_. The detailed experimental conditions are reported in Section 3.2.

For C_8_, cyanopropyl, and phenyl chromatography, according to the assumption proposed by Snyder and co-workers [42,43], appropriate log*k*_w_ values (i.e., the retention factor log*k* extrapolated to 0% organic modifier, as an alternative to log*P*) were obtained. Two retention times for each system in two different gradients (short and long) were collected, and these data, as input, were introduced into DryLab 6.0 software (Molnar Institute, Berlin, Germany). Dwell volume for these HPLC systems was measured at 0.780 mL, whereas the obtained dead times for used HPLC columns were equal to 1.530 min, 1.252 min, and 2.335 min for C_8_, SB-CN, and UK-Phenyl, respectively. The mobile phase was ammonium acetate buffer (50 mM) at pH 7.4 and acetonitrile as an organic modifier in each system. C_8_ chromatography was performed on Unison UK-C8 column (150 × 2 mm; 3.0 µm. Imtakt; USA) with 0.3 mL/min flow rate. Analyses were carried out in linear gradient from 30 to 100% within 15 min in short gradient and 30 min in long gradient. The Agilent SB-CN column (150 × 4.6 mm; 3.5 µm; Zorbax; USA) was used for the cyanopropyl chromatography. The CN chromatography analyses were carried out in linear gradient from 20 to 100%, which was applied from 0 to 20 min in short gradient, and from 0 to 40 min in long gradient. The flow rate was 1.5 mL/min. The column Unison UK-Phenyl (150 × 2 mm; 3.0 µm; Imtakt; USA) was used to perform phenyl chromatography. The flow rate was set to 0.2 mL/min. The phenyl analyses were carried out in linear gradient from 40 to 100%, which was applied from 0 to 20 min in short gradient, and from 0 to 40 min in long gradient. Each HPLC analysis was run in triplicate; in Tables S2–S6, obtained retention times are listed, whereas representative chromatograms are shown in Figure 9.

### 3.3. In Silico Calculation

#### 3.3.1. Theoretical Descriptors 

The theoretical descriptors were calculated applying alvaDesc software [51] and based on geometries optimization using universal force field (UFF) via OpenBabel software [52]. Before QSRR analysis, constant and near-constant were removed. MolGpka was applied for the calculation of pKa (https://xundrug.cn/molgpka, accessed on 1 March 2022). Finally, 3352 descriptors belonging to 31 classes were calculated.

#### 3.3.2. In Silico Calculation of Lipophilicity

SwissADME software (http://www.swissadme.ch, accessed on 1 March 2022) and KOWWIN v. 1.68 software (EPI Suite package v.4.2, U.S. EPA) were used for lipophilicity calculations based on the SMLIE notation. Calculated lipophilicity indexes are summarized in Table 1.

### 3.4. Data Analysis

#### 3.4.1. Cluster Analysis (CA) and Principal Component Analysis (PCA)

CA and PCA were performed on databases that included chromatographic data and in-silico-calculated lipophilicity indices. In order to eliminate the impact of various lipophilicity scales, data were standardized before analysis. Using Ward’s agglomeration rule and the Euclidian distance measure, CA has presented results as clustered heat maps. Both PCA and CA analysis and visualization were performed using Python scripts. 

#### 3.4.2. Sum of Ranking Differences (SRD) Analysis

The SRD analysis, introduced by Héberger [53,54,55], was carried out on the standardized lipophilicity data to rank, group, and select the most suitable lipophilicity measures of the studied series of sulfonamide derivatives obtained by in silico and experimental (chromatographic) approaches. The analysis was performed by using a data matrix in which the lipophilicity measures are organized in the columns while the compounds are listed in the rows. The last column contained average row values used as a reference ranking. This so-called “consensus approach” measures the differences from the center as a non-parametric measure of similarities and dissimilarities [55]. The results of the ranking are interpreted based on the SRD values. The best objects (models, descriptors, molecules, etc.) are the ones that have the SRD values equal to or closer to zero (the objects closest to the reference ranking, i.e., “golden standard”) [55]. The validation of the SRD procedure was done by comparison of rank by random numbers (CRRN) and 7-fold cross-validation based on omitting about 1/7 of objects and carrying out the ranking on the rest of the objects [54,55]. The normalized SRD values (SRD%) were used to compare results from different SRD analyses.

#### 3.4.3. QSRR Analysis

Descriptors selection was supported by a genetic algorithm (GA), whereas multiple linear regression (PLS) was employed as a regression method. The QSRR models were built using the retention data and the calculated descriptors as dependent and independent variables, respectively. The set of parameters applied to control GA was the size of the population (500) and the mutation rate (0.1). The models were built using three LVs and five structural descriptors in each variable. The optimization of the models was carried out based on R^2^. Before calculating GA-PLS for each modeled endpoint, the target solutes were randomly divided into the training group (*n* = 18) and the testing group (*n* = 9). The training set always contains the molecule with the highest and lowest value of the modeled endpoint. The information on the belonging of each compound to a training or testing set is included in Figure S1. 

The following statistical figures were used for assessment of model fitting and predictive abilities: the coefficient of determination (*R^2^*), external validation coefficients (*Q_F1_^2^, Q_F2_^2^, Q_F3_^2^*), root-mean-squared error of cross-validation (RMSE_CV_), root-mean-square error in prediction (RMSE_P_) and concordance correlation coefficient (CCC). Applied statistics were calculated using the following formulas:

(3) $$R^{2}=1-\frac{\sum_{i=1}^{n_{TR}}{\left(y_{i}{}^{obs}-y_{i}{}^{pred}\right)}^{2}}{\sum_{i=1}^{n_{TR}}{\left(y_{i}{}^{obs}-{\bar{y}}_{TR}^{obs}\right)}^{2}}$$

(4) $$Q_{F1}{}^{2}=1-\frac{\sum_{i=1}^{n_{EXT}}{\left(y_{i}{}^{obs}-y_{i}{}^{pred}\right)}^{2}}{\sum_{i=1}^{n_{EXT}}{\left(y_{i}{}^{obs}-{\bar{y}}_{TR}^{obs}\right)}^{2}}$$

(5) $$Q_{F2}{}^{2}=1-\frac{\sum_{i=1}^{n_{EXT}}{\left(y_{i}{}^{obs}-y_{i}{}^{pred}\right)}^{2}}{\sum_{i=1}^{n_{EXT}}{\left(y_{i}{}^{obs}-{\bar{y}}_{EXT}^{obs}\right)}^{2}}$$

(6) $$Q_{F3}{}^{2}=1-\frac{\left[\sum_{i=1}^{n_{EXT}}{\left(y_{i}{}^{obs}-y_{i}{}^{pred}\right)}^{2}\right]/n_{EXT}}{\left[\sum_{i=1}^{n_{EXT}}{\left(y_{i}{}^{obs}-{\bar{y}}_{TR}^{obs}\right)}^{2}\right]/n_{TR}}$$

(7) $$RMSE_{P}=\sqrt{\frac{\sum_{i=1}^{n_{EXT}}{\left(y_{i}{}^{obs}-y_{i}{}^{pred}\right)}^{2}}{n_{EXT}}}$$

(8) $$CCC=\;\frac{2\;\sum_{i=1}^{n_{EXT}}\left(y_{i}{}^{obs}-{\bar{y}}_{EXT}^{obs}\right)\left(y_{i}{}^{pred}-{\bar{y}}_{EXT}^{pred}\right)}{\sum_{i=1}^{n_{EXT}}{\left(y_{i}{}^{obs}-{\bar{y}}_{EXT}^{obs}\right)}^{2}+\;\sum_{i=1}^{n_{EXT}}{\left(y_{i}{}^{pred}-{\bar{y}}_{EXT}^{pred}\right)}^{2}+\;n_{EXT}{\left({\bar{y}}_{EXT}^{obs}-{\bar{y}}_{EXT}^{pred}\right)}^{2}}$$

The same procedures were used to calculate the quantitative structure–activity relationship model, where the pIC_50_ was used as the independent variable, and the dependent variables were lipophilicity parameters, both computational and chromatographic, and the remaining structural descriptors obtained.

## 4. Conclusions

Both PCA and CA showed significant differences between the chromatographically determined lipophilicity and calculated ones. Although the grouping results can be explained by ionization, since the calculation methods do not include information about the ionization of the compounds all the time, significant differences between the values calculated by different algorithms affect the credibility of the results obtained through the computational approach. SRD indicated that among the chromatographic lipophilicity parameters of the analyzed sulfonamide derivatives, log*k*_IAM_ could be considered the best choice. Consequently, IAM-HPLC can be recommended as the most sustainable method for the lipophilicity characterization of this class of chemical compounds. From the practical point of view, considering the ratio between lipophilicity and anticancer activity, according to the assumption that the compound should be characterized by the lowest lipophilicity while maintaining pharmacological action for different tested molecules, compounds **1**–**4** and **26** can be recommended.

## Figures and Tables

**Table 1:** The calculated log*P* values of the sulfonamides derivatives concerning the computational model.

No.	iLog*P*	XLog*P*3	WLog*P*	MLog*P*	Silicos-IT Log*P*	Consensus Log*P*	Log*P* KOWWIN
1	2.12	1.22	3.27	0.58	0.97	1.63	1.78
2	2.59	2.15	4.72	2.04	2.02	2.70	4.23
3	2.82	3.48	5.02	2.27	2.53	3.23	4.78
4	2.95	3.51	5.02	2.27	2.53	3.26	4.78
5	2.41	2.71	4.17	1.23	2.11	2.53	3.44
6	2.52	3.79	4.82	2.58	2.20	3.18	3.93
7	2.74	1.36	3.57	0.83	1.47	2.00	2.32
8	2.56	5.82	7.80	3.93	5.83	5.19	7.38
9	2.05	4.53	5.42	3.24	4.72	3.99	6.38
10	2.35	6.19	6.78	4.06	5.74	5.03	7.59
11	2.68	5.31	5.93	3.68	5.62	4.64	7.61
12	2.13	5.14	5.84	2.88	3.49	3.89	5.23
13	3.29	4.81	6.89	3.85	5.01	4.77	5.02
14	3.18	5.15	7.09	3.44	4.85	4.74	5.06
15	3.71	6.69	9.91	4.68	6.53	6.30	6.58
16	4.15	5.78	7.75	3.90	5.50	5.42	5.70
17	3.78	6.43	9.56	4.42	6.42	6.12	6.49
18	4.10	5.52	7.40	3.63	5.39	5.21	5.61
19	4.28	5.46	7.42	3.00	5.57	5.15	5.04
20	3.43	5.12	7.10	3.13	4.94	4.74	5.14
21	3.37	6.48	10.67	4.75	6.79	6.41	6.74
22	4.04	6.06	8.04	4.48	6.02	5.73	6.19
23	3.30	5.93	7.49	3.78	5.67	5.24	6.07
24	3.58	5.70	9.06	4.36	6.11	5.76	5.98
25	3.70	5.57	8.51	3.66	5.76	5.44	5.86
26	2.39	4.92	5.85	3.55	4.61	4.27	4.99
27	3.18	7.00	7.43	4.73	6.31	5.73	6.97

**Table 2:** The summarized values of log*k_w_,* CHI_C1*8*_*,* CHI_IAM_, pKa, and pIC_50_ for sulfonamides derivatives.

No.	CHI_IAM_	log*k*_IAM_	CHI_C18_	CHI log*D*	log_kw_C_8_	log*k*_w_CN	log*k*_w_Ph	pKa	pIC_50_
1	19.20	1.28	56.70	1.51	2.98	1.58	2.62	6.6	4.34
2	45.00	2.45	97.50	3.65	4.68	4.04	6.22	7.3	6.00
3	42.00	2.31	94.80	3.51	4.59	2.97	5.56	7.4	4.72
4	45.90	2.49	104.80	4.04	4.90	3.63	7.39	7.3	5.05
5	31.40	1.83	82.90	2.89	4.06	2.30	4.08	6.5	4.64
6	45.40	2.46	94.70	3.50	4.61	2.89	5.39	7.8	4.15
7	24.30	1.51	63.80	1.88	3.30	1.78	2.81	6.9	4.16
8	36.70	2.07	82.80	2.88	4.21	2.01	4.49	5.5	4.10
9	31.40	1.83	72.30	2.33	3.69	1.85	2.88	5.4	4.07
10	39.10	2.18	84.30	2.96	4.21	2.25	4.19	5.5	4.05
11	35.50	2.02	80.30	2.75	4.07	2.13	3.82	5.5	4.06
12	35.20	2.00	79.30	2.70	4.07	2.16	3.90	5.4	4.06
13	37.10	2.09	77.90	2.62	4.03	2.04	3.53	5.7	4.12
14	35.80	2.03	78.90	2.68	4.13	2.02	4.31	5.1	4.11
15	43.40	2.37	92.50	3.39	4.53	2.64	5.07	5.1	4.19
16	39.80	2.21	85.90	3.04	4.38	2.47	4.48	5.1	4.12
17	41.40	2.28	89.90	3.25	4.49	2.54	4.54	5.1	4.15
18	37.50	2.11	82.80	2.88	4.28	2.36	4.23	5.1	4.12
19	33.60	1.93	79.70	2.72	4.07	2.22	3.97	5.0	4.12
20	34.90	1.99	77.20	2.59	4.02	2.21	3.79	5.1	4.14
21	41.30	2.28	91.60	3.34	4.44	2.11	4.48	5.7	4.29
22	42.20	2.32	84.60	2.97	4.28	2.36	4.25	5.7	4.31
23	41.10	2.27	86.10	3.05	4.12	2.48	4.40	5.8	4.42
24	38.30	2.14	87.50	3.13	4.26	2.28	4.09	5.7	4.17
25	37.20	2.09	84.40	2.96	4.26	2.33	3.89	5.7	4.17
26	30.60	1.80	84.10	2.95	3.66	1.76	2.87	5.2	4.85
27	39.60	2.20	73.10	2.37	4.28	2.31	4.46	4.6	4.11

**Table 3:** The calculated statistical figures of the obtained GA-PLS QSRR and QSAR models.

	R^2^	Q^2^loo	RMSE_cv_	RMSE_ext_	Q^2^ _F1_	Q^2^ _F2_	Q^2^ _F3_	CCC
log*k*_IAM_	0.946	0.860	0.070	0.075	0.877	0.860	0.938	0.927
CHI logD	0.941	0.810	0.136	0.173	0.828	0.810	0.904	0.884
log*kw*_C8_	0.966	0.918	0.081	0.084	0.927	0.918	0.963	0.964
log*kw*_CN_	0.973	0.877	0.086	0.181	0.881	0.877	0.879	0.937
log*kw*_Ph_	0.937	0.861	0.264	0.337	0.865	0.861	0.898	0.924
pIC_50_	0.976	0.858	0.070	0.120	0.858	0.858	0.933	0.929

## Data Availability

All data are available as Supplementary Materials.

## Supplementary Materials

The following supporting information can be downloaded at: https://www.mdpi.com/article/10.3390/molecules27133965/s1, Table S1. The SMILES of the studied sulfonamide derivatives and their chemical class of substituents and IC_50_ values; Table S2. The obtained retention times together with CHI_IAM_ for the target sulfonamides derivatives in IAM chromatography; Table S3. The obtained retention times and CHI_C18_ for the target sulfonamide derivatives in C_18_ chromatography; Table S4. The obtained retention times and log_kw_ for the target sulfonamide derivatives in C_8_ chromatography; Table S5. The obtained retention times and log_kw_ for the target sulfonamide derivatives in Cyanopropyl chromatography; Table S6. The obtained retention times and log_kw_ for the target sulfonamide derivatives in phenyl chromatography; Table S7. List of molecular descriptors used to build QSRR models.; Table S8. The values of theoretical descriptors for each compound; Figure S1. William’s plots and y-randomization tests (Q2 vs. R2) generated for each model. The numbers correspond to the next stationary phases A) IAM. B) C18. C) C8. D) CN. E) Ph and F) pIC50.
