# Supplementary material for: Modeling of Anticancer Sulfonamide Derivatives Lipophilicity by Chemometric and Quantitative Structure-Retention Relationships Approaches
Source: Molecules. 2022 Jun 21;27(13):3965. doi: 10.3390/molecules27133965 (PMC9268166; doi:10.3390/molecules27133965)
Supplement: Supplementary file 1 [file molecules-27-03965-s001.zip › molecules-1759529-supplementary.pdf]

Supplementary materials

Modeling of anticancer sulfonamide derivatives lipophilicity by chemometric and quantitative structure-retention relationships approaches

Monika Pastewska[1]. Beata Żołnowska[2]. Strahinja Kovačević[3]. Hanna Kapica[1]. Maciej Gromelski[4]. Filip Stoliński[4,5]. Jarosław Sławiński[2]. Wiesław Sawicki[1]. Krzesimir Ciura[1,4*]

[1] Department of Physical Chemistry. Medical University of Gdańsk. Al. Gen. Hallera 107. 80-416 Gdańsk. Poland

[2] Department of Organic Chemistry. Medical University of Gdańsk. Al. Gen. Hallera 107. 80-416 Gdańsk. Poland

[3] University of Novi Sad. Faculty of Technology Novi Sad. Department of Applied and Engineering Chemistry. Bulevar cara Lazara 1. 21000 Novi Sad. Serbia

[4] QSAR Lab Ltd.. Trzy Lipy 3 St. Gdańsk. 80-172. Poland

[5] University of Gdansk. Faculty of Chemistry. Laboratory of Environmental Chemoinformatics. Wita Stwosza 63. 80-308 Gdansk. Poland

* Correspondence: k.ciura@qsarlab.pl

Table S1. The SMILES of the studied sulfonamide derivatives together with their chemical class of substituents and maxium of absorbance.

No	Substituent's chemical class	Smiles	max abs.
**1**	pyrrole	O=S(NC1=NC=CC(C)=N1)(C2=CC=C(N3C=CC=C3)C=C2)=O	244
**2**	pyrrole	O=S(C1=CC=C(N2C=CC=C2)C=C1)(NC3=C4C(C=CC=N4)=CC=C3)=O	237
**3**	pyrrole	O=S(C1=CC=C(N2C=CC=C2)C=C1)(NC3=C4C(C=CC=N4)=CC=C3C)=O	280
**4**	pyrrole	O=S(C1=CC=C(N2C=CC=C2)C=C1)(NC3=C4C(C=CC(C)=N4)=CC=C3)=O	239
**5**	pyrrole	O=S(NC1=CC=CC2=NSN=C21)(C3=CC=C(N4C=CC=C4)C=C3)=O	248
**6**	pyrrole	ClC1=CC=C(NS(C2=CC=C(N3C=CC=C3)C=C2)(=O)=O)C=C1	278
**7**	pyrrole	O=S(NC1=NC(C)=CC(C)=N1)(C2=CC=C(N3C=CC=C3)C=C2)=O	244
**8**	5-oxo-4.5-dihydro-1.2.4-triazine	ClC1=C(C)C=C(S(NC2=NN=C(C3=CC=CC=C3)C(N2)=O)(=O)=O)C(SCC4=CC=C(C(F)(F)F)C=C4)=C1	224
**9**	5-oxo-4.5-dihydro-1.2.4-triazine	ClC1=C(C)C=C(S(NC2=NN=C(C)C(N2)=O)(=O)=O)C(SCC3=CC=CC4=C3C=CC=C4)=C1	221
**10**	5-oxo-4.5-dihydro-1.2.4-triazine	ClC1=C(C)C=C(S(NC2=NN=C(C3=CC=CC=C3)C(N2)=O)(=O)=O)C(SCC4=CC=CC5=C4C=CC=C5)=C1	222
**11**	5-oxo-4.5-dihydro-1.2.4-triazine	ClC1=C(C)C=C(S(NC2=NN=C(CC3=CC=CC=C3)C(N2)=O)(=O)=O)C(SCC4=C(Cl)C=C(OCO5)C5=C4)=C1	194
**12**	5-oxo-4.5-dihydro-1.2.4-triazine	ClC1=C(C)C=C(S(NC2=NN=C(CC3=C([N+]([O-])=O)C=CC=C3)C(N2)=O)(=O)=O)C(SCC4=C(Cl)C=C(OCO5)C5=C4)=C1	230
**13**	1.2.4-triazine	ClC1=C(C)C=C(S(NC2=NN=CC(C3=CC=CC(F)=C3)=N2)(=O)=O)C(SCC4=CC=CC=C4)=C1	194
**14**	1.2.4-triazine	ClC1=C(C(NC2=CC=CC=C2)=O)C=C(S(NC3=NN=CC(C4=CC=C(OC)C=C4)=N3)(=O)=O)C(SCC5=CC=CC=C5)=C1	194
**15**	1.2.4-triazine	ClC1=C(C(NC2=CC=C(Cl)C=C2)=O)C=C(S(NC3=NN=CC(C4=CC=C(C(F)(F)F)C=C4)=N3)(=O)=O)C(SCC5=CC=CC=C5)=C1	195
**16**	1.2.4-triazine	ClC1=C(C(NC2=CC=C(Cl)C=C2)=O)C=C(S(NC3=NN=CC(C4=CC=C(OC)C=C4)=N3)(=O)=O)C(SCC5=CC=CC=C5)=C1	193
**17**	1.2.4-triazine	ClC1=C(C(NC2=CC=C(C)C=C2)=O)C=C(S(NC3=NN=CC(C4=CC=C(C(F)(F)F)C=C4)=N3)(=O)=O)C(SCC5=CC=CC=C5)=C1	268
**18**	1.2.4-triazine	ClC1=C(C(NC2=CC=C(C)C=C2)=O)C=C(S(NC3=NN=CC(C4=CC=C(OC)C=C4)=N3)(=O)=O)C(SCC5=CC=CC=C5)=C1	194
**19**	1.2.4-triazine	ClC1=C(C(NC2=CC=C(C)C=C2)=O)C=C(S(NC3=NN=CC(C4=CC(OC)=C(OC)C(OC)=C4)=N3)(=O)=O)C(SCC5=CC=CC=C5)=C	194
**20**	1.2.4-triazine	ClC1=C(C(NC2=CC=C(OC)C=C2)=O)C=C(S(NC3=NN=CC(C4=CC=C(OC)C=C4)=N3)(=O)=O)C(SCC5=CC=CC=C5)=C1	238
**21**	1.2.4-triazine	ClC1=C(C)C=C(S(NC2=NN=CC(C3=CC=C(C(F)(F)F)C=C3)=N2)(=O)=O)C(SCC4=CC=C(C(F)(F)F)C=C4)=C1	193
**22**	1.2.4-triazine	ClC1=C(C)C=C(S(NC2=NN=CC(C3=CC(F)=CC=C3)=N2)(=O)=O)C(SCC4=CC=CC5=C4C=CC=C5)=C1	222
**23**	1.2.4-triazine	ClC1=C(C)C=C(S(NC2=NN=CC(C3=CC=C(OC)C=C3)=N2)(=O)=O)C(SCC4=CC=CC5=C4C=CC=C5)=C1	222
**24**	1.2.4-triazine	ClC1=C(C)C=C(S(NC2=NN=CC(C3=CC(F)=CC=C3)=N2)(=O)=O)C(SCC4=CC=C(C(F)(F)F)C=C4)=C1	194
**25**	1.2.4-triazine	ClC1=C(C)C=C(S(NC2=NN=CC(C3=CC=C(OC)C=C3)=N2)(=O)=O)C(SCC4=CC=C(C(F)(F)F)C=C4)=C1	221
**26**	N-acyl	ClC1=C(C)C=C(S(NC(C)=O)(=O)=O)C(SCC2=CC=CC3=C2C=CC=C3)=C1	222
**27**	N-acyl	ClC1=C(C)C=C(S(NC(/C=C/C2=CC=CC=C2)=O)(=O)=O)C(SCC3=CC=CC4=C3C=CC=C4)=C1	223

Table S2. The obtained retention times together with CHI_{IAM} for the target sulfonamides derivatives in IAM chromatography

No	tr_1	tr_2	tr_3	t_m	CHI	SD
**1**	2.823	2.801	2.809	2.811	19.2	0.01
**2**	4.467	4.463	4.559	4.496	45.0	0.05
**3**	4.292	4.308	4.315	4.305	42.0	0.01
**4**	4.539	4.569	4.571	4.560	45.9	0.02
**5**	3.599	3.610	3.612	3.607	31.4	0.01
**6**	4.520	4.530	4.526	4.525	45.4	0.01
**7**	3.146	3.148	3.147	3.147	24.3	0.01
**8**	3.952	3.952	3.963	3.956	36.7	0.01
**9**	3.606	3.613	3.612	3.610	31.4	0.01
**10**	4.115	4.118	4.103	4.112	39.1	0.01
**11**	3.88	3.878	3.882	3.880	35.5	0.01
**12**	3.856	3.856	3.856	3.856	35.2	0.01
**13**	3.973	3.981	3.983	3.979	37.1	0.01
**14**	3.891	3.888	3.907	3.895	35.8	0.01
**15**	4.388	4.399	4.397	4.395	43.4	0.01
**16**	4.163	4.159	4.158	4.160	39.8	0.01
**17**	4.270	4.258	4.266	4.265	41.4	0.01
**18**	4.015	4.005	4.005	4.008	37.5	0.01
**19**	3.753	3.748	3.755	3.752	33.6	0.01
**20**	3.842	3.836	3.830	3.836	34.9	0.01
**21**	4.261	4.249	4.257	4.256	41.3	0.01
**22**	4.315	4.310	4.317	4.314	42.2	0.01
**23**	4.235	4.248	4.252	4.245	41.1	0.01
**24**	4.062	4.056	4.060	4.059	38.3	0.01
**25**	3.977	4.004	3.986	3.989	37.2	0.01
**26**	3.555	3.560	3.558	3.558	30.6	0.01
**27**	4.146	4.149	4.144	4.146	39.6	0.01

Table S3. The obtained retention times together with CHI_{C18} for the target sulfonamides derivatives in C_{18} chromatography

No	tr_1	tr_2	tr_3	t_m	CHI	SD
**1**	10.645	10.610	10.610	10.622	56.7	0.02
**2**	18.690	18.746	18.762	18.733	97.5	0.04
**3**	18.247	18.185	18.176	18.203	94.8	0.04
**4**	20.098	20.274	20.225	20.199	104.8	0.09
**5**	15.857	15.830	15.818	15.835	82.9	0.02
**6**	18.208	18.178	18.147	18.178	94.7	0.03
**7**	12.020	12.014	12.041	12.025	63.8	0.01
**8**	15.826	15.806	15.778	15.803	82.8	0.02
**9**	13.738	13.722	13.677	13.712	72.3	0.03
**10**	16.152	16.129	16.073	16.118	84.3	0.04
**11**	15.361	15.338	15.259	15.319	80.3	0.05
**12**	15.154	15.105	15.070	15.110	79.3	0.04
**13**	14.888	14.856	14.768	14.837	77.9	0.06
**14**	15.090	15.040	14.951	15.027	78.9	0.07
**15**	17.836	17.774	17.643	17.751	92.5	0.10
**16**	16.502	16.453	16.309	16.421	85.9	0.10
**17**	17.307	17.259	17.087	17.218	89.9	0.12
**18**	15.837	15.791	15.787	15.805	82.8	0.03
**19**	15.203	15.176	15.175	15.185	79.7	0.02
**20**	14.730	14.690	14.658	14.693	77.2	0.04
**21**	17.585	17.546	17.531	17.554	91.6	0.03
**22**	16.065	16.221	16.230	16.172	84.6	0.09
**23**	16.482	16.450	16.469	16.467	86.1	0.02
**24**	16.797	16.650	16.773	16.740	87.5	0.08
**25**	16.134	16.127	16.144	16.135	84.4	0.01
**26**	16.168	16.144	15.895	16.069	84.1	0.15
**27**	13.870	13.864	13.936	13.890	73.1	0.04

Table S4. The obtained retention times together with $\log_{kw}$ for the target sulfonamides derivatives in C_8 chromatography

No	t_1 short grad.	t_2 short grad.	t_3 short grad.	SD	t_m short grad.	t_1 long grad.	t_2 long grad.	t_3 long grad.	SD	t_m long grad.	$\log_{kw}$
**1**	4.411	4.106	4.117	0.17	4.211	4.260	4.136	4.142	0.07	4.179	2.980
**2**	10.053	10.081	10.092	0.02	10.075	13.819	13.814	13.924	0.06	13.852	4.682
**3**	9.789	9.808	9.823	0.02	9.807	13.302	13.317	13.358	0.03	13.326	4.593
**4**	10.721	10.779	10.800	0.04	10.767	15.131	15.120	15.163	0.02	15.138	4.898
**5**	8.106	8.144	8.144	0.02	8.131	10.096	10.102	10.157	0.03	10.118	4.055
**6**	9.804	9.811	9.829	0.01	9.815	13.400	13.403	13.452	0.03	13.418	4.609
**7**	5.411	5.414	5.418	0.00	5.414	5.768	5.795	5.830	0.03	5.798	3.300
**8**	8.327	8.315	8.293	0.02	8.312	11.004	11.035	11.027	0.02	11.022	4.206
**9**	6.656	6.628	6.628	0.02	6.637	7.948	7.944	7.986	0.02	7.959	3.692
**10**	8.335	8.336	8.325	0.01	8.332	11.012	11.078	11.067	0.04	11.052	4.211
**11**	7.901	7.854	7.865	0.02	7.873	10.219	10.200	10.280	0.04	10.233	4.074
**12**	7.886	7.850	7.839	0.02	7.858	10.208	10.195	10.234	0.02	10.212	4.070
**13**	7.577	7.548	7.538	0.02	7.554	9.630	9.630	9.630	0.00	9.630	4.030
**14**	7.738	7.710	7.702	0.02	7.717	9.955	9.952	9.964	0.01	9.957	4.130
**15**	9.489	9.464	9.456	0.02	9.470	13.327	13.294	13.305	0.02	13.309	4.530
**16**	8.545	8.521	8.513	0.02	8.526	11.518	11.510	11.521	0.01	11.516	4.380
**17**	9.163	9.134	9.113	0.03	9.137	12.717	12.704	12.711	0.01	12.711	4.490
**18**	8.170	8.150	8.144	0.01	8.155	10.803	10.796	10.814	0.01	10.804	4.280
**19**	7.837	7.822	7.824	0.01	7.828	10.189	10.204	10.194	0.01	10.196	4.068
**20**	7.547	7.536	7.515	0.02	7.533	9.582	9.595	9.586	0.01	9.588	4.020
**21**	9.364	9.350	9.329	0.02	9.348	13.057	13.048	13.057	0.01	13.054	4.440
**22**	8.519	8.506	8.488	0.02	8.504	11.436	11.440	11.441	0.00	11.439	4.276
**23**	8.577	8.556	8.560	0.01	8.564	11.493	11.498	11.509	0.01	11.500	4.120
**24**	8.439	8.420	8.408	0.02	8.422	11.319	11.308	11.320	0.01	11.316	4.256
**25**	8.385	8.365	8.358	0.01	8.369	11.182	11.184	11.197	0.01	11.188	4.260
**26**	6.558	6.559	6.543	0.01	6.553	7.732	7.745	7.744	0.01	7.740	3.655
**27**	8.545	8.537	8.539	0.00	8.540	11.444	11.457	11.460	0.01	11.454	4.279

Table S5. The obtained retention times together with $\log_{kw}$ for the target sulfonamides derivatives in Cyanopropyl chromatography

No.	t_1 short grad.	t_2 short grad.	t_3 short. grad.	SD	t_m short grad.	t_1 long grad.	t_2 long grad.	t_3 long grad.	SD	t_m long grad.	$\log_{kw}$
**1**	2.481	2.494	2.473	0.01	2.483	3.625	3.842	3.866	0.13	3.778	1.580
**2**	8.571	9.049	9.219	0.34	8.946	17.815	17.875	17.920	0.05	17.870	4.040
**3**	6.840	6.889	6.906	0.03	6.878	13.381	13.346	13.317	0.03	13.348	2.966
**4**	8.675	8.749	8.781	0.05	8.735	17.187	17.238	17.224	0.03	17.216	3.628
**5**	5.011	5.000	4.983	0.01	4.998	9.524	9.473	9.365	0.08	9.454	2.300
**6**	6.698	6.692	6.665	0.02	6.685	12.972	12.925	12.847	0.06	12.915	2.892
**7**	3.046	3.101	3.111	0.04	3.086	5.438	5.420	5.369	0.04	5.409	1.780
**8**	3.962	3.935	3.924	0.02	3.940	8.839	8.766	8.726	0.06	8.777	2.010
**9**	3.004	2.980	2.968	0.02	2.984	6.559	6.659	6.452	0.10	6.557	1.850
**10**	4.170	4.129	4.118	0.03	4.139	9.231	9.158	9.113	0.06	9.167	2.251
**11**	3.822	3.773	3.762	0.03	3.786	8.523	8.451	8.408	0.06	8.461	2.130
**12**	3.906	3.841	3.841	0.04	3.863	8.708	8.653	8.594	0.06	8.652	2.162
**13**	3.581	3.545	3.535	0.02	3.554	7.986	7.905	7.859	0.06	7.917	2.037
**14**	4.075	4.050	4.038	0.02	4.054	9.032	9.001	8.986	0.02	9.006	2.020
**15**	5.301	5.232	5.219	0.04	5.251	11.505	11.455	11.399	0.05	11.453	2.642
**16**	4.830	4.775	4.758	0.04	4.788	10.515	10.454	10.399	0.06	10.456	2.471
**17**	4.976	4.937	4.921	0.03	4.945	10.929	10.879	10.830	0.05	10.879	2.544
**18**	4.475	4.440	4.424	0.03	4.446	9.858	9.802	9.728	0.07	9.796	2.358
**19**	4.011	3.975	3.969	0.02	3.985	9.012	8.962	8.912	0.05	8.962	2.216
**20**	4.015	3.990	3.976	0.02	3.994	8.983	8.930	8.891	0.05	8.935	2.211
**21**	4.863	4.825	4.813	0.03	4.834	10.694	10.643	10.594	0.05	10.644	2.110
**22**	4.451	4.423	4.417	0.02	4.430	9.844	9.772	9.724	0.06	9.780	2.356
**23**	4.898	4.869	4.871	0.02	4.879	10.570	10.488	10.430	0.07	10.496	2.478
**24**	4.224	4.195	4.180	0.02	4.200	9.370	9.329	9.262	0.05	9.320	2.277
**25**	4.434	4.411	4.397	0.02	4.414	9.676	9.647	9.560	0.06	9.628	2.330
**26**	3.027	3.013	3.012	0.01	3.017	6.362	6.336	6.252	0.06	6.317	1.763
**27**	4.359	4.333	4.309	0.03	4.334	9.601	9.543	9.453	0.07	9.532	2.313

Table S6. The obtained retention times together with $\log_{kw}$ for the target sulfonamides derivatives in Phenyl chromatography

No	t_1 short grad.	t_2 short grad.	t_3 short grad.	SD	t_m	t_1 long grad.	t_2 long grad.	t_3 long grad.	SD	t_m	logkw
**1**	3.830	3.777	3.774	0.03	3.794	3.670	3.692	3.675	0.01	3.679	2.620
**2**	11.023	11.390	11.544	0.27	11.319	13.971	14.045	13.878	0.08	13.965	6.221
**3**	10.568	10.453	10.455	0.07	10.492	12.130	12.058	12.031	0.05	12.073	5.556
**4**	12.356	13.281	13.453	0.59	13.030	17.414	17.332	17.118	0.15	17.288	7.389
**5**	7.813	7.748	7.731	0.04	7.764	7.908	7.860	7.842	0.03	7.870	4.079
**6**	10.203	10.129	10.125	0.04	10.152	11.635	11.605	11.572	0.03	11.604	5.391
**7**	4.654	4.614	4.612	0.02	4.627	4.560	4.545	4.523	0.02	4.543	2.810
**8**	7.541	7.472	7.445	0.05	7.486	7.845	7.802	7.754	0.05	7.800	4.490
**9**	5.010	4.961	4.945	0.03	4.972	4.944	4.921	4.886	0.03	4.917	2.880
**10**	7.833	7.772	7.750	0.04	7.785	8.240	8.193	8.150	0.05	8.194	4.193
**11**	7.042	6.986	6.967	0.04	6.998	7.181	7.150	7.104	0.04	7.145	3.824
**12**	7.201	7.139	7.126	0.04	7.155	7.385	7.357	7.310	0.04	7.351	3.897
**13**	6.359	6.301	6.283	0.04	6.314	6.347	6.309	6.286	0.03	6.314	3.532
**14**	7.232	7.182	7.168	0.03	7.194	7.425	7.390	7.340	0.04	7.385	4.310
**15**	9.338	9.273	9.252	0.04	9.288	10.744	10.706	10.657	0.04	10.702	5.074
**16**	8.352	8.301	8.293	0.03	8.315	9.042	9.003	8.957	0.04	9.001	4.476
**17**	8.970	8.931	8.916	0.03	8.939	10.135	10.093	10.046	0.04	10.091	4.540
**18**	7.894	7.841	7.824	0.04	7.853	8.329	8.295	8.254	0.04	8.293	4.228
**19**	7.345	7.296	7.274	0.04	7.305	7.595	7.562	7.498	0.05	7.552	3.967
**20**	6.959	6.920	6.896	0.03	6.925	7.085	7.043	7.005	0.04	7.044	3.789
**21**	8.654	8.617	8.588	0.03	8.620	9.590	9.552	9.512	0.04	9.551	4.480
**22**	7.915	7.881	7.853	0.03	7.883	8.401	8.375	8.329	0.04	8.368	4.254
**23**	8.230	8.199	8.185	0.02	8.205	8.773	8.732	8.852	0.06	8.786	4.401
**24**	7.563	7.540	7.517	0.02	7.540	7.932	7.882	7.877	0.03	7.897	4.088
**25**	7.633	7.609	7.581	0.03	7.608	6.065	7.926	7.972	1.09	7.321	3.886
**26**	4.959	4.933	4.911	0.02	4.934	4.854	4.844	4.848	0.01	4.849	2.870
**27**	8.210	8.166	8.140	0.04	8.172	8.758	8.709	8.827	0.06	8.765	4.460

Table S7. List of molecular descriptors used to build QSRR and QSAR models.

Descriptor	Full name	Block
JGI6	mean topological charge index of order 6	2D autocorrelations
SIC1	Structural Information Content index (neighborhood symmetry of 1-order)	Information indices
VE2sign_X	average coefficient of the last eigenvector from chi matrix	2D matrix-based descriptors
SHED_LL	SHED Lipophilic-Lipophilic	Pharmacophore descriptors
Mor32p	signal 32 / weighted by polarizability	3D-MoRSE descriptors
Mor14i	signal 14 / weighted by ionization potential	3D-MoRSE descriptors
VE2sign_X	average coefficient of the last eigenvector from chi matrix	2D matrix-based descriptors
VE1sign_D/Dt	coefficient sum of the last eigenvector from distance/detour matrix	2D matrix-based descriptors
L3s	3rd component size directional WHIM index / weighted by I-state	WHIM descriptors
SIC1	Structural Information Content index (neighborhood symmetry of 1-order)	Information indices
Mor19m	signal 19 / weighted by mass	3D-MoRSE descriptors
SpMin2_Bh(p)	smallest eigenvalue n. 2 of Burden matrix weighted by polarizability	Burden eigenvalues
R6p+	R maximal autocorrelation of lag 6 / weighted by polarizability	GETAWAY descriptors
MATS4e	Moran autocorrelation of lag 4 weighted by Sanderson electronegativity	2D autocorrelations
SpMin2_Bh(m)	smallest eigenvalue n. 2 of Burden matrix weighted by mass	Burden eigenvalues
B02[N-N]	Presence/absence of N – N at topological distance 2	2D Atom Pairs
RTs+	R maximal index / weighted by I-state	GETAWAY descriptors
HOMT	HOMA total	Geometrical descriptors
Gu	total symmetry index / unweighted	WHIM descriptors
H-049	H attached to C3(sp3)/C2(sp2)/C3(sp2)/C3(sp)	Atom-centred fragments
Mor08u	signal 08 / unweighted	3D-MoRSE descriptors
R3u	R autocorrelation of lag 3 / unweighted	GETAWAY descriptors
R5p+	R maximal autocorrelation of lag 5 / weighted by polarizability	GETAWAY descriptors
Mor25u	signal 25 / unweighted	3D-MoRSE descriptors
G2p	2nd component symmetry directional WHIM index / weighted by polarizability	WHIM descriptors
WHALES00_IR	WHALES Isolation-Remoteness ratio (IR)	WHALES descriptors
TDB05r	3D Topological distance based descriptors – lag 5 weighted by covalent radius	3D autocorrelations
RPCG	relative positive charge	Charge descriptors

Table S8. The values of alvaDesc Molecular Descriptors for target compound

No.	JGI6	SIC1	VE2sign_X	SHED_LL	Mor32p	Mor14i	VE2sign_X	VE1sign_D/Dt	L3s	SIC1	Mor19m	SpMin2_Bh(p)	R6p+	MATS4e	SpMin2_Bh(m)	B02[N-N]	RTs+	HOMT	Gu	H-049	Mor08u	R3u	R5p+	Mor25u
1	0.020	0.658	0.018	7.823	-0.492	-0.402	0.018	0.081	1.288	0.658	-0.174	1.958	0.017	0.068	1.902	1.000	3.344	15.838	0.178	1.000	0.543	1.609	0.023	1.374
2	0.021	0.543	0.009	11.877	-0.524	-0.452	0.009	0.253	1.667	0.543	0.127	2.031	0.018	-0.198	1.996	0.000	2.974	20.364	0.193	1.000	-0.478	1.772	0.022	1.788
3	0.022	0.605	0.004	11.635	-0.498	0.020	0.004	0.255	1.848	0.605	-0.057	2.032	0.020	-0.186	1.996	0.000	2.087	20.311	0.176	1.000	0.055	1.752	0.022	1.761
4	0.023	0.585	0.003	12.176	-0.508	-0.541	0.003	0.256	1.899	0.585	0.248	2.033	0.024	-0.168	1.996	0.000	2.351	20.224	0.190	0.000	-1.115	1.754	0.020	2.118
5	0.021	0.589	0.012	10.127	-0.354	-0.448	0.012	0.214	1.544	0.589	-0.102	1.976	0.017	-0.218	1.970	1.000	3.171	17.334	0.177	0.000	-0.196	1.675	0.045	1.356
6	0.024	0.542	0.005	10.873	-0.468	-0.219	0.005	0.105	1.353	0.542	0.244	1.951	0.036	-0.234	1.957	0.000	3.091	15.835	0.187	0.000	0.390	1.750	0.026	1.394
7	0.021	0.643	0.023	7.584	-0.510	-0.774	0.023	0.077	1.343	0.643	-0.115	1.976	0.018	0.119	1.938	1.000	3.068	15.829	0.190	0.000	0.042	1.590	0.022	1.361
8	0.020	0.675	0.002	15.237	-0.525	-0.989	0.002	0.041	3.375	0.675	0.305	2.009	0.009	-0.059	2.020	1.000	2.787	18.154	0.175	0.000	0.176	1.677	0.020	1.645
9	0.019	0.678	0.005	11.969	-0.391	-0.799	0.005	0.072	3.448	0.678	-0.077	2.023	0.019	0.072	1.954	1.000	1.953	19.771	0.174	0.000	1.093	1.647	0.024	1.404
10	0.018	0.623	0.006	16.200	-0.368	-0.518	0.006	0.160	3.593	0.623	0.161	2.043	0.012	0.168	2.024	1.000	1.622	22.431	0.167	0.000	-0.152	1.820	0.022	1.773
11	0.018	0.623	0.006	16.200	-0.427	-0.141	0.006	0.160	2.907	0.623	-0.058	2.043	0.012	0.168	2.024	1.000	1.738	22.446	0.166	0.000	-0.343	1.817	0.022	1.613
12	0.019	0.726	0.002	15.455	-0.440	-0.193	0.002	0.156	2.677	0.726	-0.112	2.020	0.013	-0.069	1.960	1.000	2.623	19.666	0.172	0.000	1.546	1.610	0.020	1.748
13	0.018	0.659	0.003	14.266	-0.542	-0.099	0.003	0.125	2.836	0.659	-0.175	1.998	0.016	0.053	2.007	1.000	2.035	20.458	0.177	1.000	-0.051	1.682	0.023	1.297
14	0.016	0.616	0.007	14.630	-0.551	0.409	0.007	0.147	2.976	0.616	0.555	2.017	0.014	0.126	2.015	1.000	1.622	23.483	0.170	1.000	-0.388	1.755	0.016	1.424
15	0.019	0.634	0.002	15.693	-0.656	-0.588	0.002	0.172	2.057	0.634	0.232	2.018	0.017	-0.060	2.024	1.000	2.701	23.363	0.163	1.000	0.139	1.750	0.017	1.617
16	0.017	0.633	0.005	15.251	-0.552	0.252	0.005	0.177	2.116	0.633	0.440	2.013	0.017	0.076	2.013	1.000	1.716	23.368	0.174	1.000	0.203	1.687	0.017	1.748
17	0.019	0.633	0.002	15.772	-0.393	-0.562	0.002	0.172	2.954	0.633	0.267	2.028	0.016	-0.008	2.028	1.000	2.256	23.531	0.164	1.000	0.329	1.819	0.019	1.715
18	0.017	0.628	0.005	15.339	-0.485	-0.145	0.005	0.177	3.261	0.628	0.201	2.024	0.015	0.142	2.028	1.000	2.119	23.545	0.162	1.000	0.652	1.747	0.019	1.603
19	0.017	0.629	0.004	14.669	-0.403	-0.116	0.004	0.175	2.887	0.629	-0.172	2.027	0.014	0.131	2.028	1.000	1.856	22.960	0.176	1.000	-0.551	1.593	0.016	1.836
20	0.017	0.626	0.006	14.211	-0.582	0.481	0.006	0.205	2.102	0.626	-0.395	2.019	0.013	0.076	2.013	1.000	1.529	23.577	0.171	1.000	1.234	1.664	0.016	2.136
21	0.021	0.662	0.003	14.245	-0.677	-1.836	0.003	0.093	2.953	0.662	-0.290	2.013	0.013	-0.157	2.020	1.000	2.528	20.375	0.177	1.000	0.307	1.608	0.021	1.431
22	0.018	0.636	0.002	15.107	-0.697	-0.086	0.002	0.107	2.506	0.636	-0.053	2.052	0.012	0.064	2.007	1.000	0.924	24.734	0.178	1.000	0.521	1.696	0.017	1.846
23	0.018	0.631	0.004	14.591	-0.739	0.045	0.004	0.168	2.464	0.631	0.276	2.053	0.014	0.080	2.013	1.000	1.302	24.549	0.166	1.000	0.877	1.596	0.015	2.122
24	0.019	0.690	0.002	14.266	-0.654	-1.500	0.002	0.008	2.773	0.690	-0.285	2.010	0.014	-0.101	2.007	1.000	2.588	20.399	0.176	1.000	0.190	1.639	0.023	1.378
25	0.019	0.680	0.001	13.743	-0.646	-1.086	0.001	0.038	2.955	0.680	-0.282	2.010	0.013	-0.090	2.013	1.000	2.523	20.354	0.185	1.000	0.665	1.538	0.020	1.651
26	0.020	0.640	0.010	10.589	-0.415	0.235	0.010	0.324	2.284	0.640	0.534	2.010	0.014	0.147	1.954	0.000	1.452	15.544	0.161	0.000	0.663	1.548	0.020	1.653
27	0.018	0.579	0.005	15.251	-0.250	1.362	0.005	0.238	4.250	0.579	0.199	2.021	0.013	0.151	2.039	0.000	1.113	18.757	0.174	0.000	0.154	1.814	0.022	1.778

Figure S1. William’s plots and y-randomization tests (Q^2 vs. R^2) generated for each model. The numbers correspond to the next stationary phases A) IAM. B) C_{18}. C) C_8. D) CN . E) Ph and F) pIC_{50}

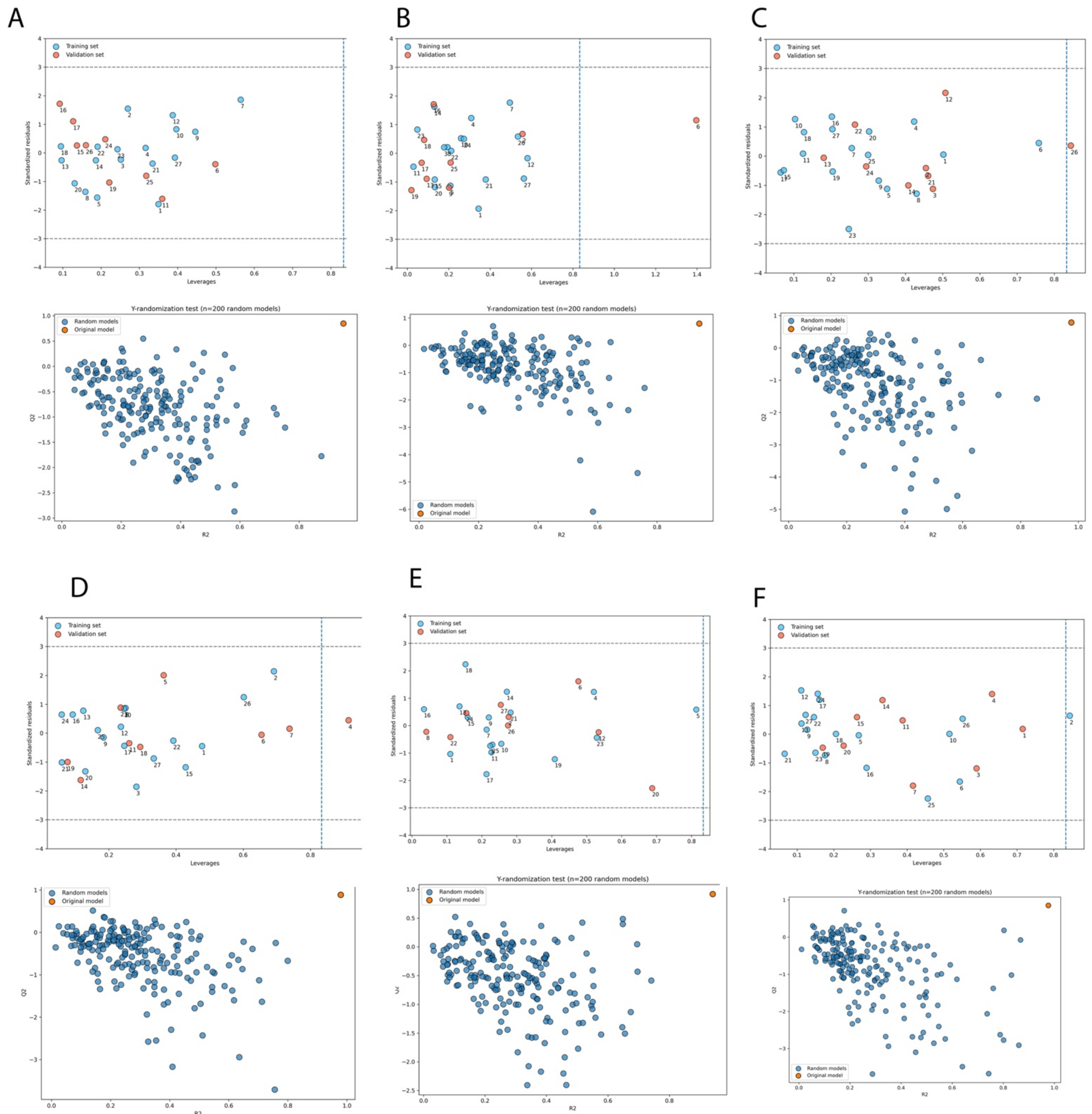

Spectrum S1 ^{1}H NMR of compound **1** (500 MHz. DMSO-d_6).
Spectrum S2 ^{13}C NMR of compound **1** (125 MHz. DMSO-d_6).
Spectrum S3 ^{1}H NMR of compound **2** (500 MHz. DMSO-d_6).
Spectrum S4 ^{13}C NMR of compound **2** (125 MHz. DMSO-d_6).
Spectrum S5 ^{1}H NMR of compound **3** (500 MHz. DMSO-d_6).
Spectrum S6 ^{13}C NMR of compound **3** (125 MHz. DMSO-d_6).
Spectrum S7 ^{1}H NMR of compound **4** (500 MHz. DMSO-d_6).
Spectrum S8 ^{1}H NMR of compound **5** (200 MHz. DMSO-d_6).
Spectrum S9 ^{13}C NMR of compound **5** (50 MHz. DMSO-d_6).
Spectrum S10 ^{1}H NMR of compound **6** (500 MHz. DMSO-d_6).
Spectrum S11 ^{13}C NMR of compound **6** (125 MHz. DMSO-d_6).
Spectrum S12 ^{1}H NMR of compound **7** (500 MHz. DMSO-d_6).
Spectrum S13 ^{13}C NMR of compound **7** (125 MHz. DMSO-d_6).
Spectrum S14 ^{1}H NMR of compound **8** (500 MHz. DMSO-d_6).
Spectrum S15 ^{13}C NMR of compound **8** (125 MHz. DMSO-d_6).
Spectrum S16 ^{1}H NMR of compound **9** (500 MHz. DMSO-d_6).
Spectrum S17 ^{13}C NMR of compound **9** (125 MHz. DMSO-d_6).
Spectrum S18 ^{1}H NMR of compound **10** (500 MHz. DMSO-d_6).
Spectrum S19 ^{13}C NMR of compound **10** (125 MHz. DMSO-d_6).
Spectrum S20 ^{1}H NMR of compound **11** (200 MHz. DMSO-d_6).
Spectrum S21 ^{1}H NMR of compound **12** (200 MHz. DMSO-d_6).
Spectrum S22 ^{1}H NMR of compound **13** (200 MHz. DMSO-d_6).
Spectrum S23 ^{13}C NMR of compound **13** (50 MHz. DMSO-d_6).
Spectrum S24 ^{1}H NMR of compound **14** (200 MHz. DMSO-d_6).
Spectrum S25 ^{1}H NMR of compound **15** (200 MHz. DMSO-d_6).
Spectrum S26 ^{13}C NMR of compound **15** (50 MHz. DMSO-d_6).
Spectrum S27 ^{1}H NMR of compound **16** (200 MHz. DMSO-d_6).
Spectrum S28 ^{1}H NMR of compound **17** (200 MHz. DMSO-d_6).
Spectrum S29 ^{13}C NMR of compound **17** (50 MHz. DMSO-d_6).
Spectrum S30 ^{1}H NMR of compound **18** (200 MHz. DMSO-d_6).
Spectrum S31 ^{13}C NMR of compound **18** (50 MHz. DMSO-d_6).
Spectrum S32 ^{1}H NMR of compound **19** (200 MHz. DMSO-d_6).
Spectrum S33 ^{13}C NMR of compound **19** (50 MHz. DMSO-d_6).
Spectrum S34 ^{1}H NMR of compound **20** (200 MHz. DMSO-d_6).
Spectrum S35 ^{13}C NMR of compound **20** (50 MHz. DMSO-d_6).
Spectrum S36 ^{1}H NMR of compound **21** (500 MHz. DMSO-d_6).
Spectrum S37 ^{1}H NMR of compound **22** (500 MHz. DMSO-d_6).
Spectrum S38 ^{1}H NMR of compound **23** (500 MHz. DMSO-d_6).
Spectrum S39 ^{13}C NMR of compound **23** (125 MHz. DMSO-d_6).
Spectrum S40 ^{1}H NMR of compound **24** (500 MHz. DMSO-d_6).
Spectrum S41 ^{1}H NMR of compound **25** (500 MHz. DMSO-d_6).
Spectrum S42 ^{13}C NMR of compound **25** (125 MHz. DMSO-d_6).
Spectrum S43 ^{1}H NMR of compound **26** (200 MHz. DMSO-d_6).
Spectrum S44 ^{1}H NMR of compound **27** (200 MHz. DMSO-d_6).
Spectrum S45 ^{13}C NMR of compound **27** (50 MHz. DMSO-d_6).

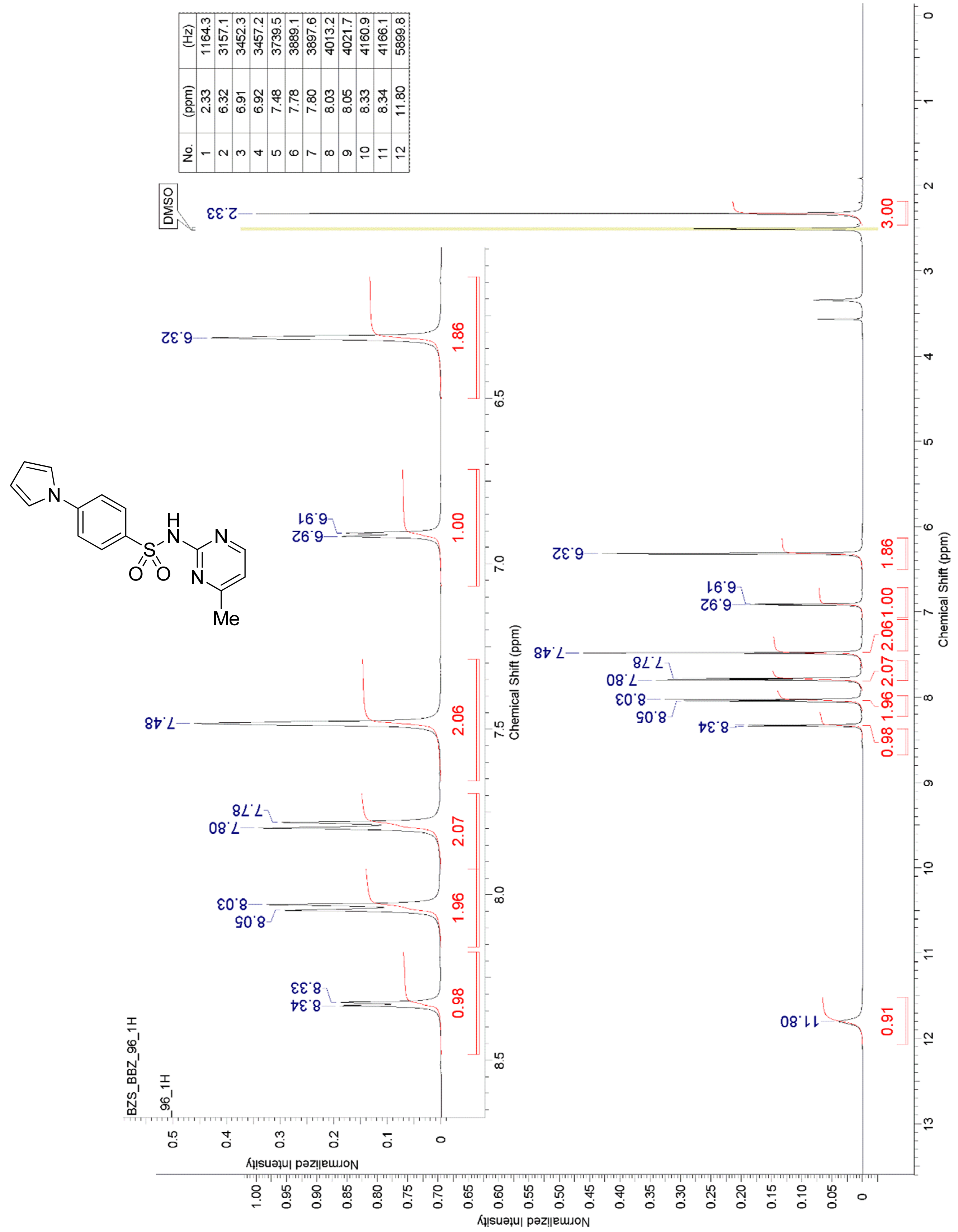

Spectrum S1. ^{1}H NMR of compd **1** (500 MHz. DMSO-d_6).

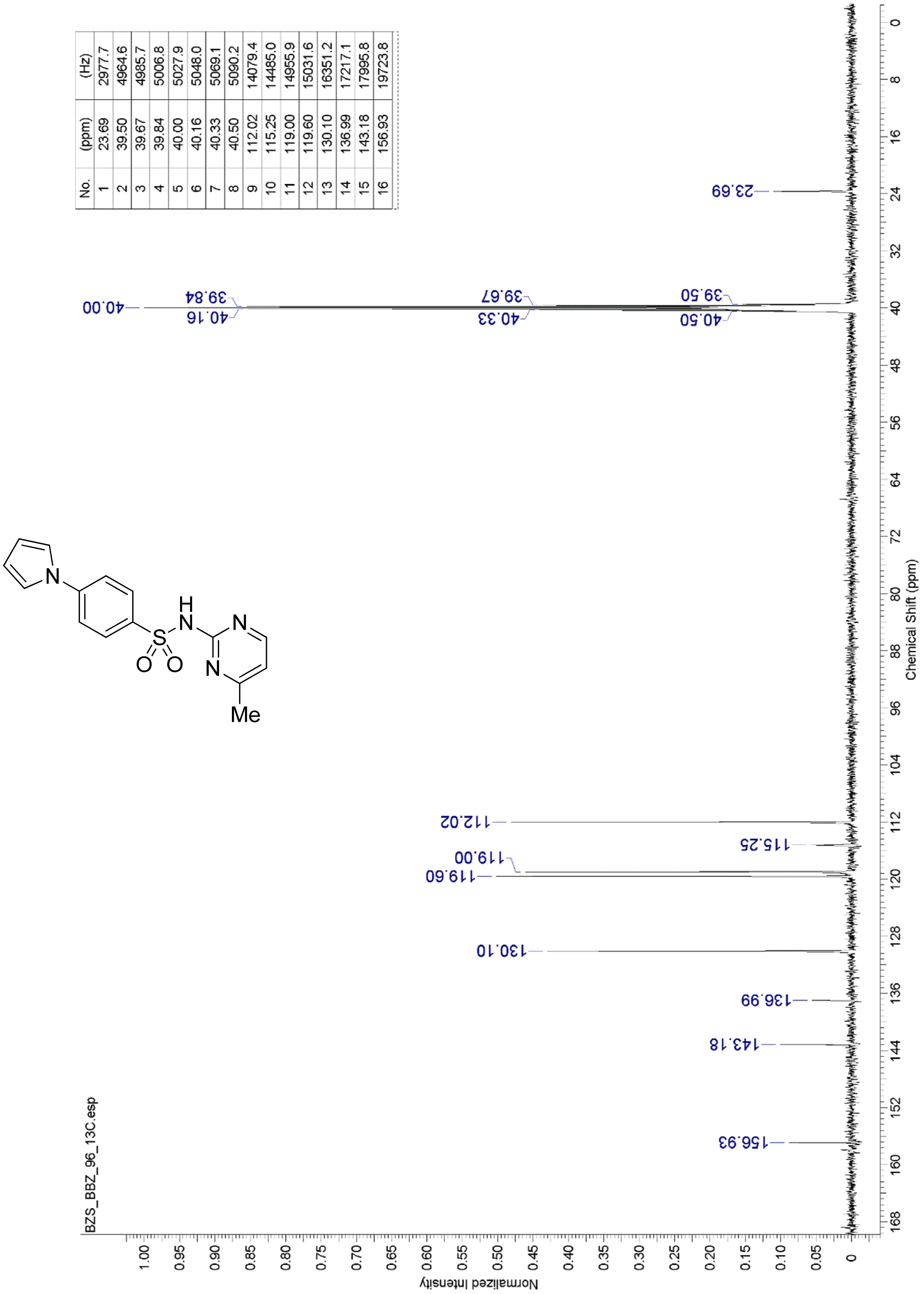

No.	(ppm)	(Hz)
1	23.69	2977.7
2	39.50	4964.6
3	39.67	4985.7
4	39.84	5006.8
5	40.00	5027.9
6	40.16	5048.0
7	40.33	5069.1
8	40.50	5090.2
9	112.02	14079.4
10	115.25	14485.0
11	119.00	14955.9
12	119.60	15031.6
13	130.10	16351.2
14	136.99	17217.1
15	143.18	17995.8
16	156.93	19723.8

Spectrum S2. ^{13}C NMR of compd **1** (125 MHz. DMSO-d_6).

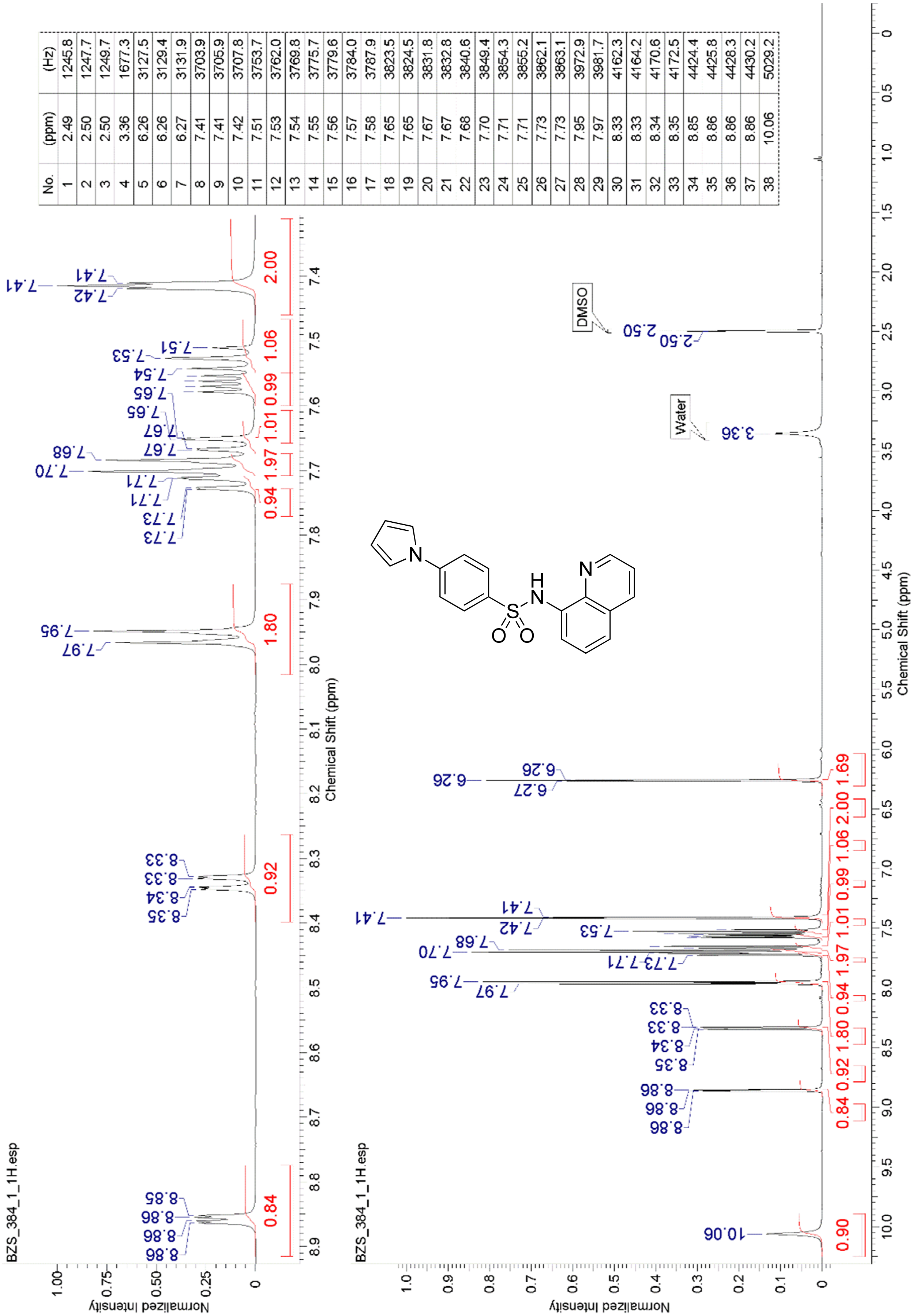

No.	(ppm)	(Hz)
1	2.49	1245.8
2	2.50	1247.7
3	2.50	1249.7
4	3.36	1677.3
5	6.26	3127.5
6	6.26	3129.4
7	6.27	3131.9
8	7.41	3703.9
9	7.41	3705.9
10	7.42	3707.8
11	7.51	3753.7
12	7.53	3762.0
13	7.54	3769.8
14	7.55	3775.7
15	7.56	3779.6
16	7.57	3784.0
17	7.58	3787.9
18	7.65	3823.5
19	7.65	3824.5
20	7.67	3831.8
21	7.67	3832.8
22	7.68	3840.6
23	7.70	3849.4
24	7.71	3854.3
25	7.71	3855.2
26	7.73	3862.1
27	7.73	3863.1
28	7.95	3972.9
29	7.97	3981.7
30	8.33	4162.3
31	8.33	4164.2
32	8.34	4170.6
33	8.35	4172.5
34	8.85	4424.4
35	8.86	4425.8
36	8.86	4428.3
37	8.86	4430.2
38	10.06	5029.2

Spectrum S3. ^{1}H NMR of compd **2** (500 MHz. DMSO-d_6).

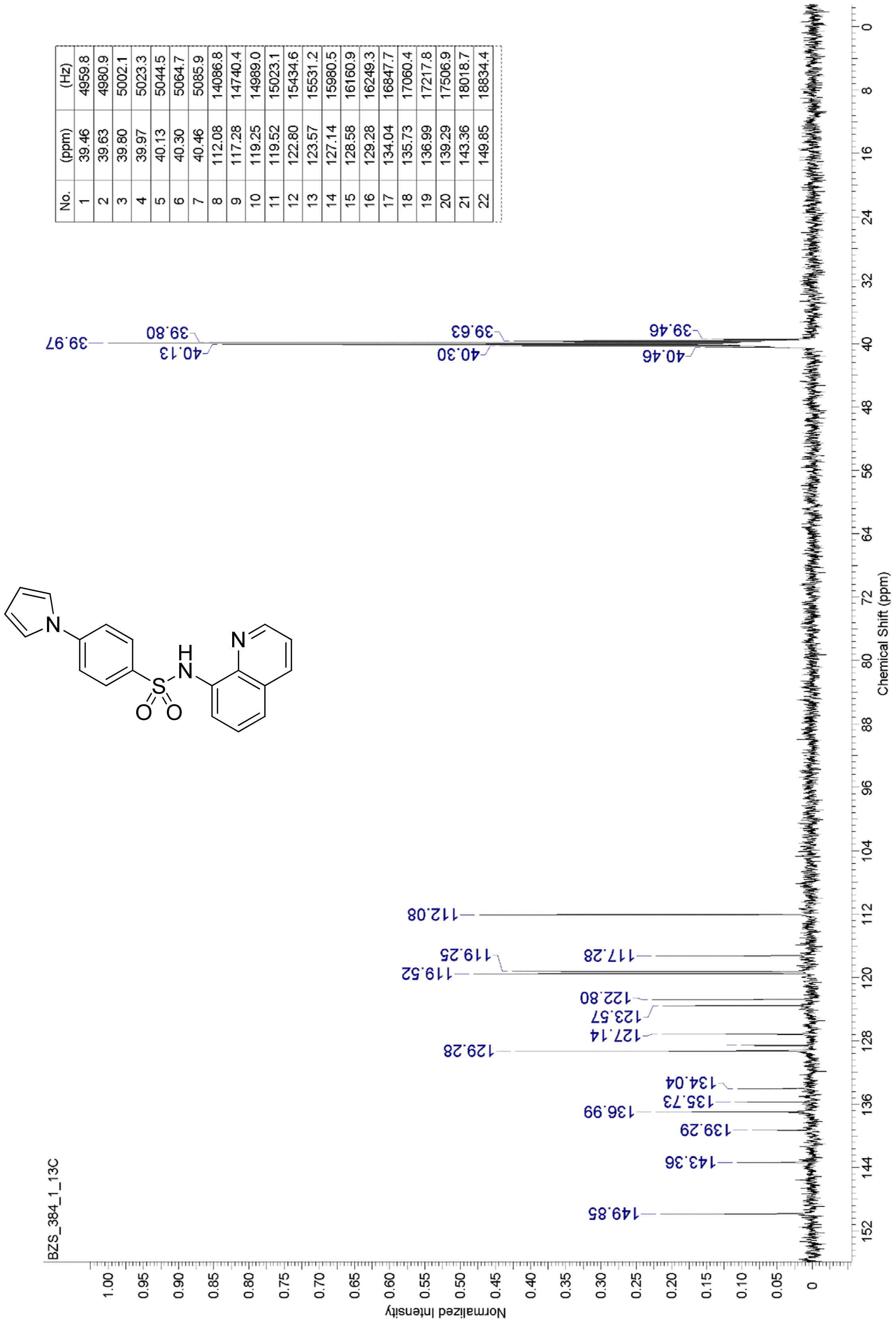

No.	(ppm)	(Hz)
1	39.46	4959.8
2	39.63	4980.9
3	39.80	5002.1
4	39.97	5023.3
5	40.13	5044.5
6	40.30	5064.7
7	40.46	5085.9
8	112.08	14086.8
9	117.28	14740.4
10	119.25	14989.0
11	119.52	15023.1
12	122.80	15434.6
13	123.57	15531.2
14	127.14	15980.5
15	128.58	16160.9
16	129.28	16249.3
17	134.04	16847.7
18	135.73	17060.4
19	136.99	17217.8
20	139.29	17506.9
21	143.36	18018.7
22	149.85	18834.4

Spectrum S4. ^{13}C NMR of compd **2** (125 MHz. DMSO-d_6).

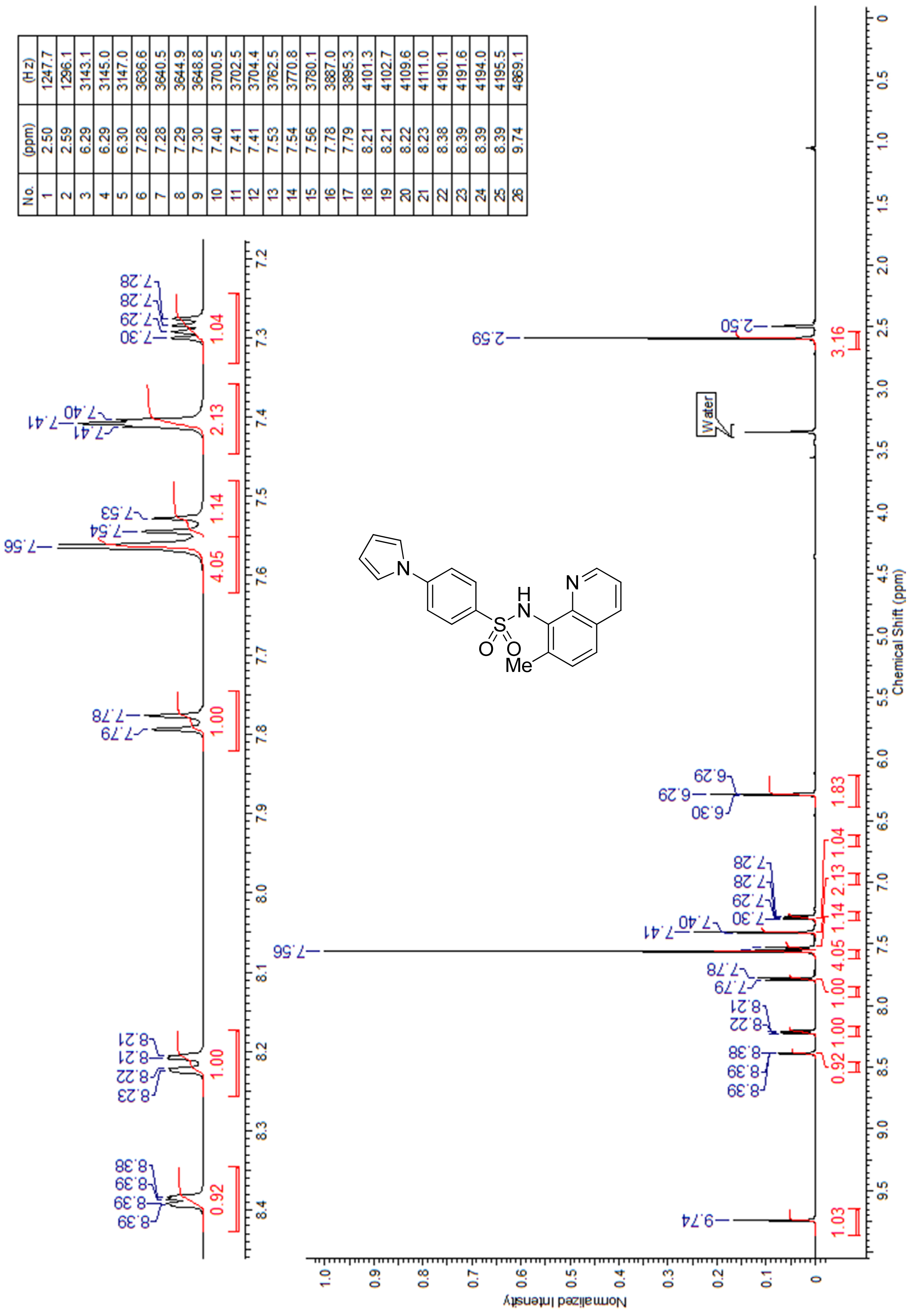

Spectrum S5. ^{1}H NMR of compd **3** (500 MHz. DMSO-d_6).

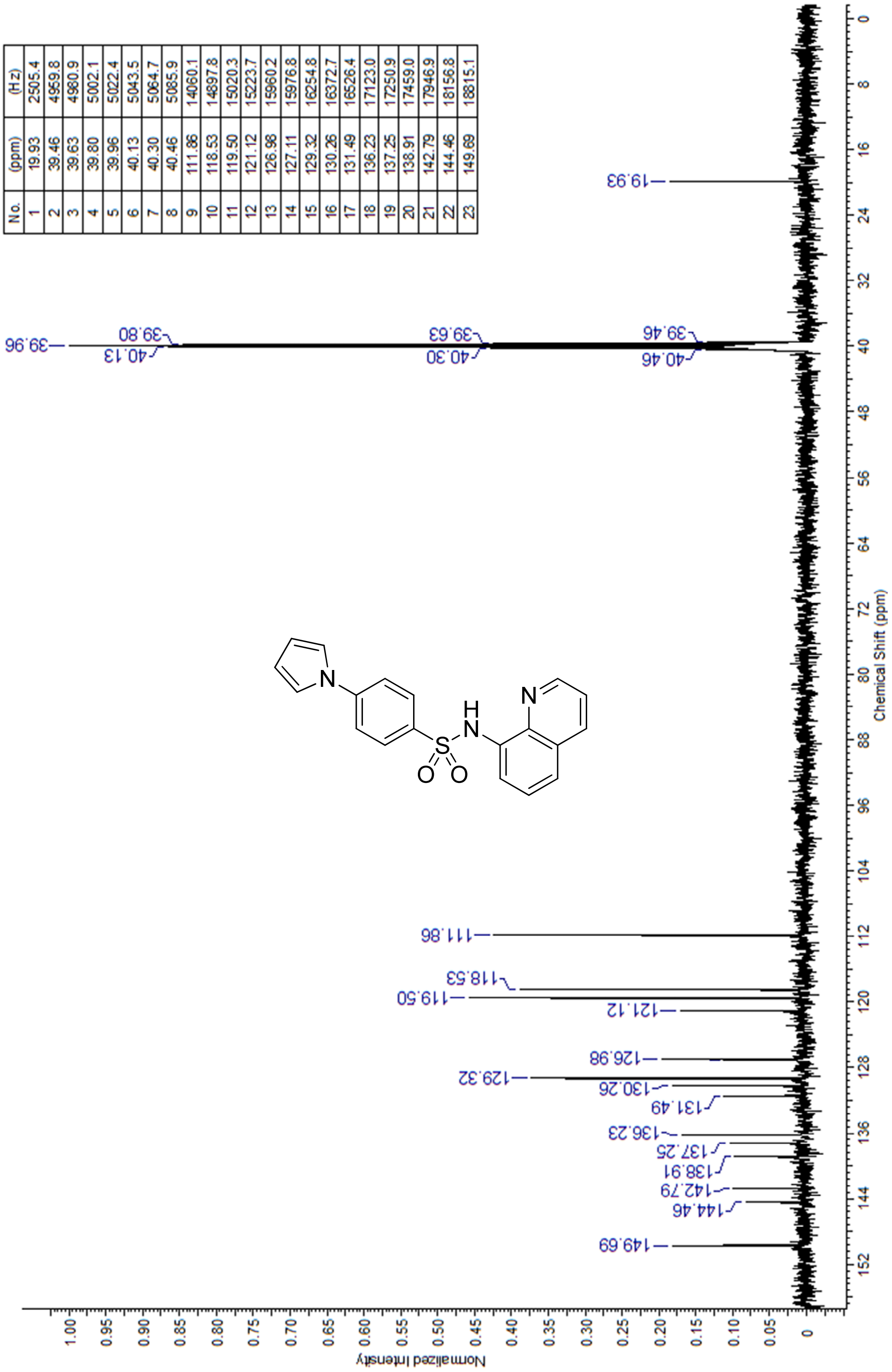

No.	(ppm)	(Hz)
1	19.93	2505.4
2	39.46	4959.8
3	39.63	4980.9
4	39.80	5002.1
5	39.96	5022.4
6	40.13	5043.5
7	40.30	5064.7
8	40.46	5085.9
9	111.86	14060.1
10	118.53	14897.8
11	119.50	15020.3
12	121.12	15223.7
13	126.98	15960.2
14	127.11	15976.8
15	129.32	16254.8
16	130.26	16372.7
17	131.49	16526.4
18	136.23	17123.0
19	137.25	17250.9
20	138.91	17459.0
21	142.79	17946.9
22	144.46	18156.8
23	149.69	18815.1

Spectrum S6. ^{13}C NMR of compd **3** (125 MHz. DMSO-d_6).

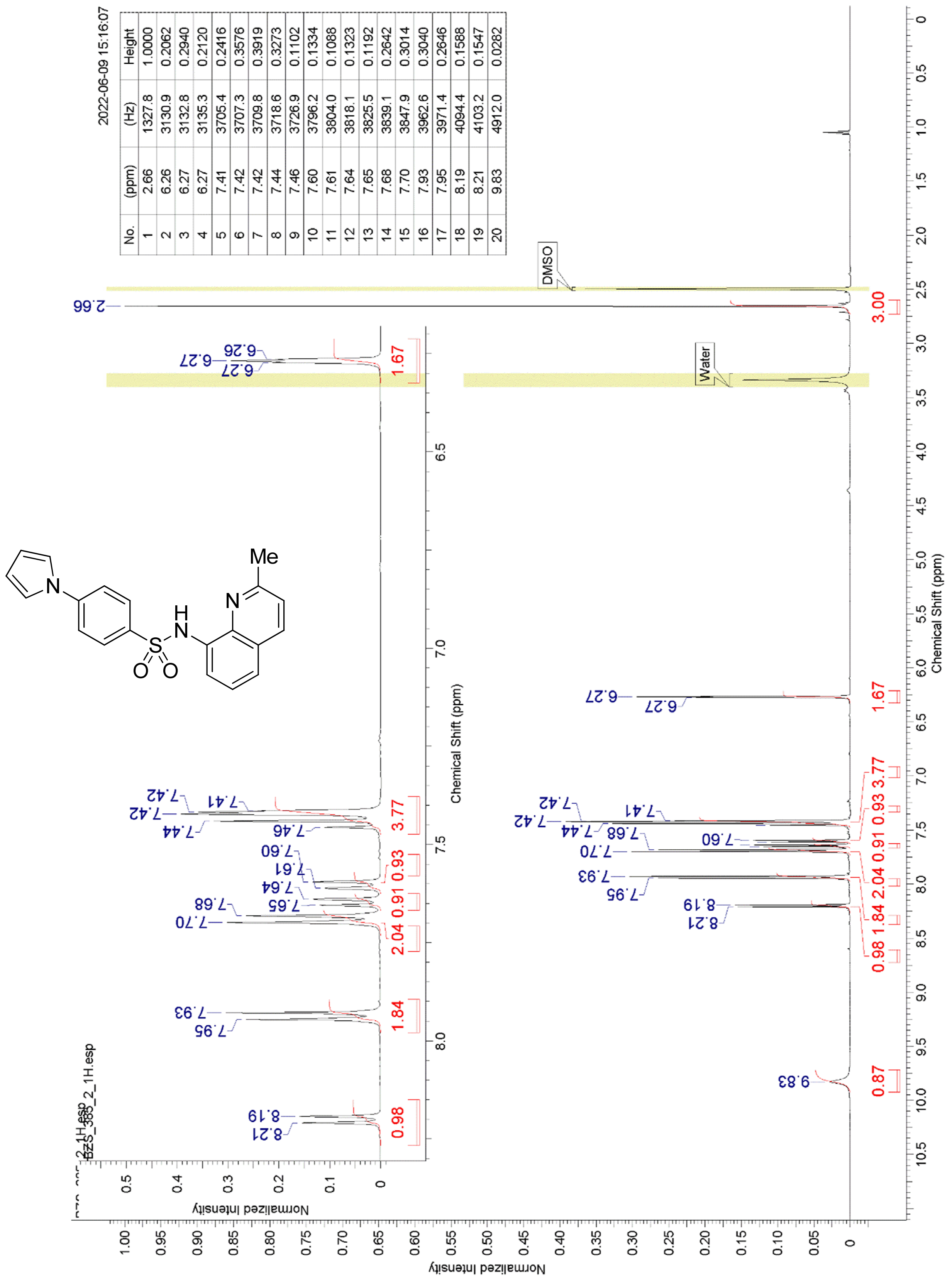

No.	(ppm)	(Hz)	Height
1	2.66	1327.8	1.0000
2	6.26	3130.9	0.2062
3	6.27	3132.8	0.2940
4	6.27	3135.3	0.2120
5	7.41	3705.4	0.2416
6	7.42	3707.3	0.3576
7	7.42	3709.8	0.3919
8	7.44	3718.6	0.3273
9	7.46	3726.9	0.1102
10	7.60	3796.2	0.1334
11	7.61	3804.0	0.1088
12	7.64	3818.1	0.1323
13	7.65	3825.5	0.1192
14	7.68	3839.1	0.2642
15	7.70	3847.9	0.3014
16	7.93	3962.6	0.3040
17	7.95	3971.4	0.2646
18	8.19	4094.4	0.1588
19	8.21	4103.2	0.1547
20	9.83	4912.0	0.0282

Spectrum S7. ^{1}H NMR of compd **4** (500 MHz. DMSO-d_6).

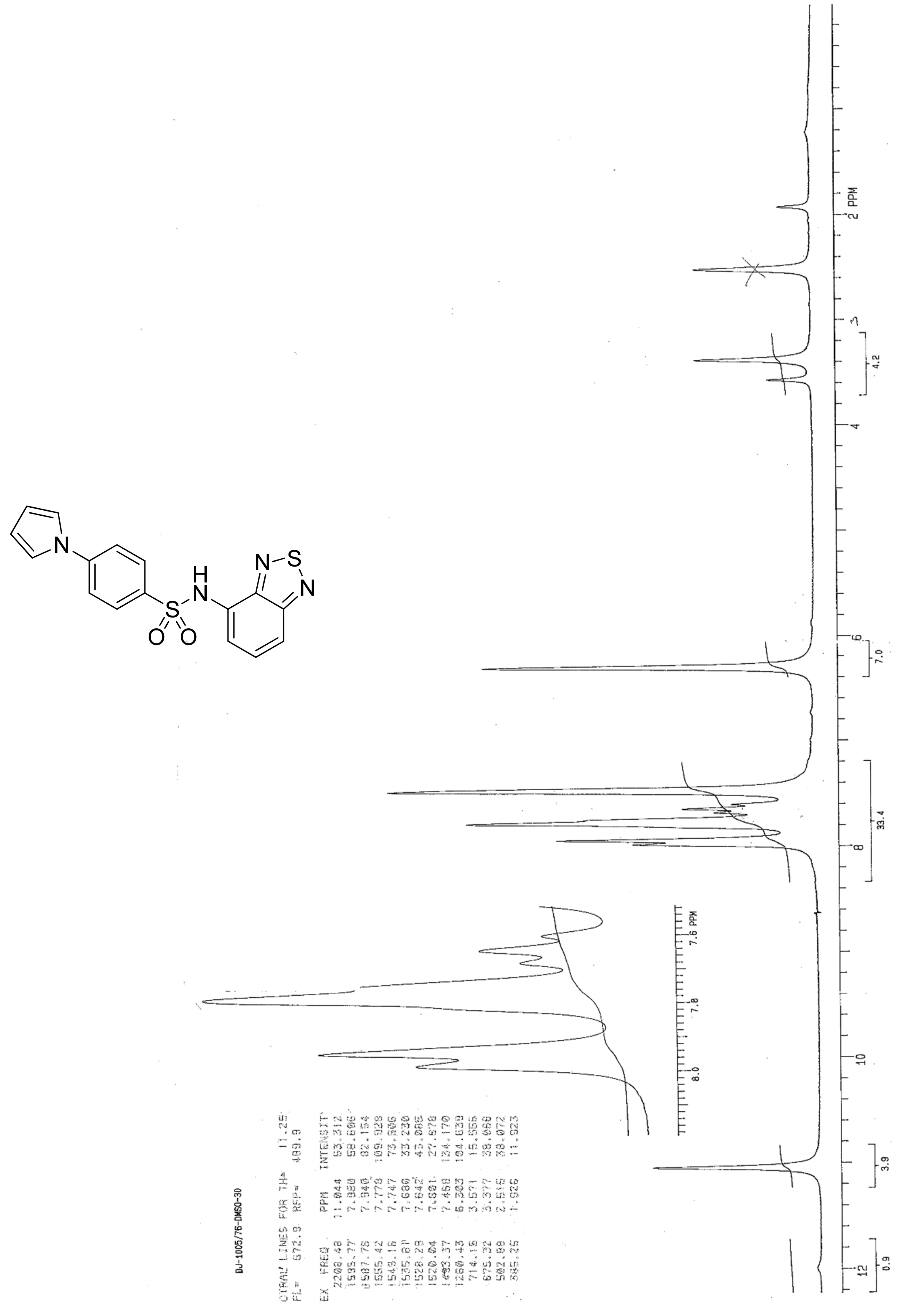

Spectrum S8. ^{1}H NMR of compd **5** (200 MHz. DMSO-d_6).

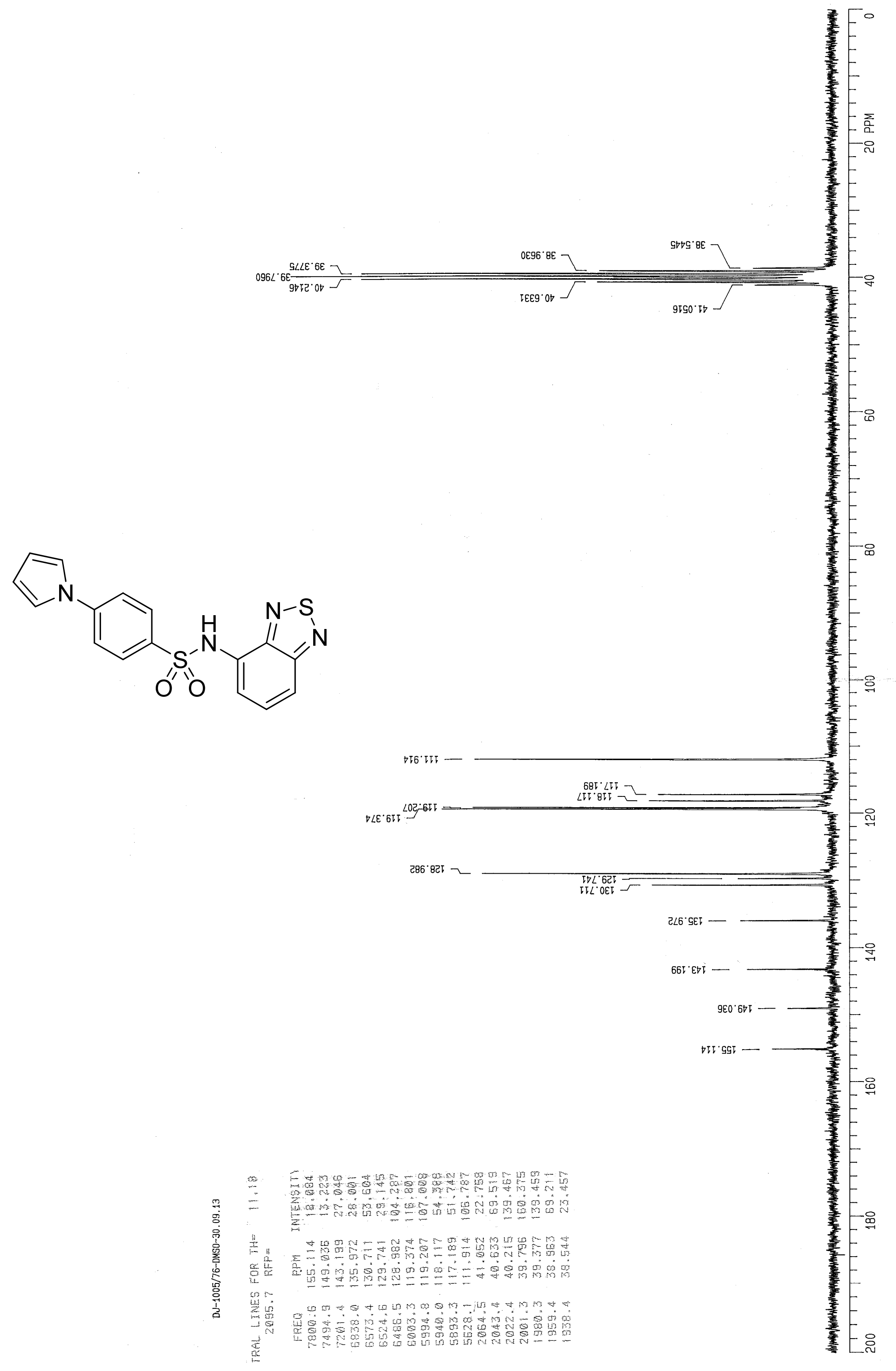

Spectrum S9. ^{13}C NMR of compd **5** (125 MHz. DMSO-d_6).

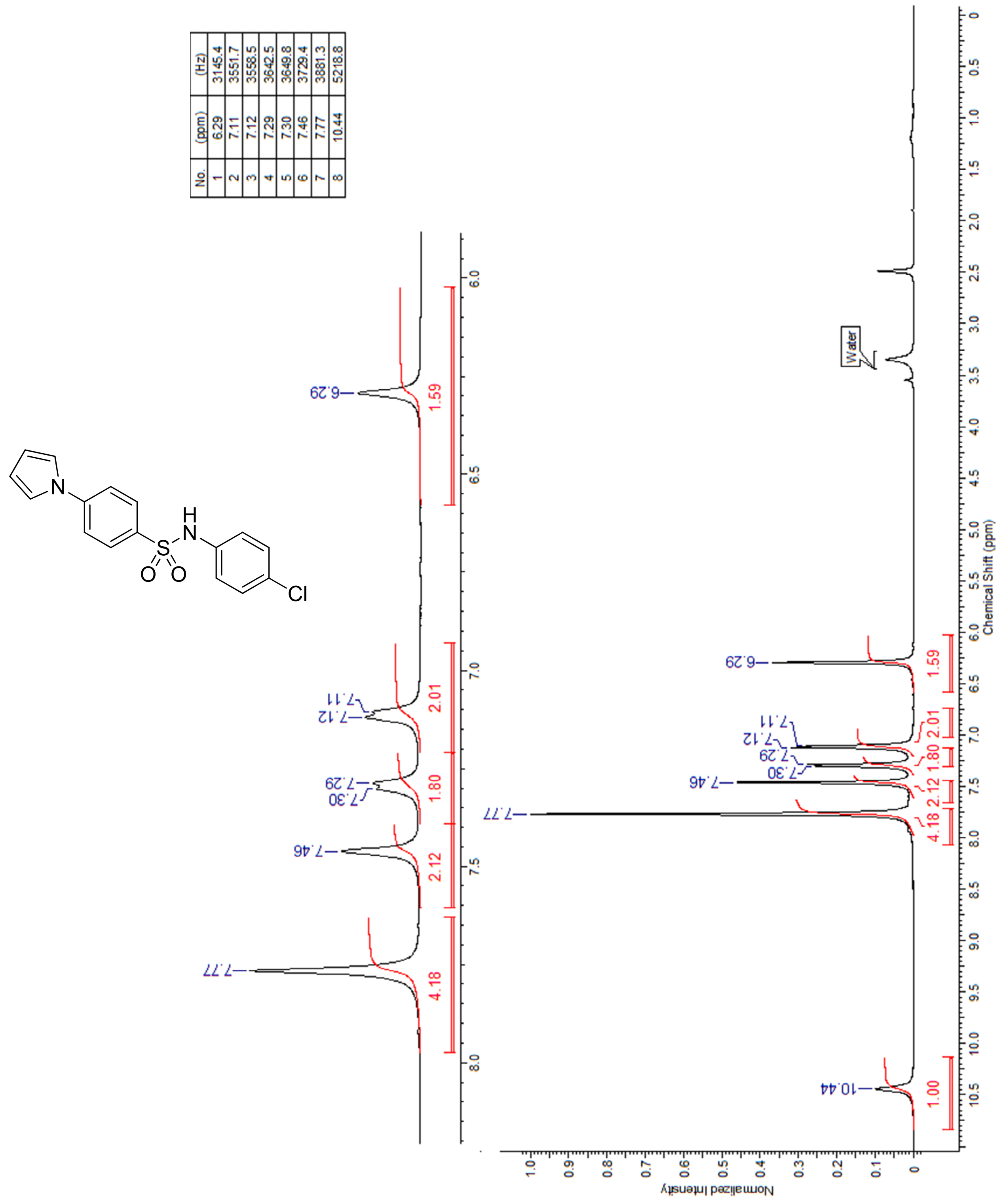

Spectrum S10. ^{1}H NMR of compd **6** (500 MHz. DMSO-d_6).

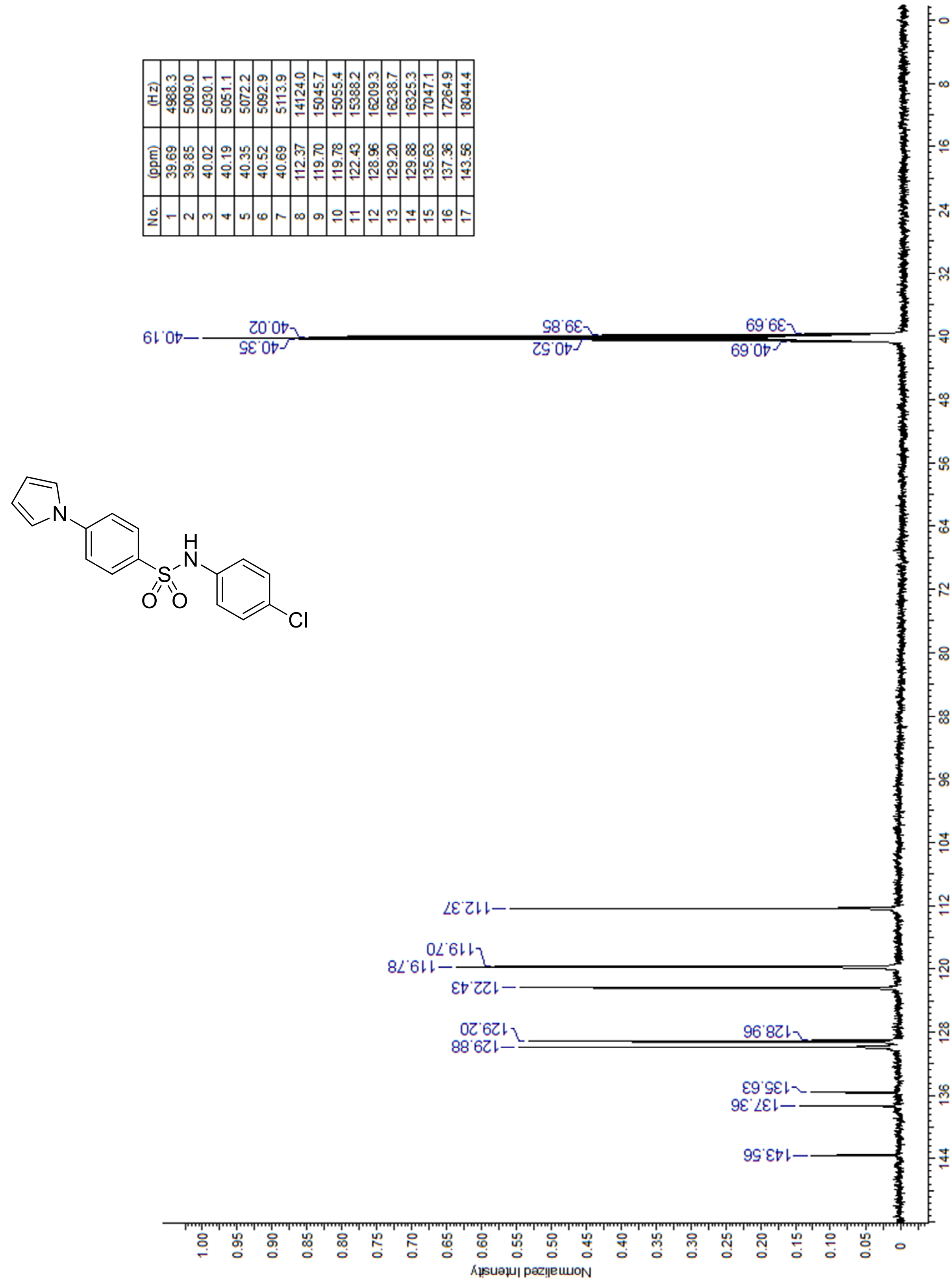

Spectrum S11. ^{13}C NMR of compd **6** (125 MHz. DMSO-d_6).

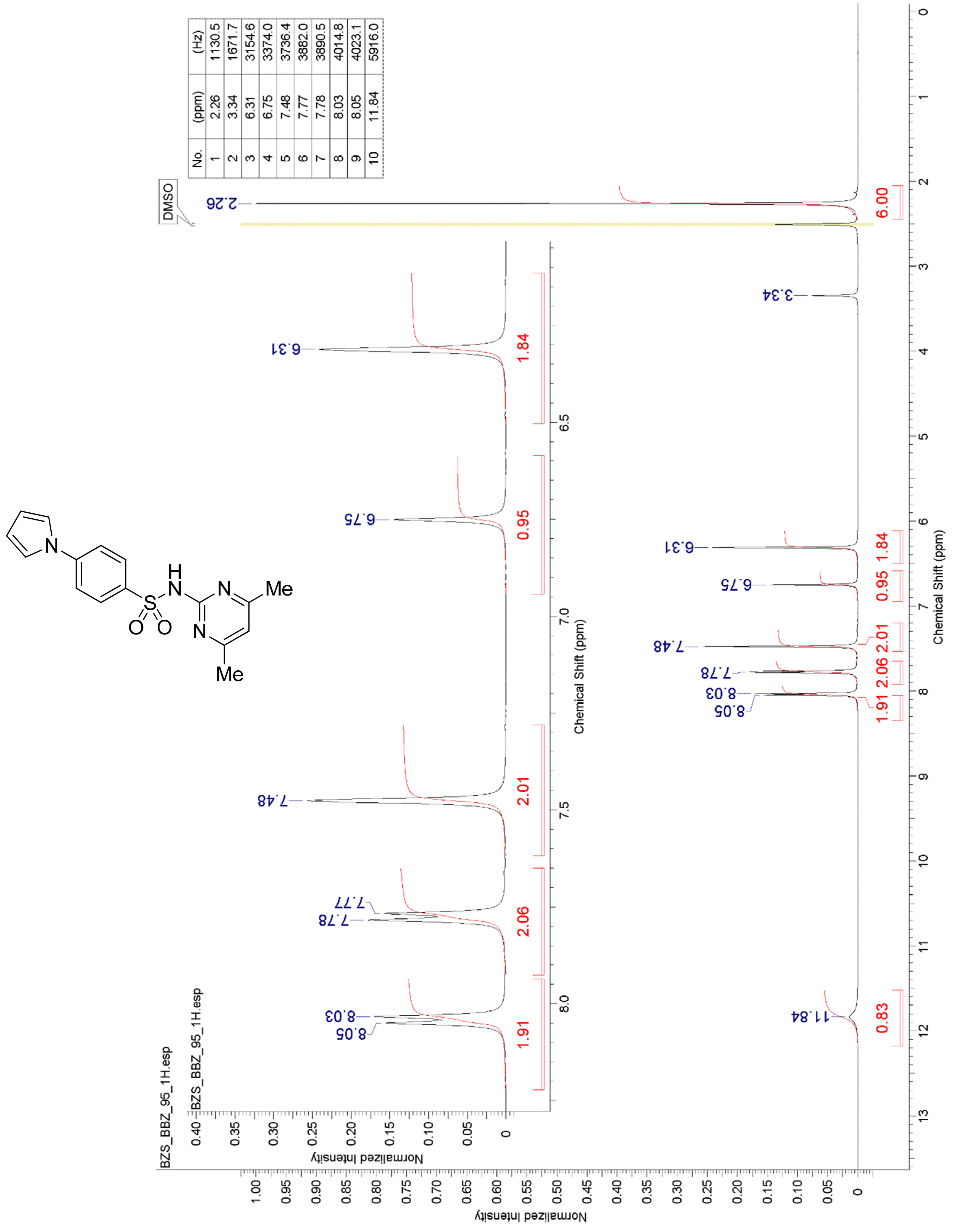

Spectrum S12. ^{1}H NMR of compd **7** (500 MHz. DMSO-d_6).

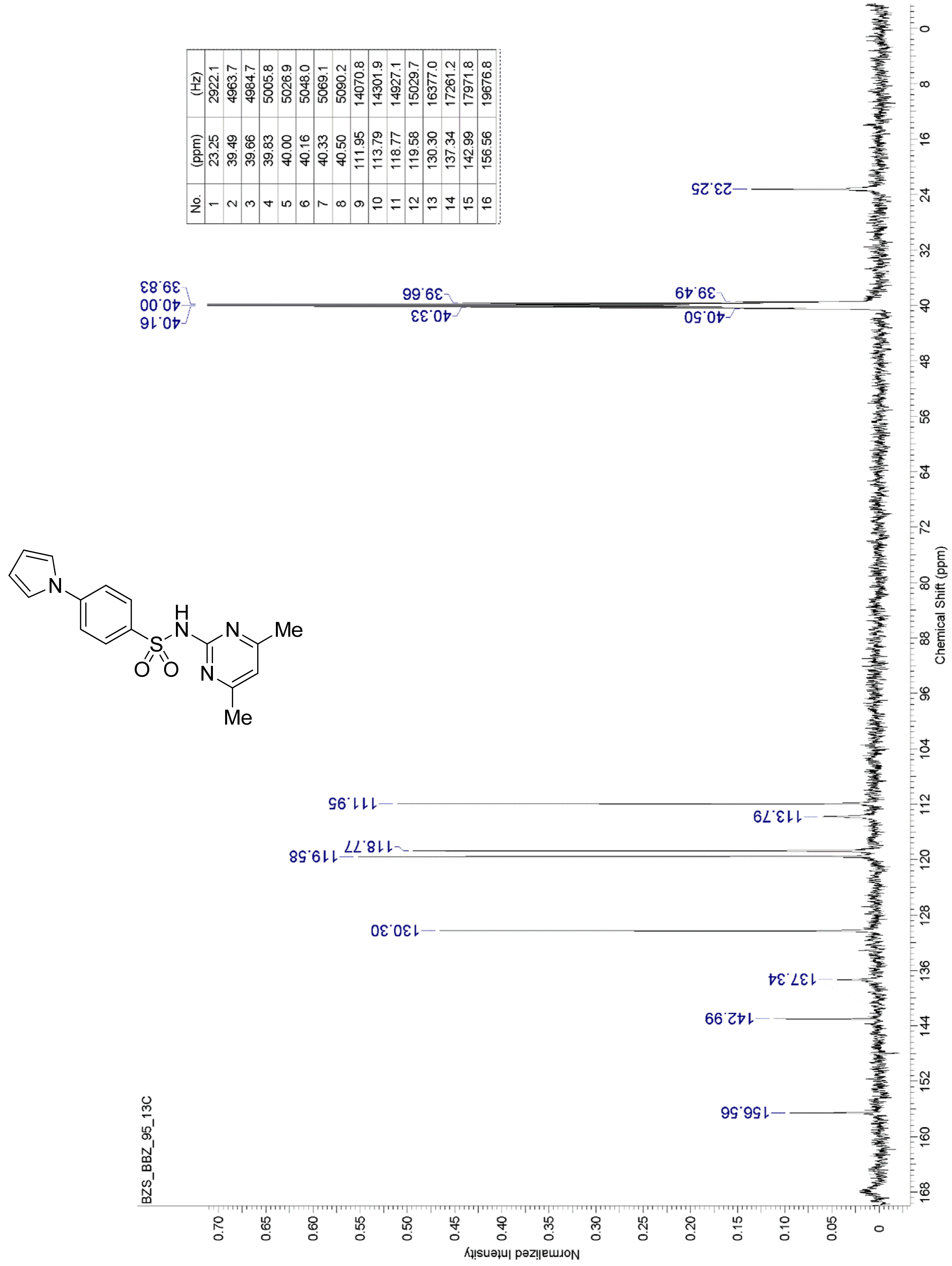

No.	(ppm)	(Hz)
1	23.25	2922.1
2	39.49	4963.7
3	39.66	4984.7
4	39.83	5005.8
5	40.00	5026.9
6	40.16	5048.0
7	40.33	5069.1
8	40.50	5090.2
9	111.95	14070.8
10	113.79	14301.9
11	118.77	14927.1
12	119.58	15029.7
13	130.30	16377.0
14	137.34	17261.2
15	142.99	17971.8
16	156.56	19676.8

Spectrum S13. ^{13}C NMR of compd **7** (125 MHz. DMSO-d_6).

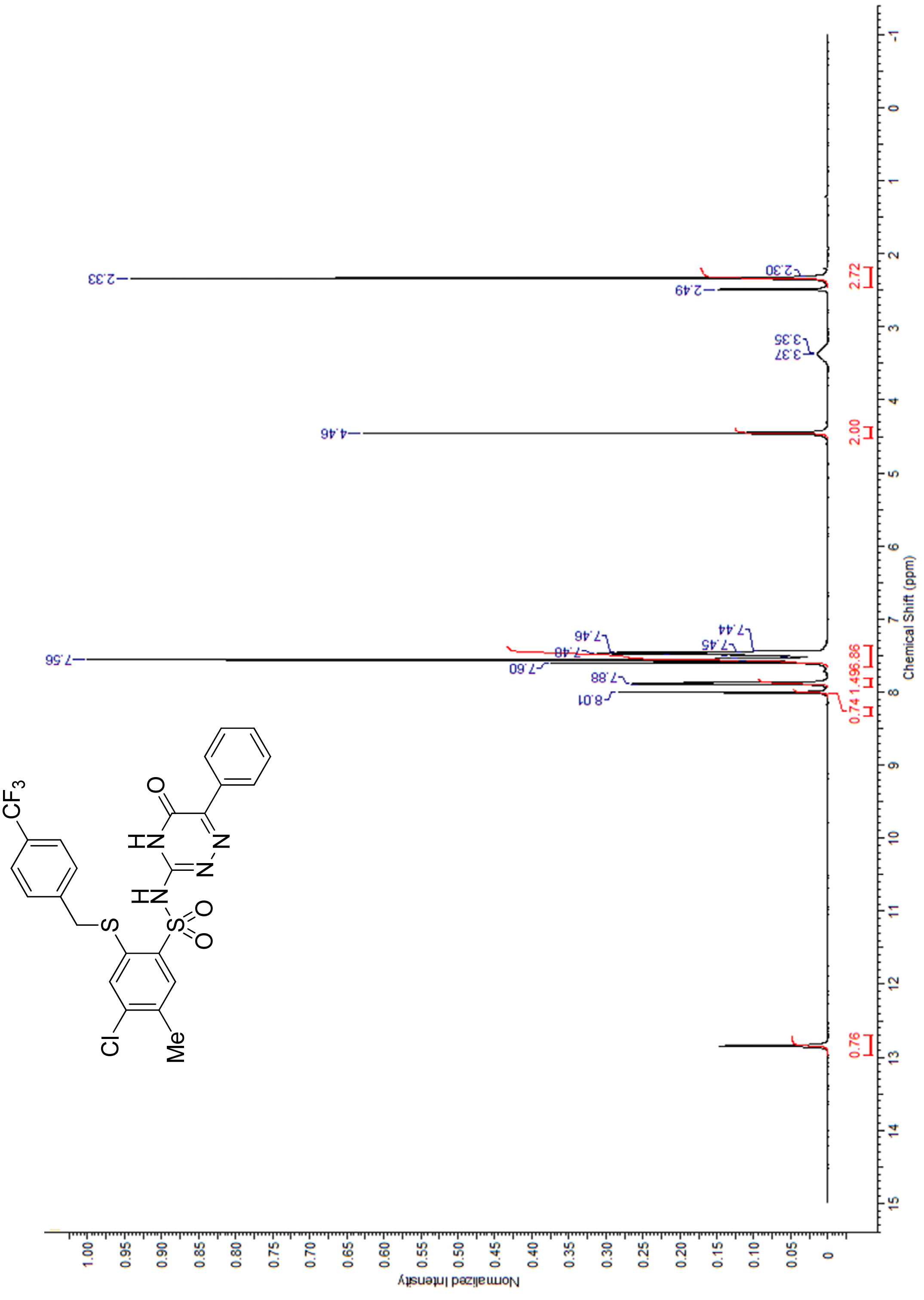

Spectrum S14. ^{1}H NMR of compd **8** (500 MHz. DMSO-d_6).

No.	(ppm)	(Hz)
1	19.66	2471.4
2	36.51	4589.2
3	39.68	4987.5
4	39.84	5008.1
5	40.01	5029.2
6	40.18	5050.3
7	40.35	5071.3
8	40.43	5082.3
9	40.51	5092.0
10	40.68	5113.1
11	123.69	15547.2
12	125.92	15826.9
13	125.95	15830.8
14	128.37	16135.6
15	128.63	16167.2
16	128.86	16196.6
17	128.88	16199.2
18	129.41	16265.5
19	130.43	16393.8
20	130.98	16462.7
21	131.40	16515.4
22	131.98	16588.3
23	133.71	16806.1
24	135.57	17040.1
25	138.08	17355.0
26	139.34	17513.5
27	142.04	17853.4
28	145.63	18303.9
29	148.83	18706.5
30	154.23	19385.8

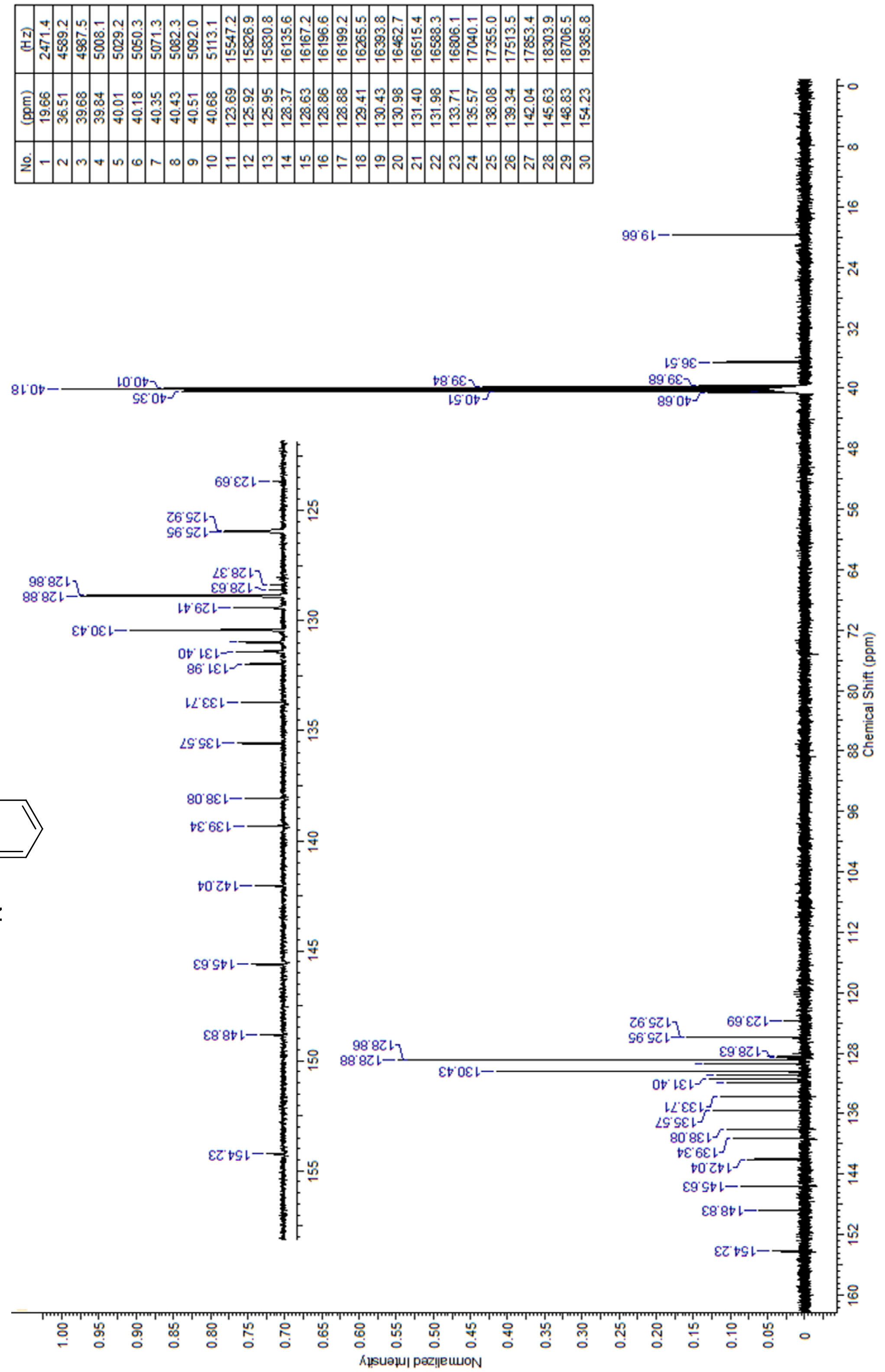

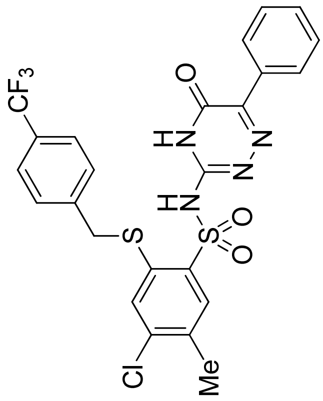

Spectrum S15. ^{13}C NMR of compd **8** (125 MHz. DMSO-d_6).

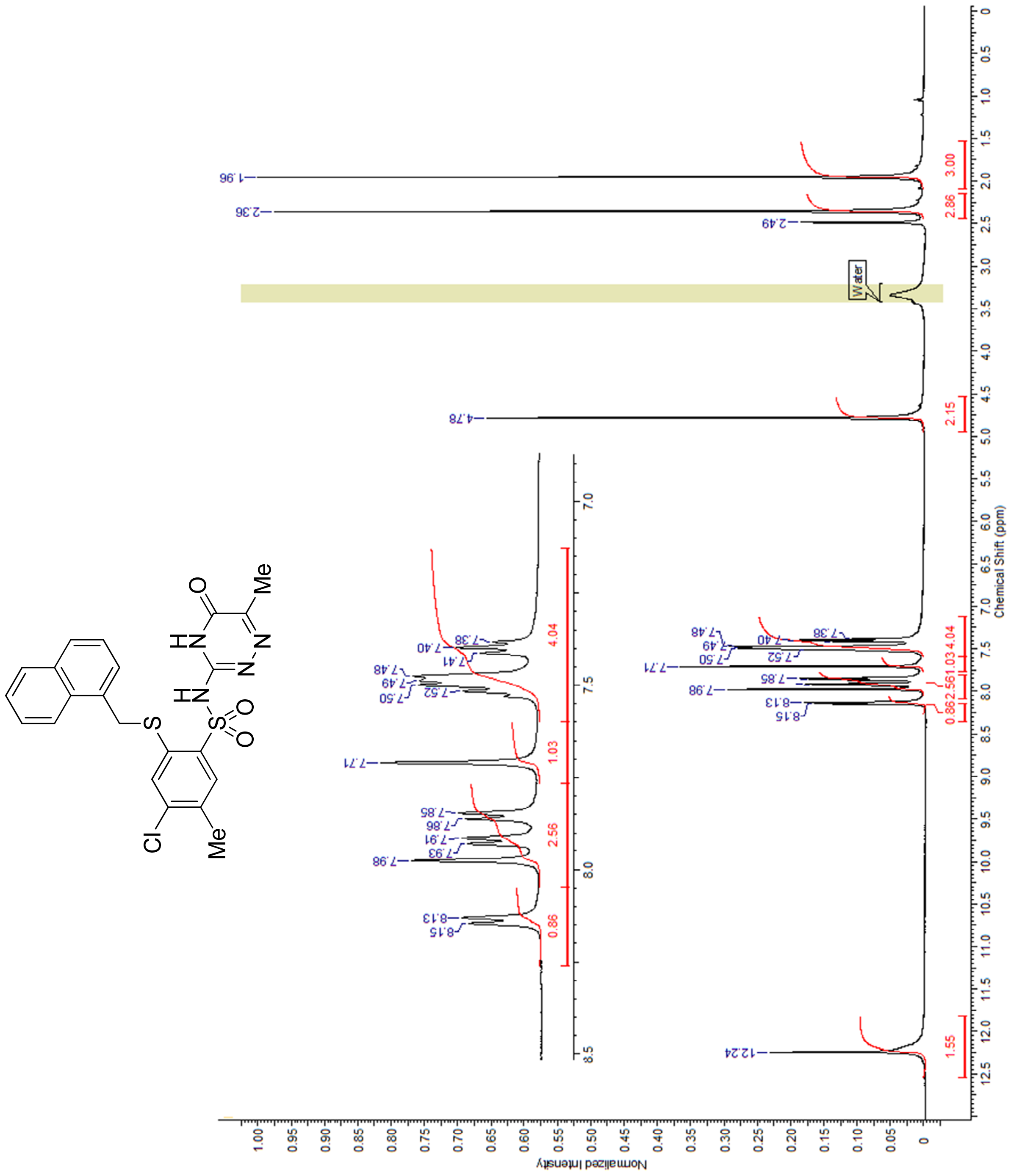

Spectrum S16. ^{1}H NMR of compd **9** (500 MHz. DMSO-d_6).

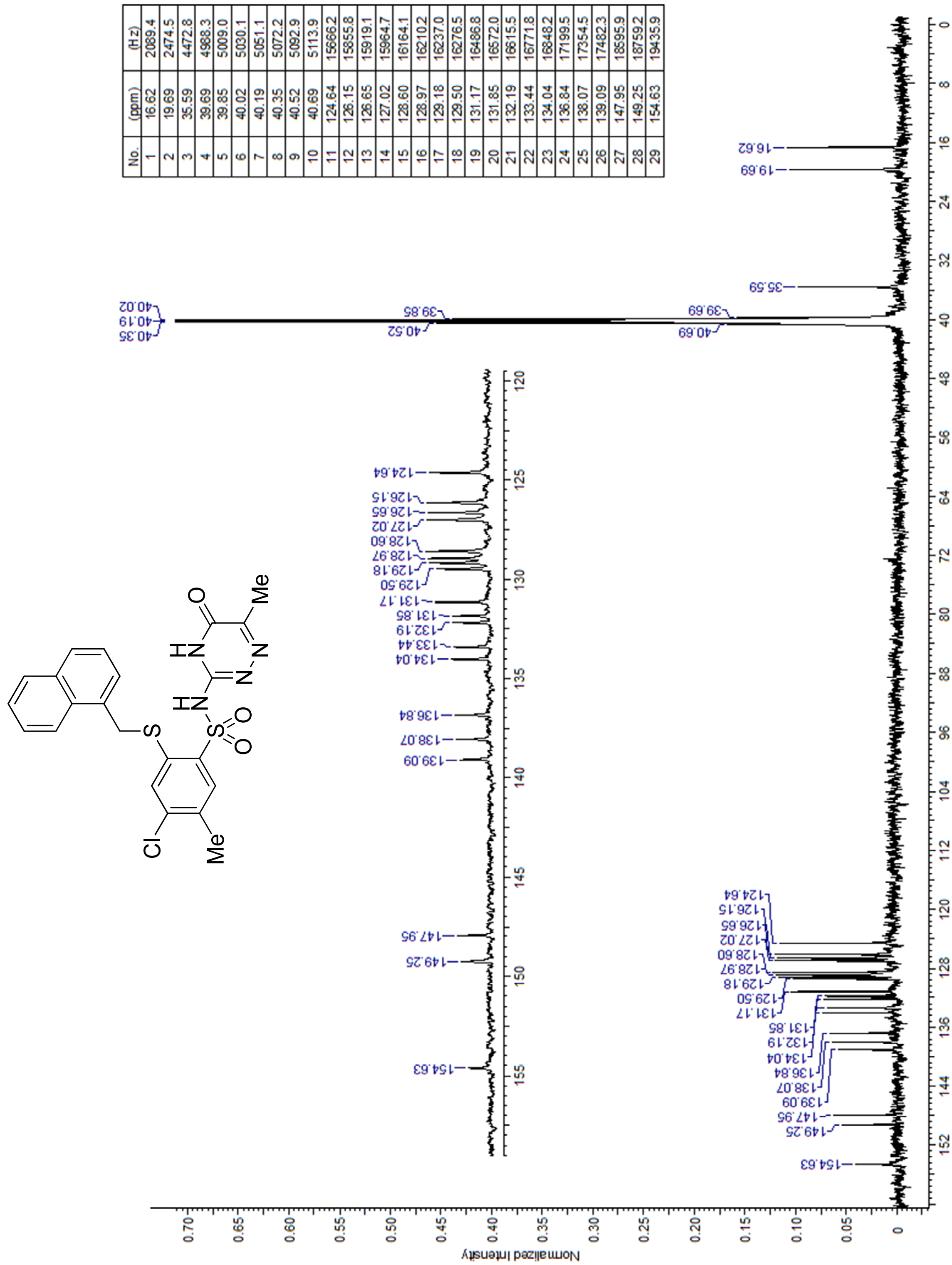

No.	(ppm)	(Hz)
1	16.62	2089.4
2	19.69	2474.5
3	35.59	4472.8
4	39.69	4988.3
5	39.85	5009.0
6	40.02	5030.1
7	40.19	5051.1
8	40.35	5072.2
9	40.52	5092.9
10	40.69	5113.9
11	124.64	15666.2
12	126.15	15855.8
13	126.65	15919.1
14	127.02	15964.7
15	128.60	16164.1
16	128.97	16210.2
17	129.18	16237.0
18	129.50	16276.5
19	131.17	16486.8
20	131.85	16572.0
21	132.19	16615.5
22	133.44	16771.8
23	134.04	16848.2
24	136.84	17199.5
25	138.07	17354.5
26	139.09	17482.3
27	147.95	18595.9
28	149.25	18759.2
29	154.63	19435.9

Spectrum S17. ^{13}C NMR of compd **9** (125 MHz. DMSO-d_6).

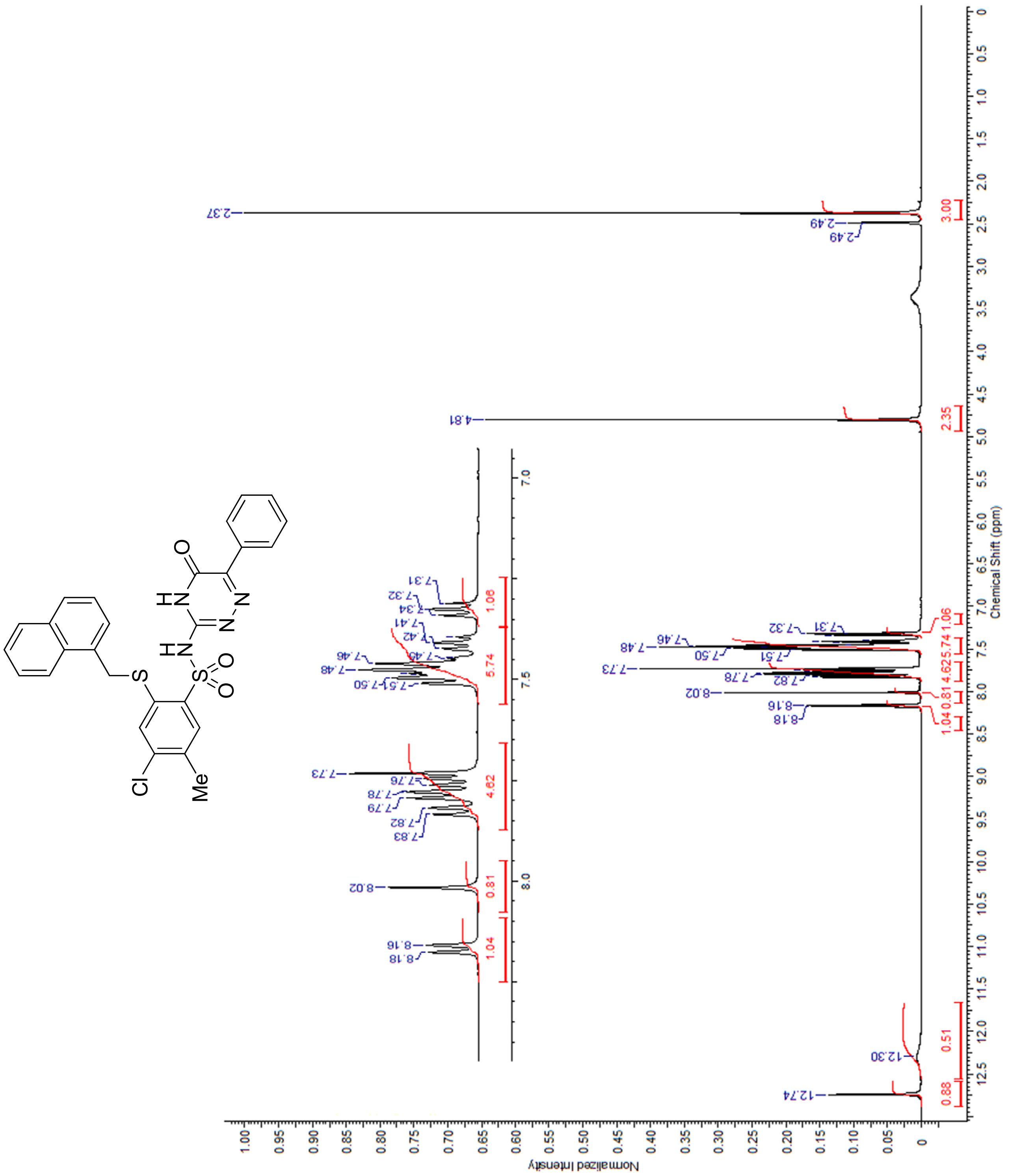

Spectrum S18. ^{1}H NMR of compd **10** (500 MHz. DMSO-d_6).

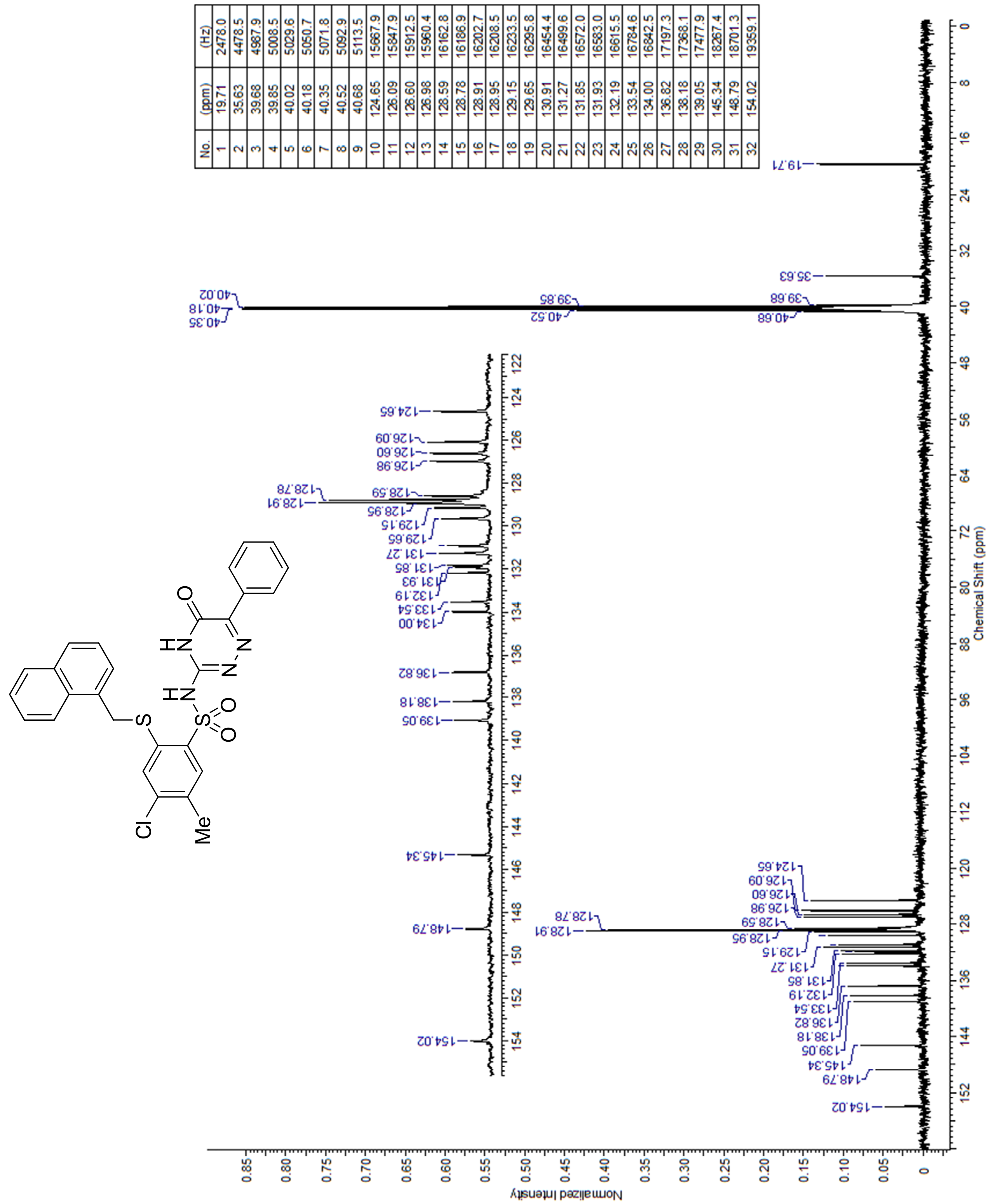

No.	(ppm)	(Hz)
1	19.71	2478.0
2	35.63	4478.5
3	39.68	4987.9
4	39.85	5008.5
5	40.02	5029.6
6	40.18	5050.7
7	40.35	5071.8
8	40.52	5092.9
9	40.68	5113.5
10	124.65	15667.9
11	126.09	15847.9
12	126.60	15912.5
13	126.98	15960.4
14	128.59	16162.8
15	128.78	16186.9
16	128.91	16202.7
17	128.95	16208.5
18	129.15	16233.5
19	129.65	16295.8
20	130.91	16454.4
21	131.27	16499.6
22	131.85	16572.0
23	131.93	16583.0
24	132.19	16615.5
25	133.54	16784.6
26	134.00	16842.5
27	136.82	17197.3
28	138.18	17368.1
29	139.05	17477.9
30	145.34	18267.4
31	148.79	18701.3
32	154.02	19359.1

Spectrum S19. ^{13}C NMR of compd **10** (125 MHz. DMSO-d_6).

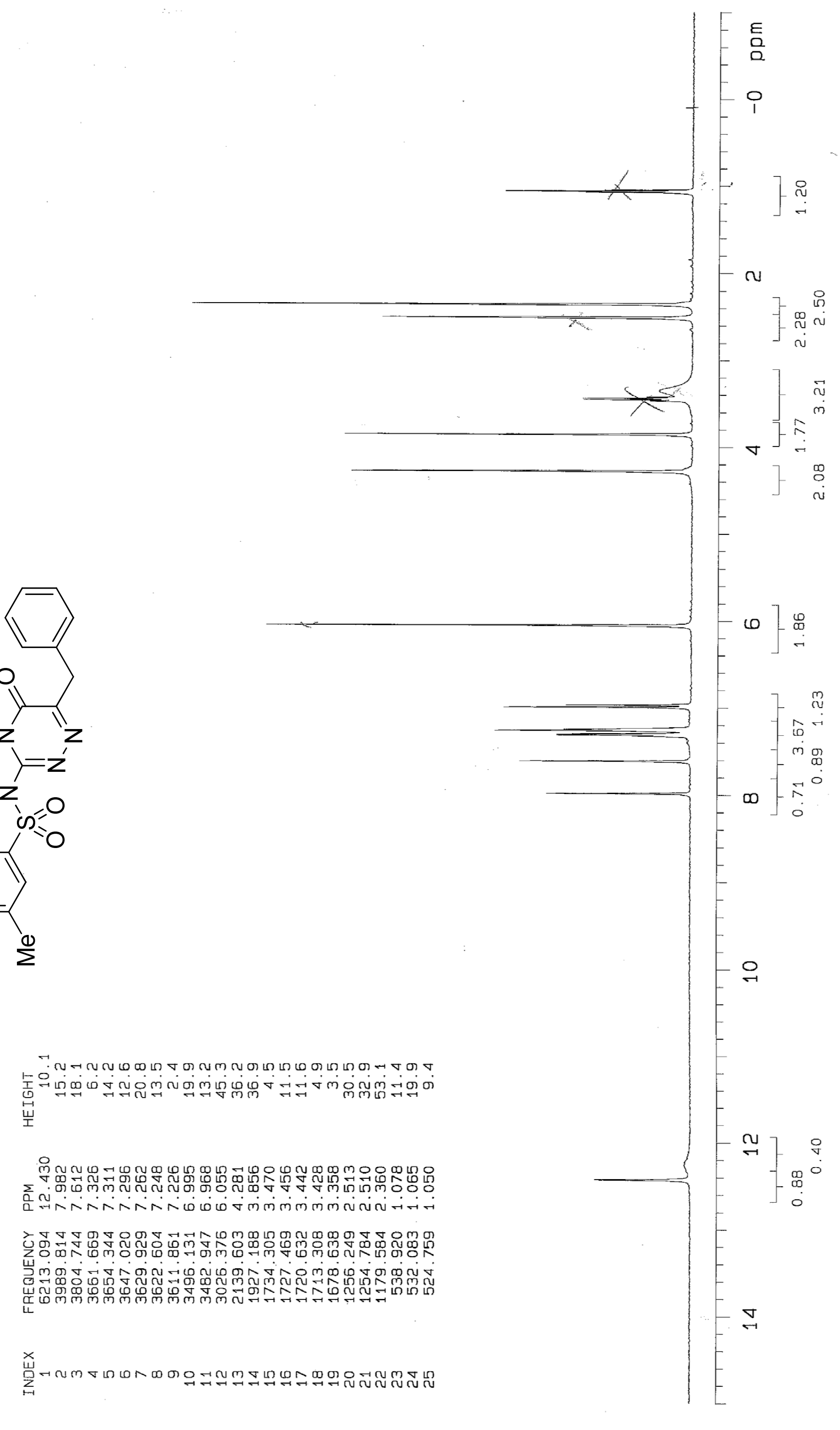

Spectrum S20. ^{1}H NMR of compd **11** (200 MHz. DMSO-*d*$_6$).

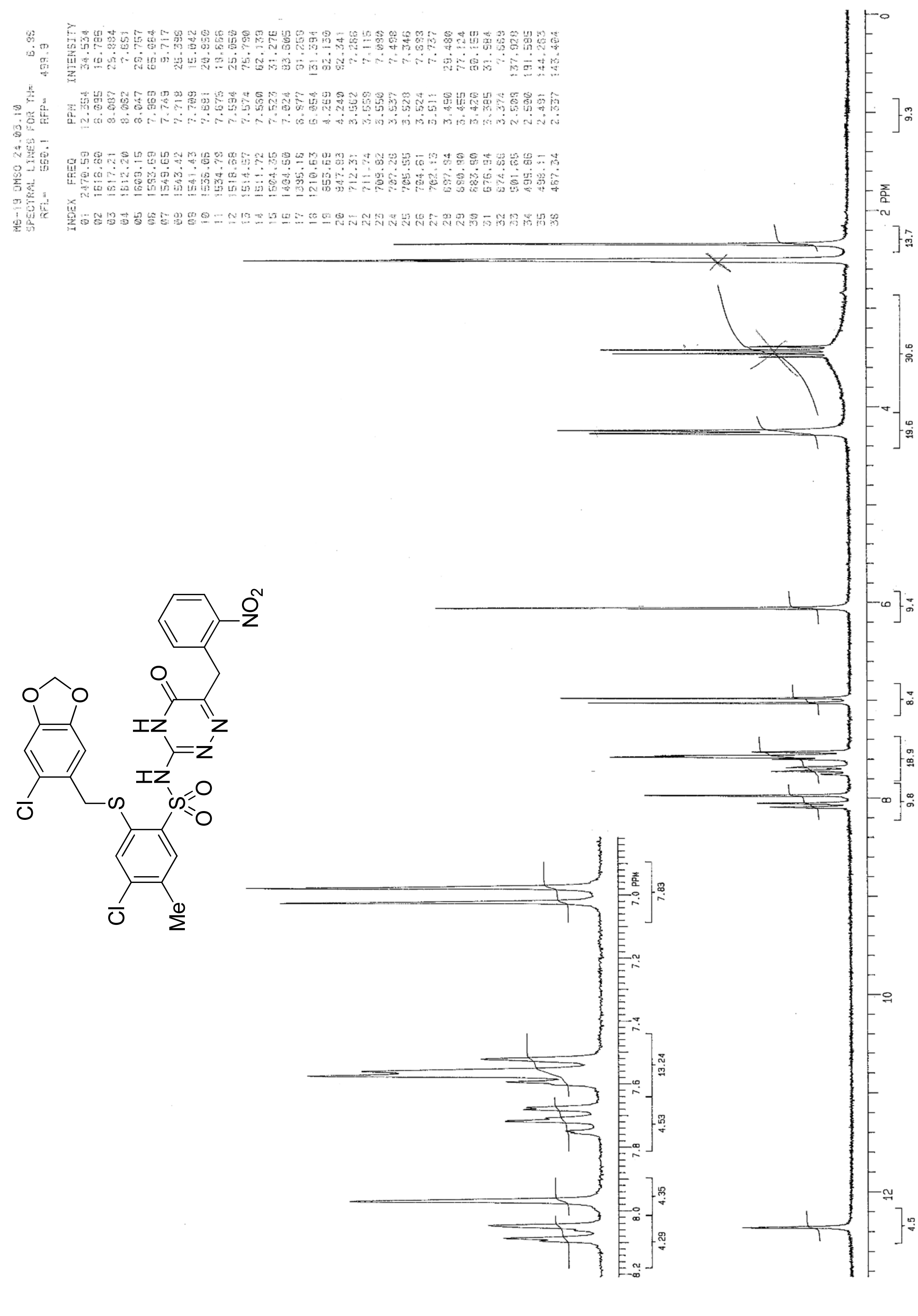

Spectrum S21. ^{1}H NMR of compd **12** (200 MHz. DMSO-d_6).

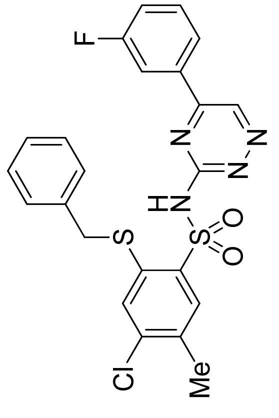

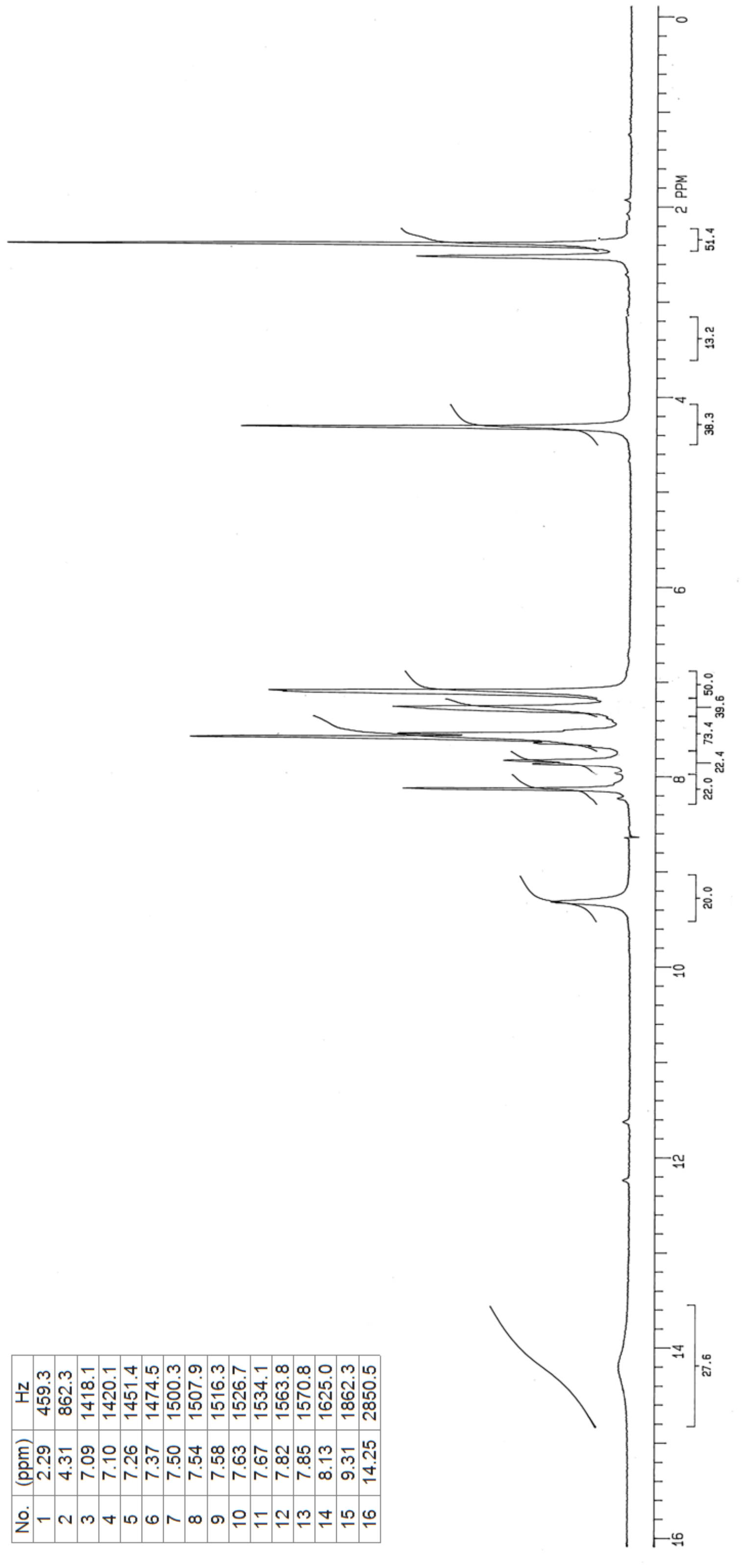

No.	(ppm)	Hz
1	2.29	459.3
2	4.31	862.3
3	7.09	1418.1
4	7.10	1420.1
5	7.26	1451.4
6	7.37	1474.5
7	7.50	1500.3
8	7.54	1507.9
9	7.58	1516.3
10	7.63	1526.7
11	7.67	1534.1
12	7.82	1563.8
13	7.85	1570.8
14	8.13	1625.0
15	9.31	1862.3
16	14.25	2850.5

Spectrum S22. ^{1}H NMR of compd **13** (200 MHz. DMSO-*d6*).

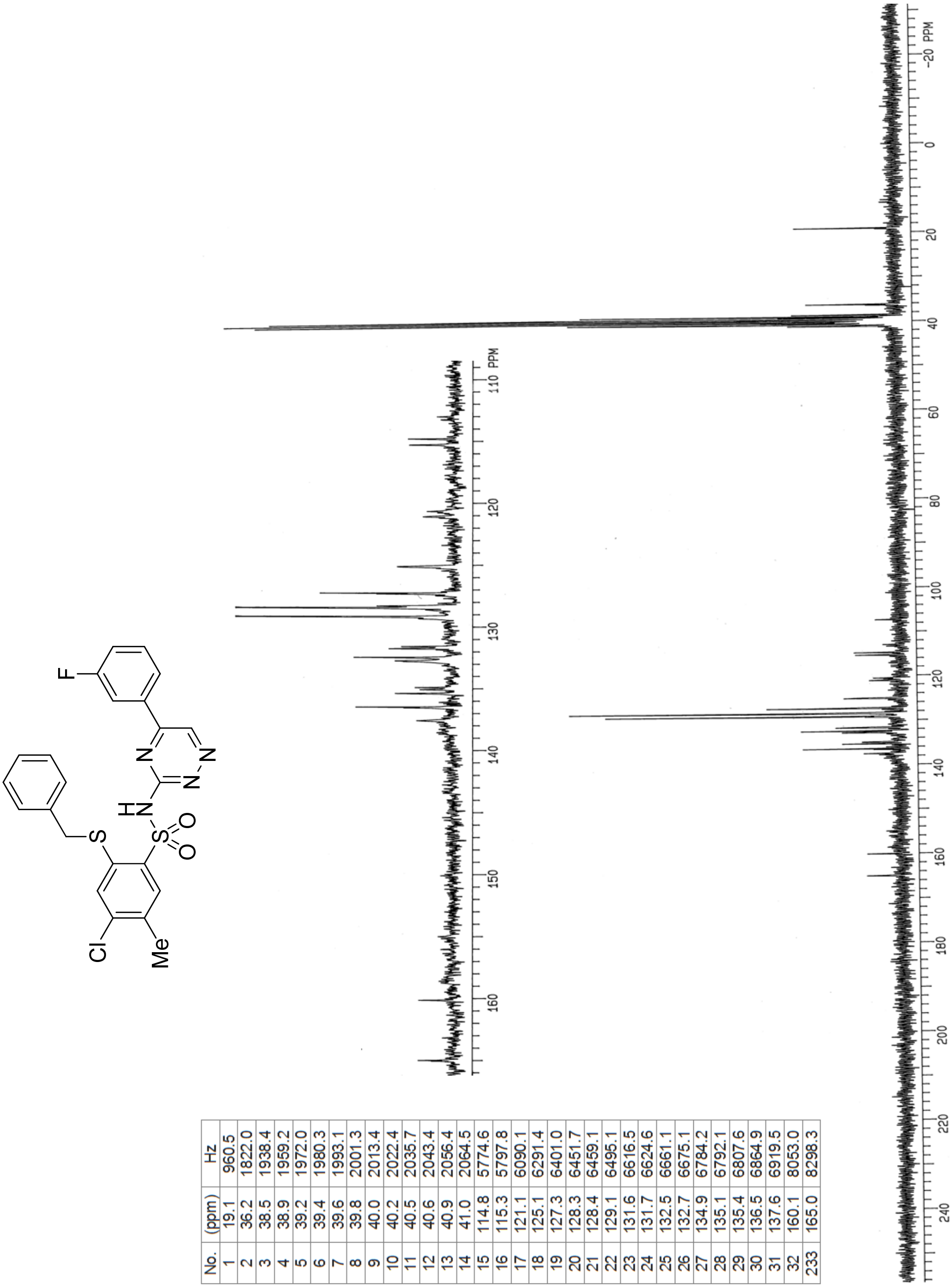

No.	(ppm)	Hz
1	19.1	960.5
2	36.2	1822.0
3	38.5	1938.4
4	38.9	1959.2
5	39.2	1972.0
6	39.4	1980.3
7	39.6	1993.1
8	39.8	2001.3
9	40.0	2013.4
10	40.2	2022.4
11	40.5	2035.7
12	40.6	2043.4
13	40.9	2056.4
14	41.0	2064.5
15	114.8	5774.6
16	115.3	5797.8
17	121.1	6090.1
18	125.1	6291.4
19	127.3	6401.0
20	128.3	6451.7
21	128.4	6459.1
22	129.1	6495.1
23	131.6	6616.5
24	131.7	6624.6
25	132.5	6661.1
26	132.7	6675.1
27	134.9	6784.2
28	135.1	6792.1
29	135.4	6807.6
30	136.5	6864.9
31	137.6	6919.5
32	160.1	8053.0
233	165.0	8298.3

Spectrum S23. ^{13}C NMR of compd **13** (50 MHz. DMSO-d_6).

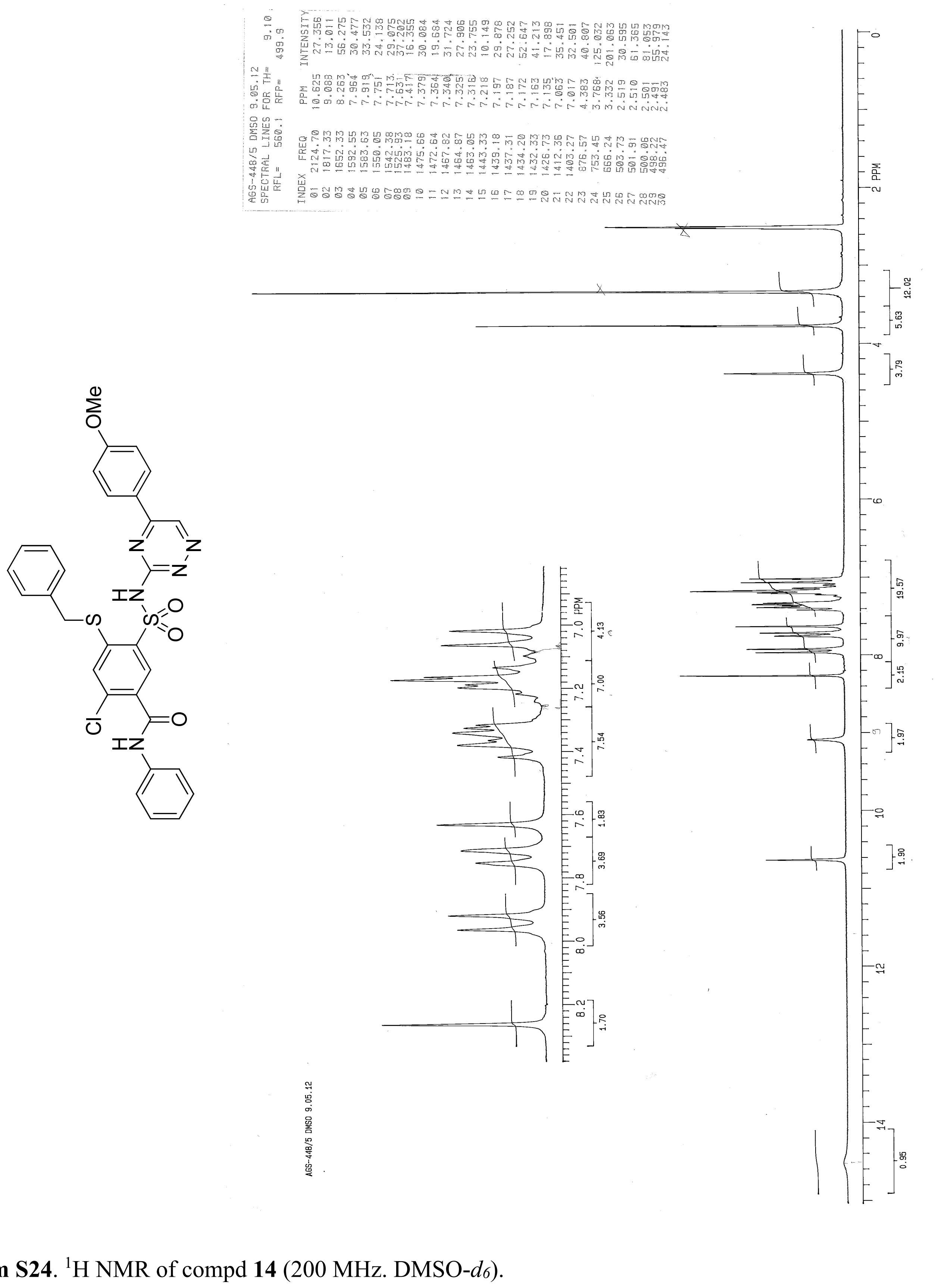

Spectrum S24. ^{1}H NMR of compd **14** (200 MHz. DMSO-d_6).

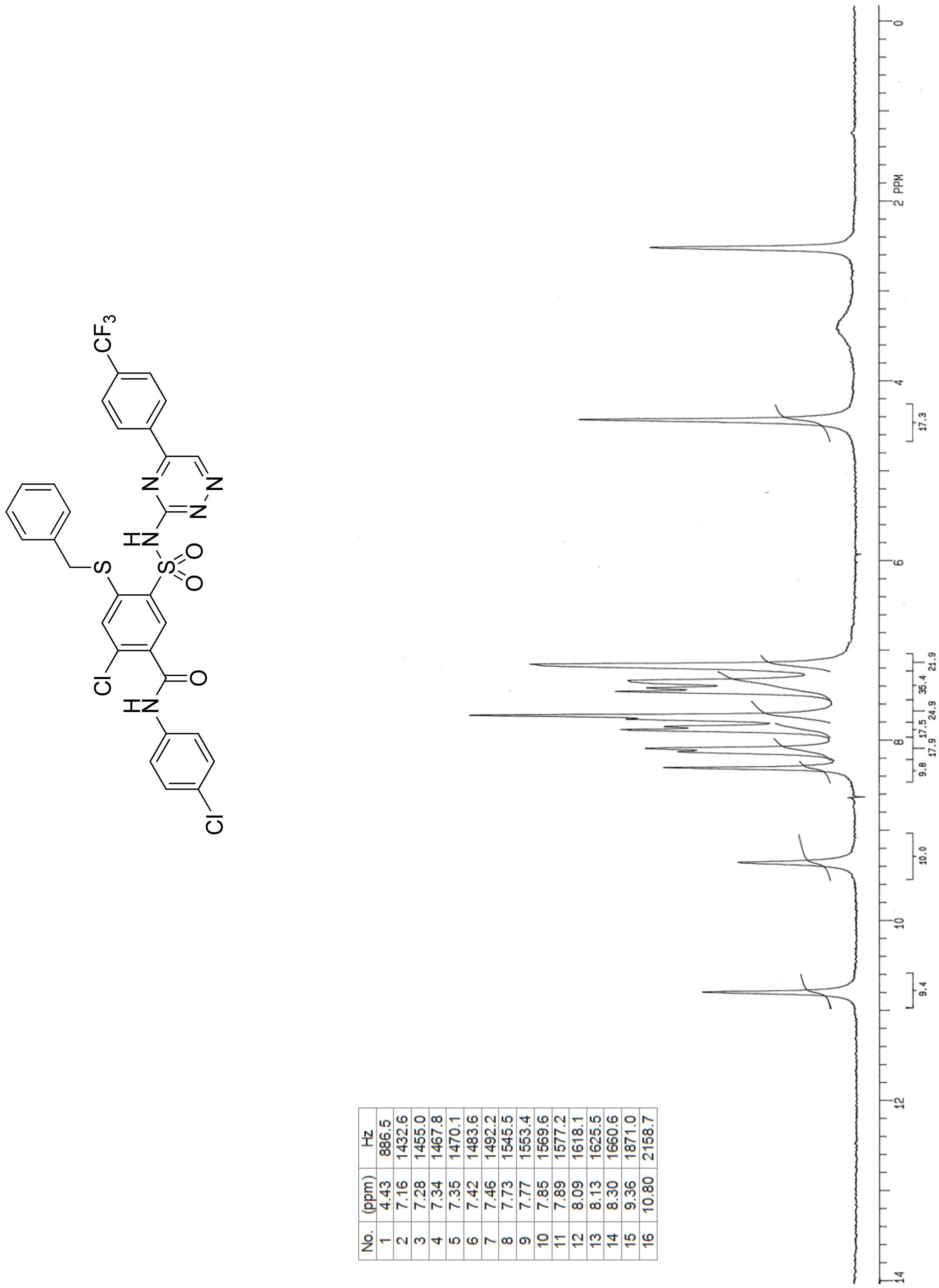

No.	(ppm)	Hz
1	4.43	886.5
2	7.16	1432.6
3	7.28	1455.0
4	7.34	1467.8
5	7.35	1470.1
6	7.42	1483.6
7	7.46	1492.2
8	7.73	1545.5
9	7.77	1553.4
10	7.85	1569.6
11	7.89	1577.2
12	8.09	1618.1
13	8.13	1625.5
14	8.30	1660.6
15	9.36	1871.0
16	10.80	2158.7

Spectrum S25. ^{1}H NMR of compd **15** (200 MHz. DMSO-d_6).

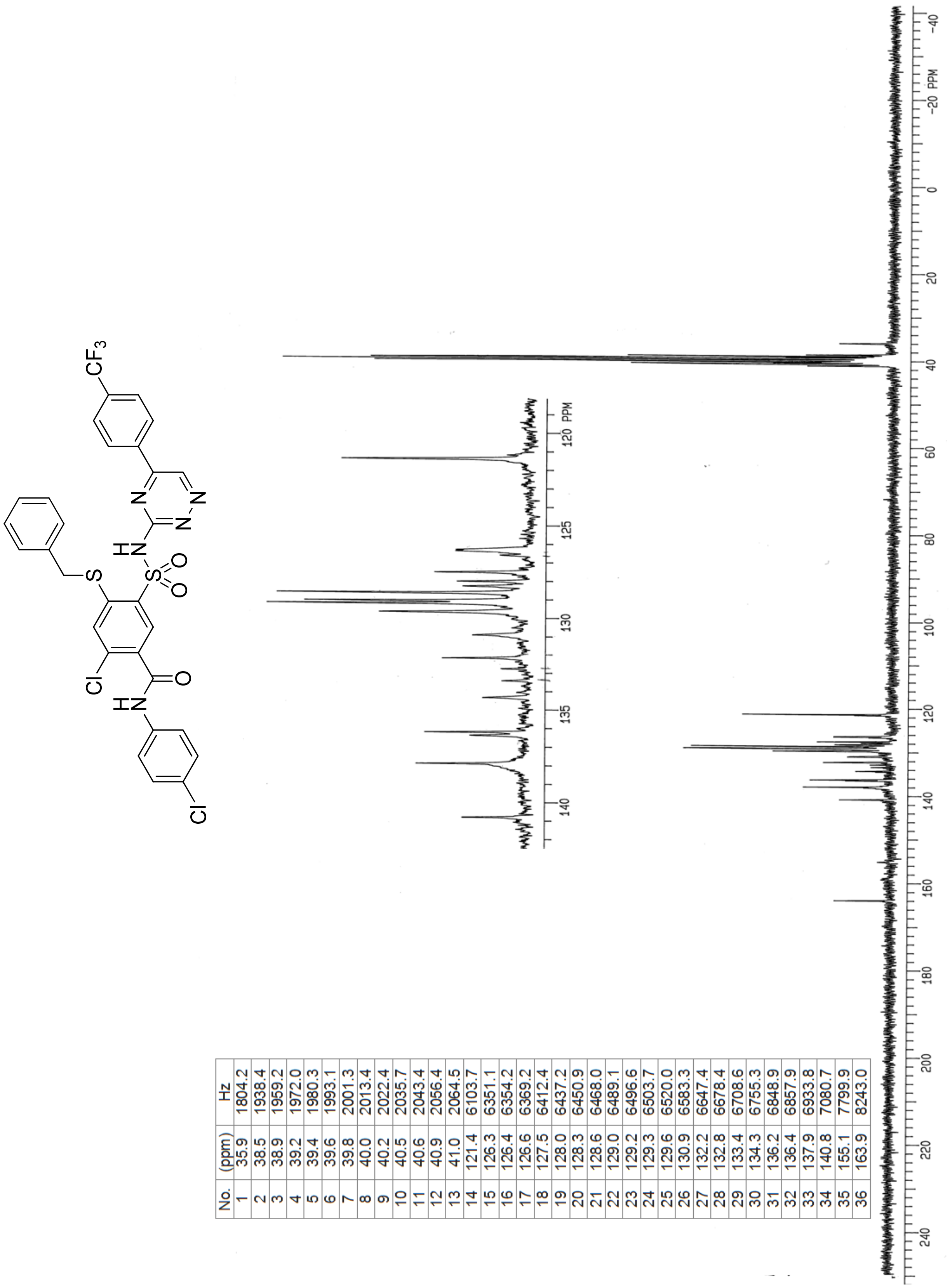

No.	(ppm)	Hz
1	35.9	1804.2
2	38.5	1938.4
3	38.9	1959.2
4	39.2	1972.0
5	39.4	1980.3
6	39.6	1993.1
7	39.8	2001.3
8	40.0	2013.4
9	40.2	2022.4
10	40.5	2035.7
11	40.6	2043.4
12	40.9	2056.4
13	41.0	2064.5
14	121.4	6103.7
15	126.3	6351.1
16	126.4	6354.2
17	126.6	6369.2
18	127.5	6412.4
19	128.0	6437.2
20	128.3	6450.9
21	128.6	6468.0
22	129.0	6489.1
23	129.2	6496.6
24	129.3	6503.7
25	129.6	6520.0
26	130.9	6583.3
27	132.2	6647.4
28	132.8	6678.4
29	133.4	6708.6
30	134.3	6755.3
31	136.2	6848.9
32	136.4	6857.9
33	137.9	6933.8
34	140.8	7080.7
35	155.1	7799.9
36	163.9	8243.0

Spectrum S26. ^{13}C NMR of compd **15** (50 MHz. DMSO-d_6).

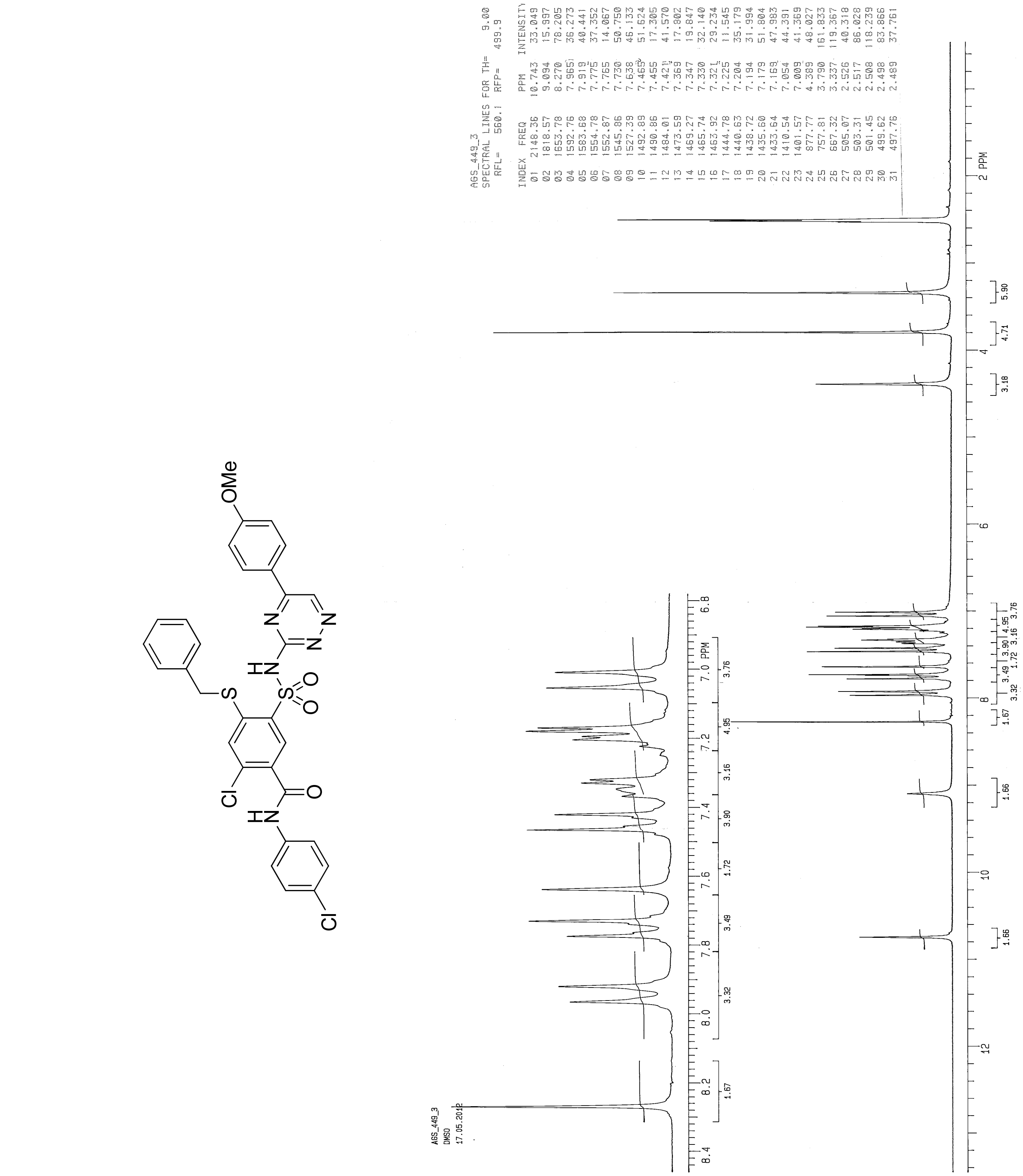

Spectrum S27. ^{1}H NMR of compd **16** (200 MHz. DMSO-d_6).

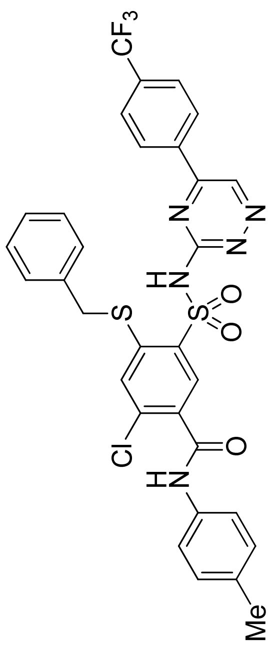

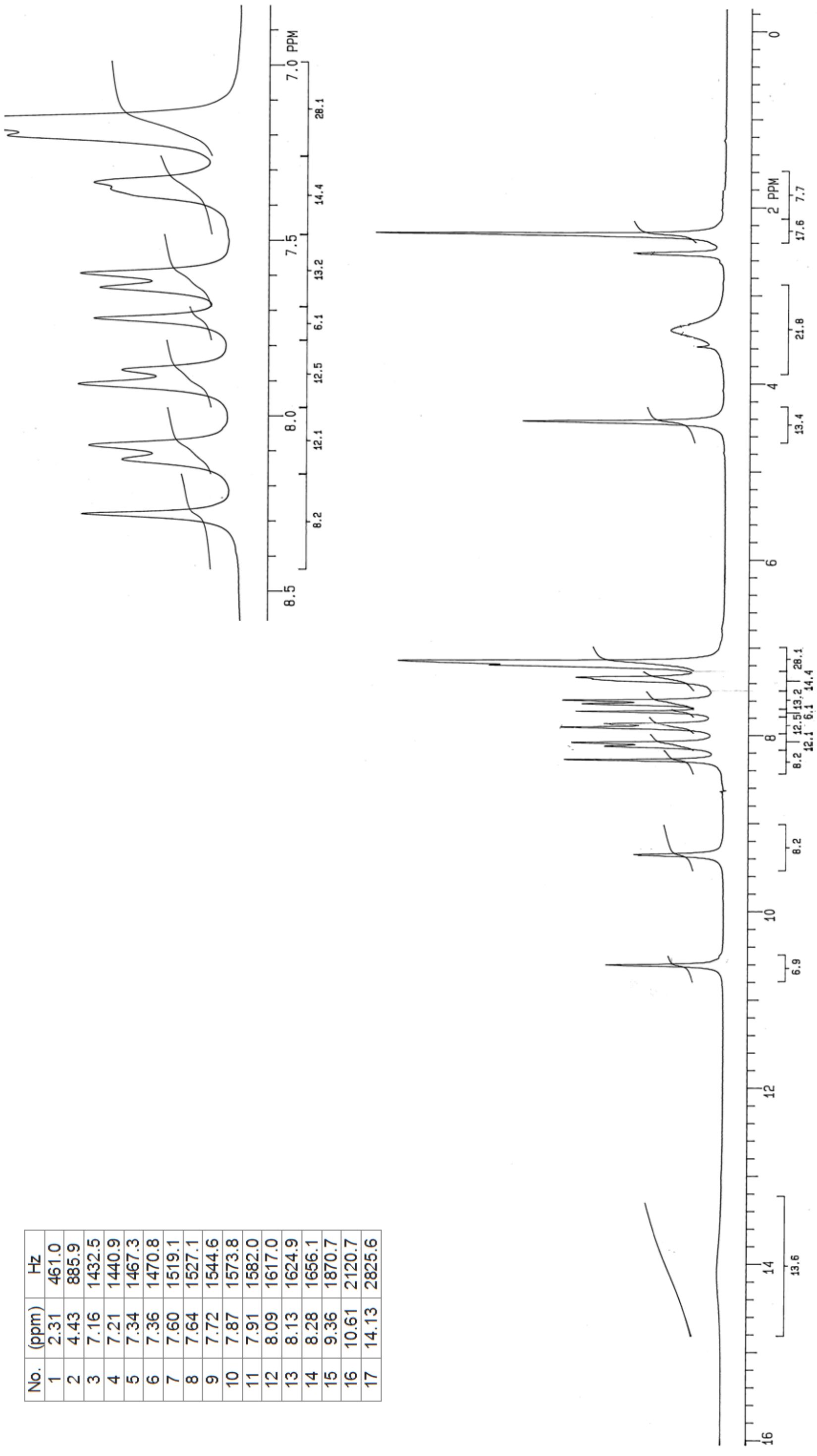

No.	(ppm)	Hz
1	2.31	461.0
2	4.43	885.9
3	7.16	1432.5
4	7.21	1440.9
5	7.34	1467.3
6	7.36	1470.8
7	7.60	1519.1
8	7.64	1527.1
9	7.72	1544.6
10	7.87	1573.8
11	7.91	1582.0
12	8.09	1617.0
13	8.13	1624.9
14	8.28	1656.1
15	9.36	1870.7
16	10.61	2120.7
17	14.13	2825.6

Spectrum S28. ^{1}H NMR of compd **17** (200 MHz. DMSO-*d*$_6$).

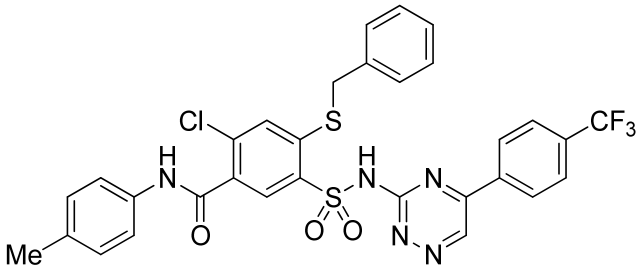

No.	(ppm)	Hz
1	20.8	1045.0
2	35.9	1805.9
3	38.5	1938.4
4	38.9	1959.2
5	39.2	1972.0
6	39.4	1980.3
7	39.6	1993.1
8	39.8	2001.3
9	40.0	2013.4
10	40.2	2022.4
11	40.5	2035.7
12	40.6	2043.4
13	40.9	2056.4
14	41.0	2064.5
15	119.8	6026.3
16	126.3	6352.9
17	126.4	6356.4
18	127.5	6411.3
19	128.3	6452.7
20	128.5	6459.9
21	128.6	6467.1
22	129.2	6496.5
23	129.5	6510.6
24	129.6	6519.5
25	130.9	6584.3
26	132.6	6667.3
27	133.4	6708.1
28	134.4	6757.7
29	136.2	6849.8
30	136.4	6858.6
31	136.5	6862.9
32	136.9	6884.6
33	140.5	7065.3
34	155.0	7794.9
35	163.6	8229.1

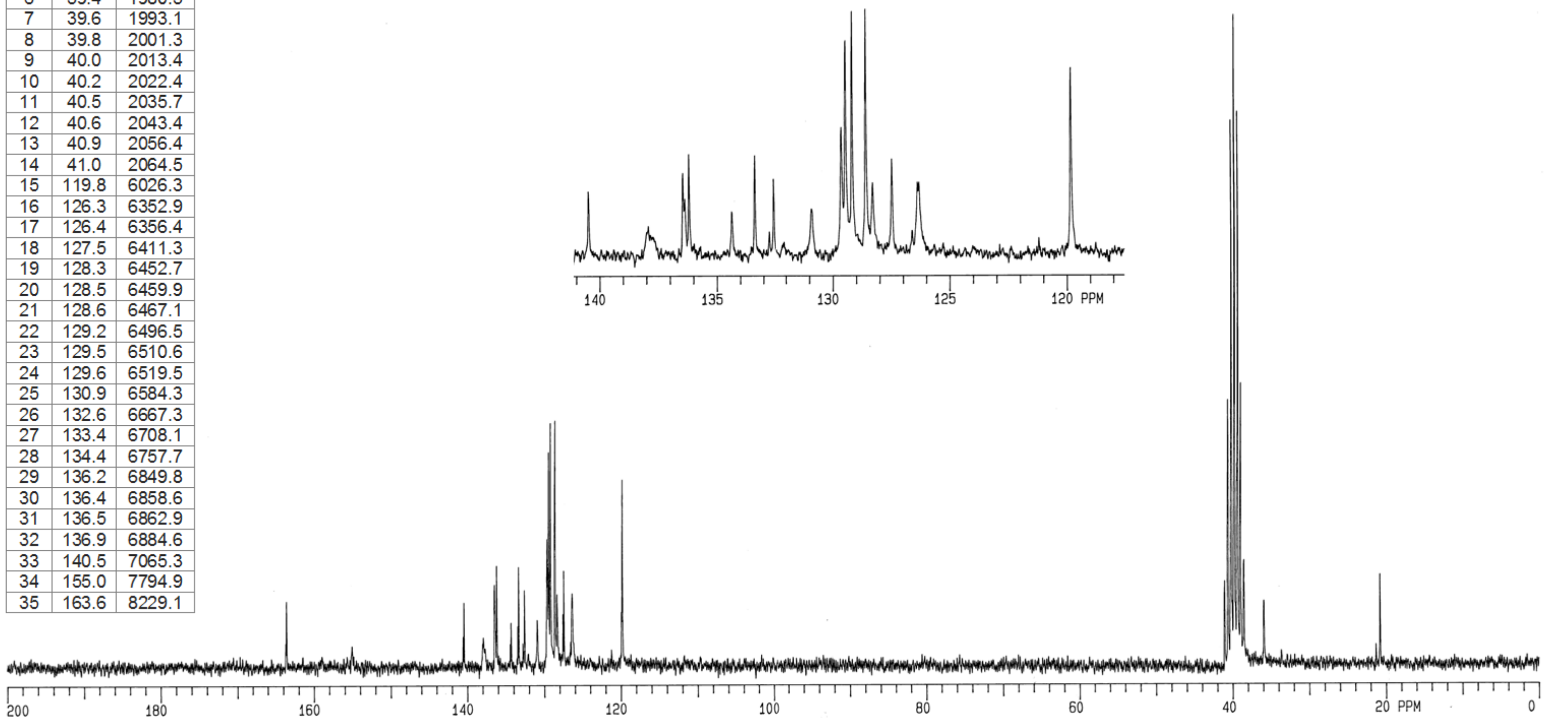

Spectrum S29. ^{13}C NMR of compd **17** (50 MHz. DMSO-d_6).

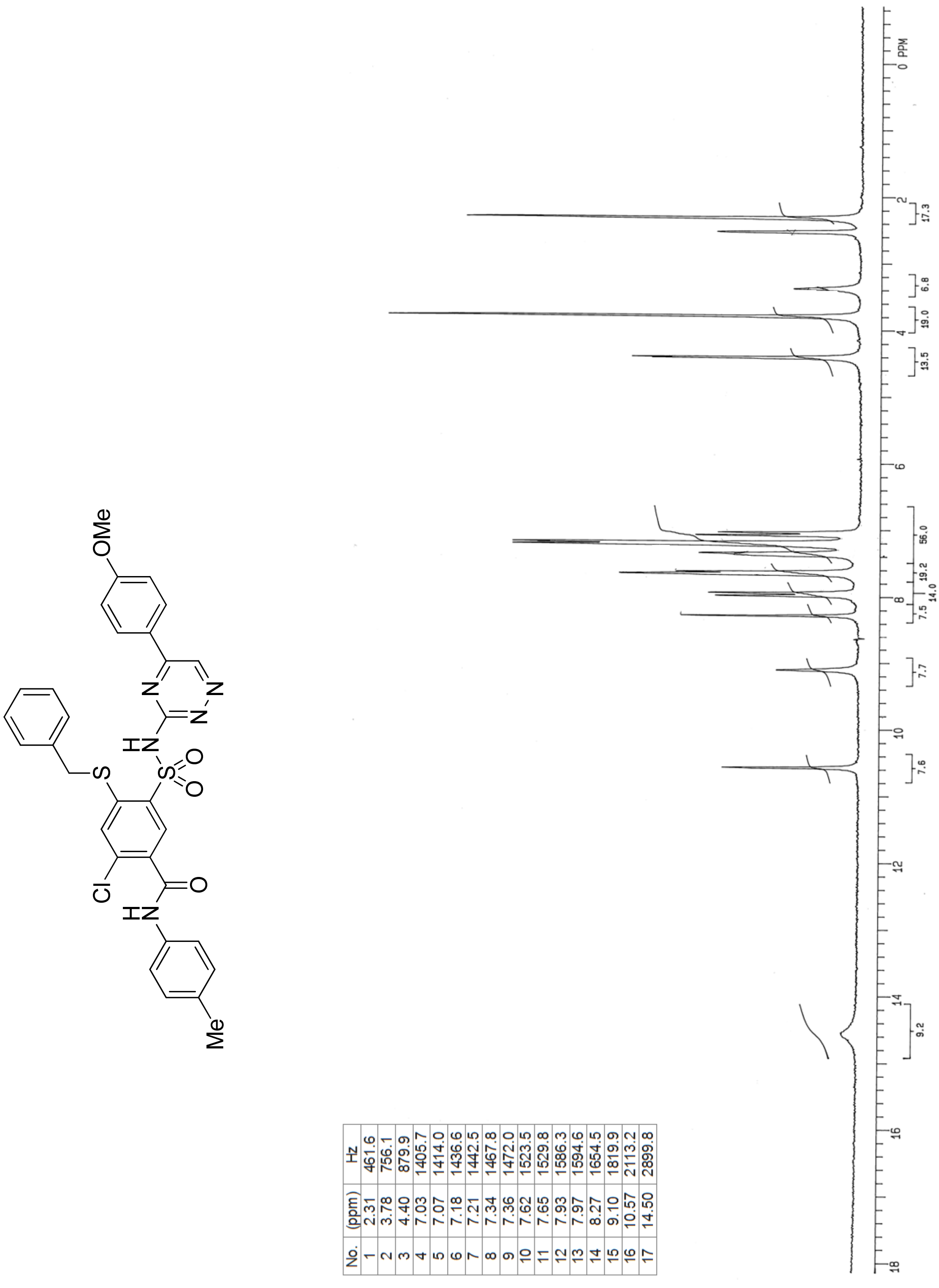

No.	(ppm)	Hz
1	2.31	461.6
2	3.78	756.1
3	4.40	879.9
4	7.03	1405.7
5	7.07	1414.0
6	7.18	1436.6
7	7.21	1442.5
8	7.34	1467.8
9	7.36	1472.0
10	7.62	1523.5
11	7.65	1529.8
12	7.93	1586.3
13	7.97	1594.6
14	8.27	1654.5
15	9.10	1819.9
16	10.57	2113.2
17	14.50	2899.8

Spectrum S30. ^{1}H NMR of compd **18** (200 MHz. DMSO-d_6).

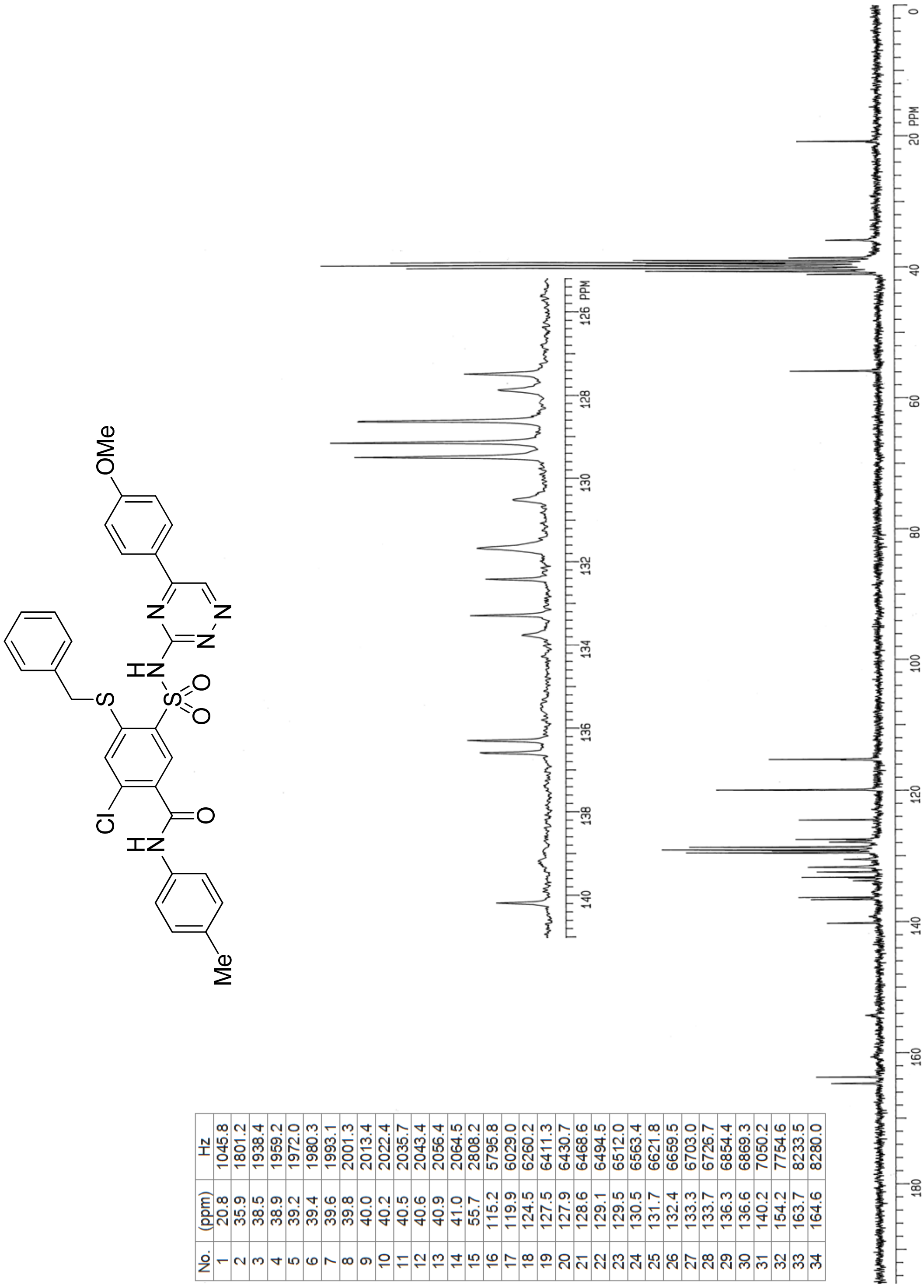

No.	(ppm)	Hz
1	20.8	1045.8
2	35.9	1801.2
3	38.5	1938.4
4	38.9	1959.2
5	39.2	1972.0
6	39.4	1980.3
7	39.6	1993.1
8	39.8	2001.3
9	40.0	2013.4
10	40.2	2022.4
11	40.5	2035.7
12	40.6	2043.4
13	40.9	2056.4
14	41.0	2064.5
15	55.7	2808.2
16	115.2	5795.8
17	119.9	6029.0
18	124.5	6260.2
19	127.5	6411.3
20	127.9	6430.7
21	128.6	6468.6
22	129.1	6494.5
23	129.5	6512.0
24	130.5	6563.4
25	131.7	6621.8
26	132.4	6659.5
27	133.3	6703.0
28	133.7	6726.7
29	136.3	6854.4
30	136.6	6869.3
31	140.2	7050.2
32	154.2	7754.6
33	163.7	8233.5
34	164.6	8280.0

Spectrum S31. ^{13}C NMR of compd **18** (50 MHz. DMSO-d_6).

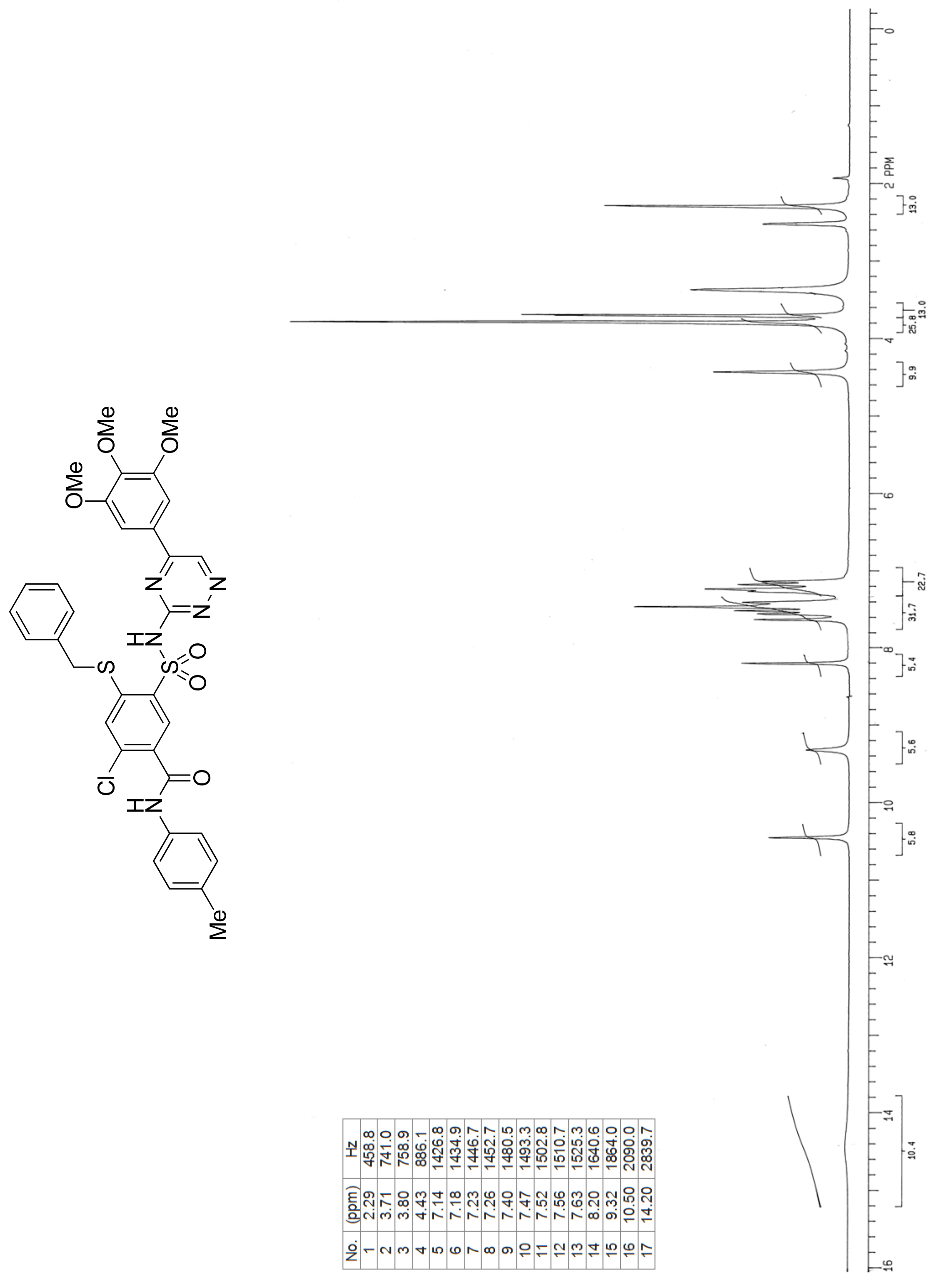

No.	(ppm)	Hz
1	2.29	458.8
2	3.71	741.0
3	3.80	758.9
4	4.43	886.1
5	7.14	1426.8
6	7.18	1434.9
7	7.23	1446.7
8	7.26	1452.7
9	7.40	1480.5
10	7.47	1493.3
11	7.52	1502.8
12	7.56	1510.7
13	7.63	1525.3
14	8.20	1640.6
15	9.32	1864.0
16	10.50	2090.0
17	14.20	2839.7

Spectrum S32. ^{1}H NMR of compd **19** (200 MHz. DMSO-d_6).

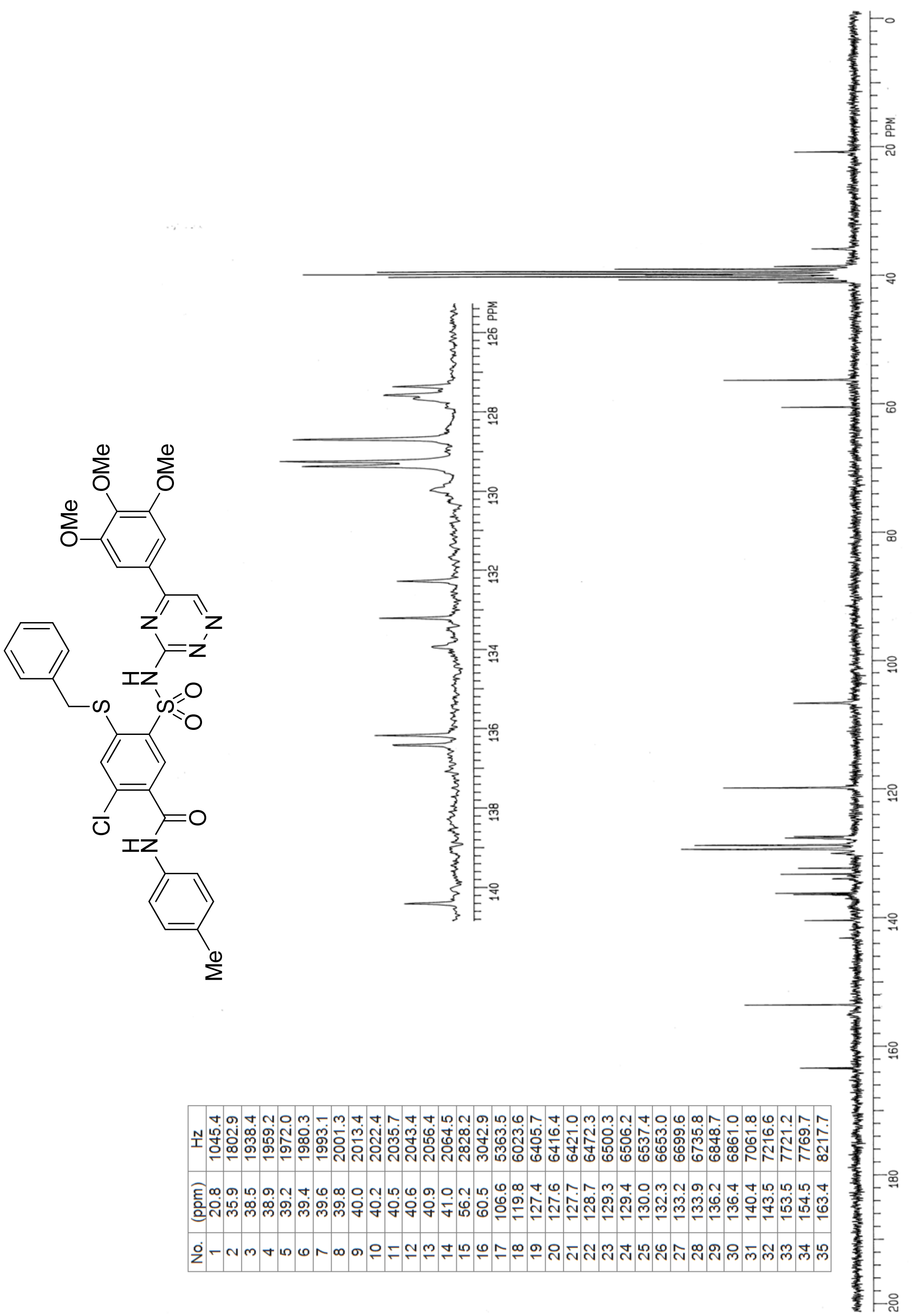

No.	(ppm)	Hz
1	20.8	1045.4
2	35.9	1802.9
3	38.5	1938.4
4	38.9	1959.2
5	39.2	1972.0
6	39.4	1980.3
7	39.6	1993.1
8	39.8	2001.3
9	40.0	2013.4
10	40.2	2022.4
11	40.5	2035.7
12	40.6	2043.4
13	40.9	2056.4
14	41.0	2064.5
15	56.2	2828.2
16	60.5	3042.9
17	106.6	5363.5
18	119.8	6023.6
19	127.4	6405.7
20	127.6	6416.4
21	127.7	6421.0
22	128.7	6472.3
23	129.3	6500.3
24	129.4	6506.2
25	130.0	6537.4
26	132.3	6653.0
27	133.2	6699.6
28	133.9	6735.8
29	136.2	6848.7
30	136.4	6861.0
31	140.4	7061.8
32	143.5	7216.6
33	153.5	7721.2
34	154.5	7769.7
35	163.4	8217.7

Spectrum S33. ^{13}C NMR of compd **19** (50 MHz. DMSO-d_6).

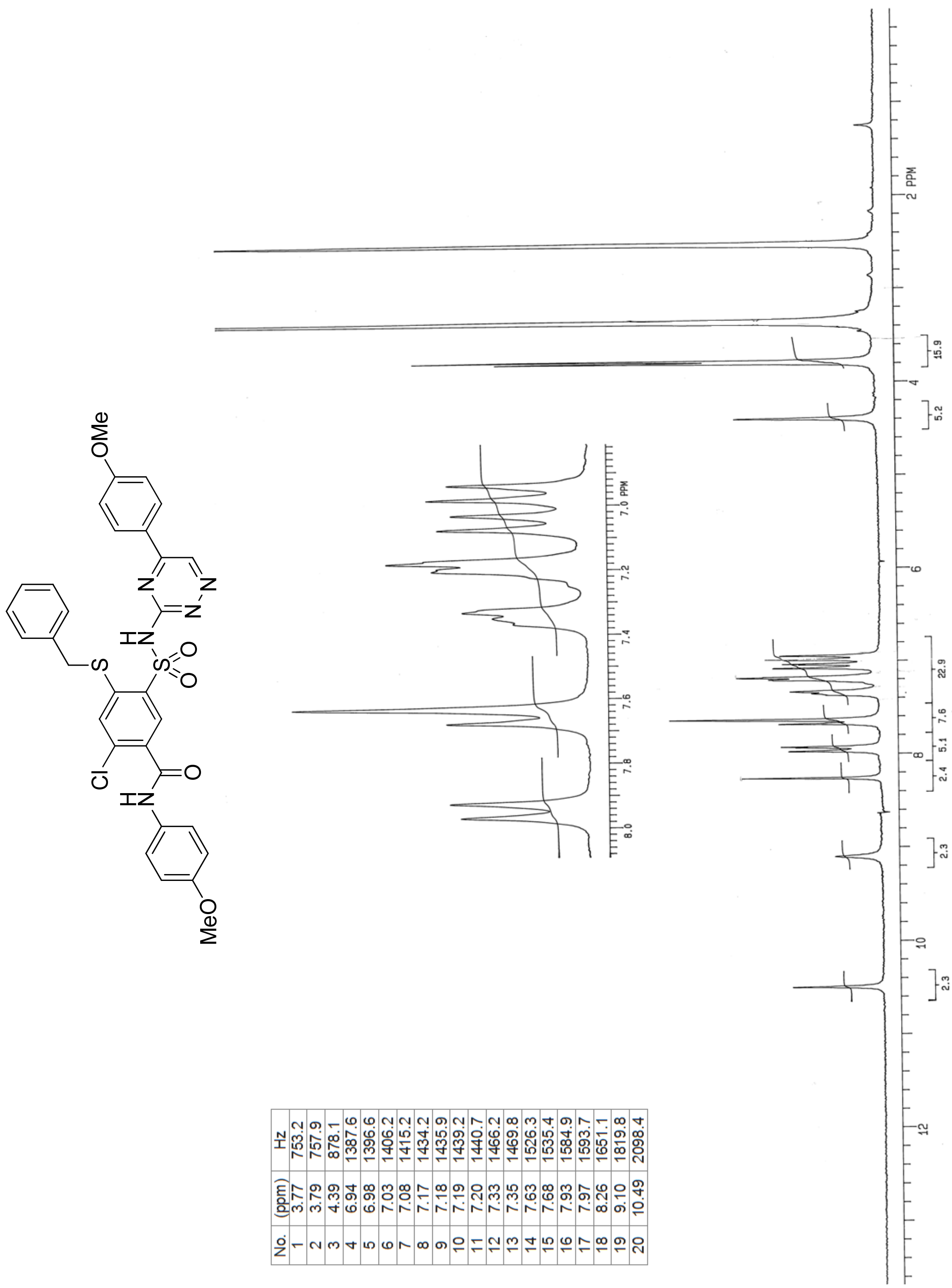

No.	(ppm)	Hz
1	3.77	753.2
2	3.79	757.9
3	4.39	878.1
4	6.94	1387.6
5	6.98	1396.6
6	7.03	1406.2
7	7.08	1415.2
8	7.17	1434.2
9	7.18	1435.9
10	7.19	1439.2
11	7.20	1440.7
12	7.33	1466.2
13	7.35	1469.8
14	7.63	1526.3
15	7.68	1535.4
16	7.93	1584.9
17	7.97	1593.7
18	8.26	1651.1
19	9.10	1819.8
20	10.49	2098.4

Spectrum S34. ^{1}H NMR of compd **20** (200 MHz. DMSO-d_6).

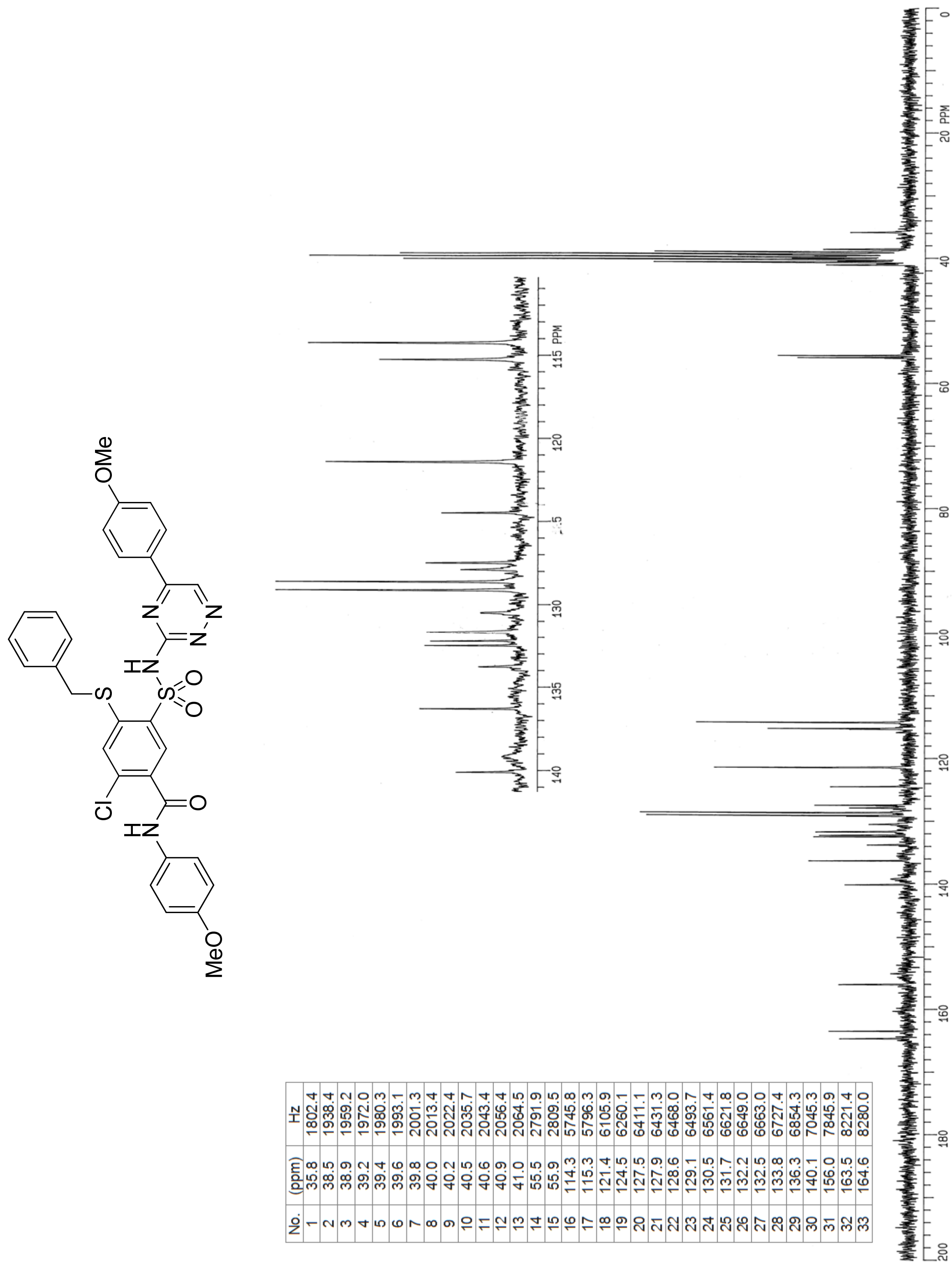

No.	(ppm)	Hz
1	35.8	1802.4
2	38.5	1938.4
3	38.9	1959.2
4	39.2	1972.0
5	39.4	1980.3
6	39.6	1993.1
7	39.8	2001.3
8	40.0	2013.4
9	40.2	2022.4
10	40.5	2035.7
11	40.6	2043.4
12	40.9	2056.4
13	41.0	2064.5
14	55.5	2791.9
15	55.9	2809.5
16	114.3	5745.8
17	115.3	5796.3
18	121.4	6105.9
19	124.5	6260.1
20	127.5	6411.1
21	127.9	6431.3
22	128.6	6468.0
23	129.1	6493.7
24	130.5	6561.4
25	131.7	6621.8
26	132.2	6649.0
27	132.5	6663.0
28	133.8	6727.4
29	136.3	6854.3
30	140.1	7045.3
31	156.0	7845.9
32	163.5	8221.4
33	164.6	8280.0

Spectrum S35. ^{13}C NMR of compd **20** (50 MHz. DMSO-d_6).

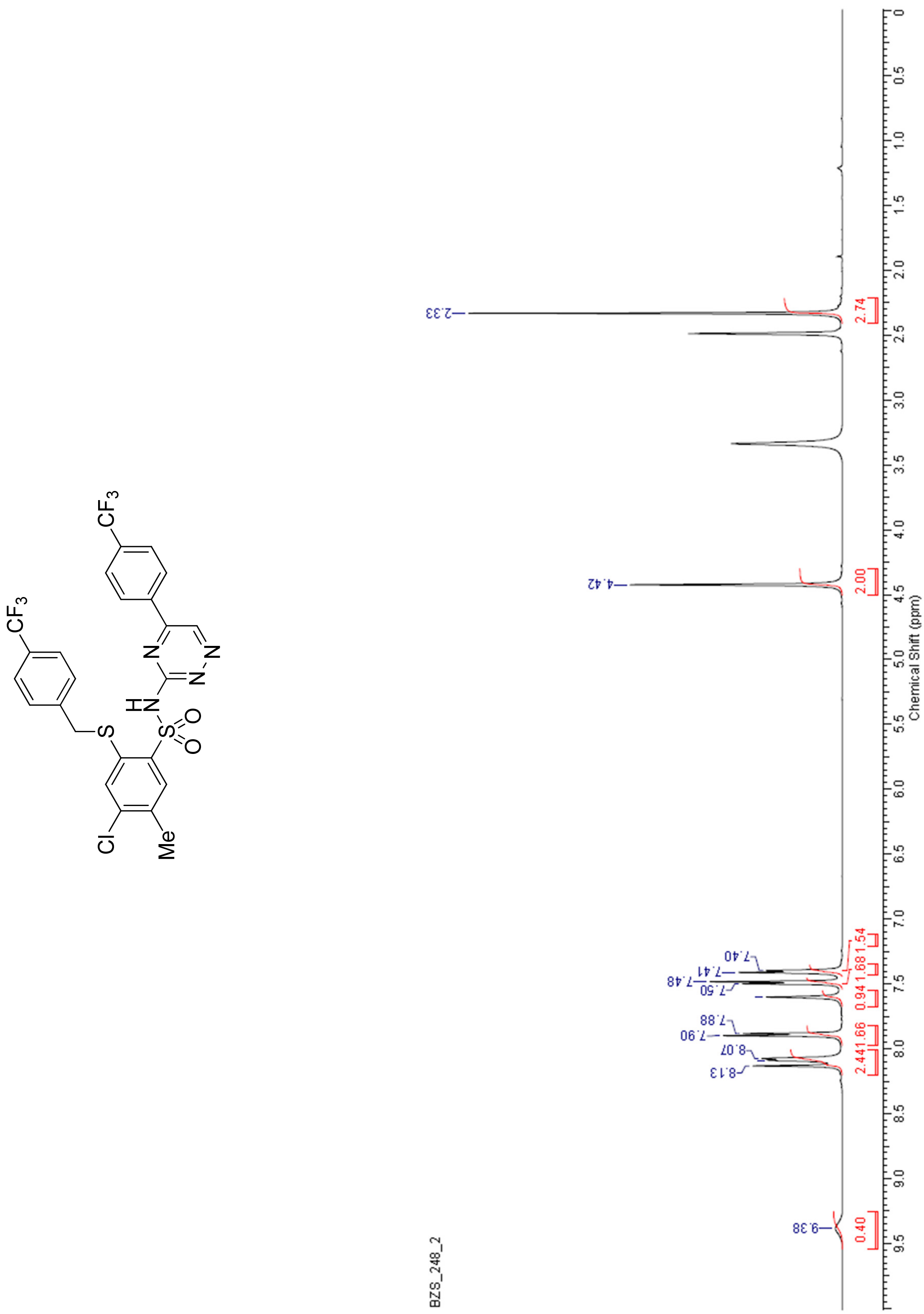

Spectrum S36. ^{1}H NMR of compd **21** (500 MHz. DMSO-*d*6).

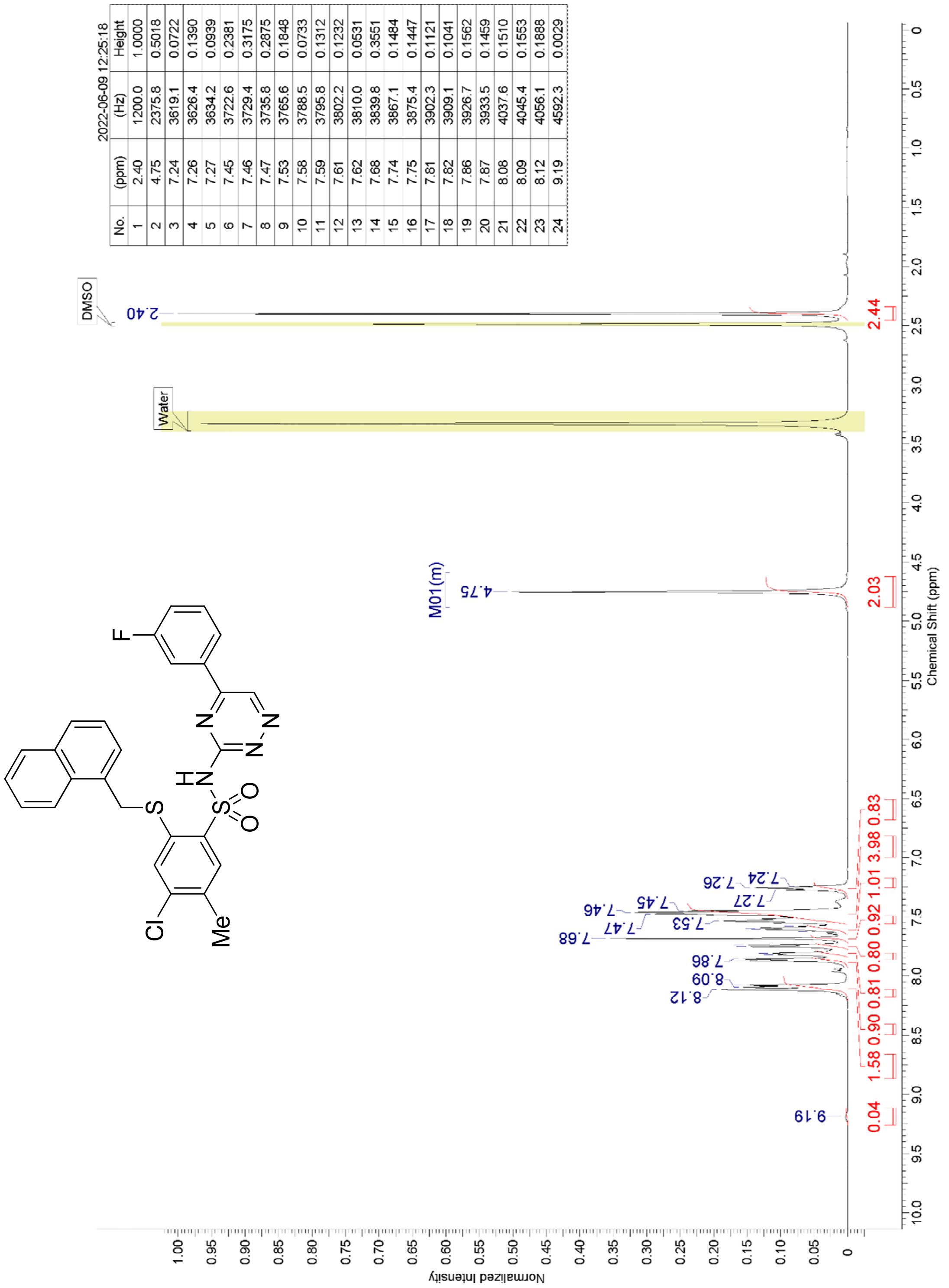

Spectrum S37. ^{1}H NMR of compd **22** (500 MHz. DMSO-d_6).

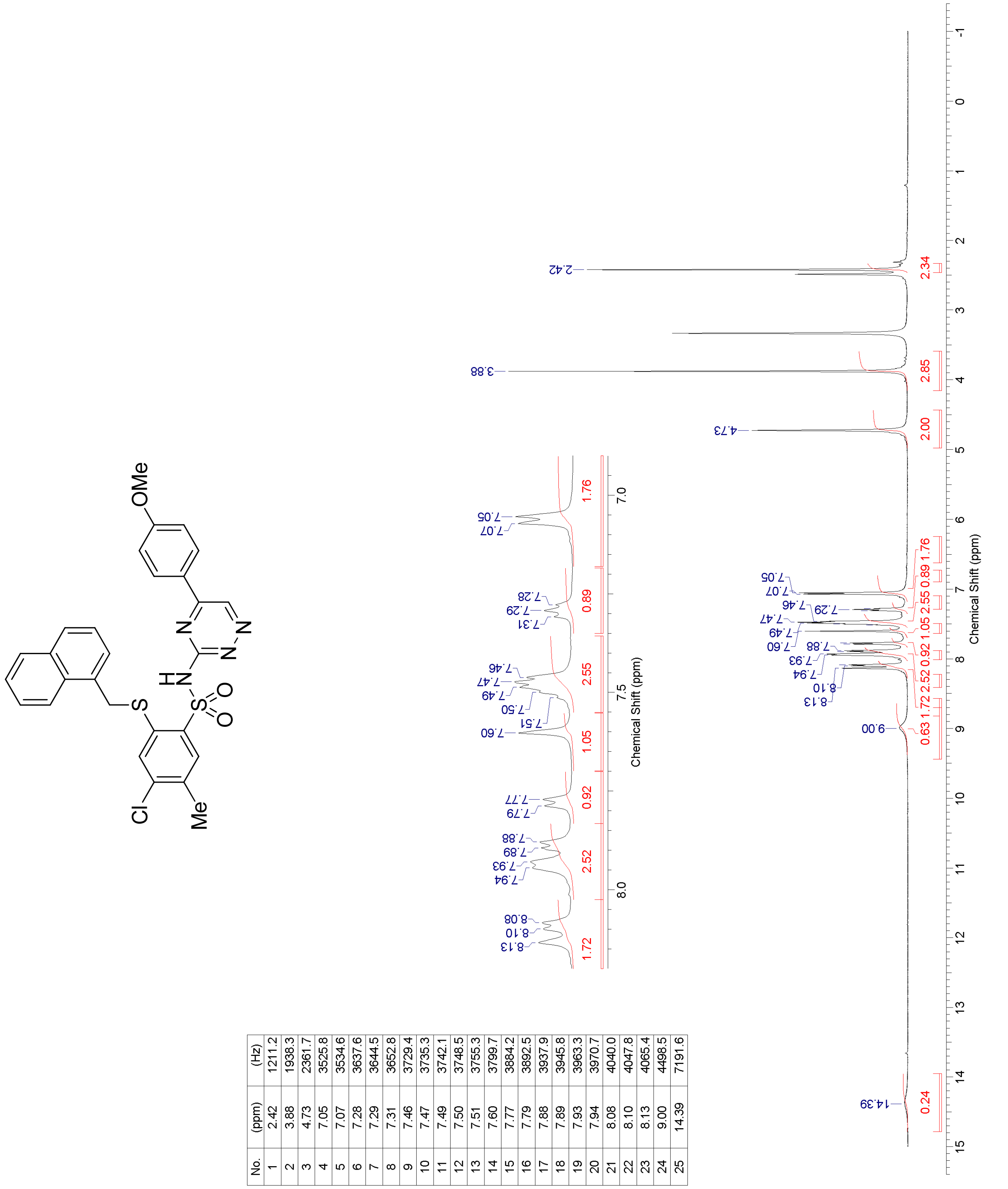

No.	(ppm)	(Hz)
1	2.42	1211.2
2	3.88	1938.3
3	4.73	2361.7
4	7.05	3525.8
5	7.07	3534.6
6	7.28	3637.6
7	7.29	3644.5
8	7.31	3652.8
9	7.46	3729.4
10	7.47	3735.3
11	7.49	3742.1
12	7.50	3748.5
13	7.51	3755.3
14	7.60	3799.7
15	7.77	3884.2
16	7.79	3892.5
17	7.88	3937.9
18	7.89	3945.8
19	7.93	3963.3
20	7.94	3970.7
21	8.08	4040.0
22	8.10	4047.8
23	8.13	4065.4
24	9.00	4498.5
25	14.39	7191.6

Spectrum S38. ^{1}H NMR of compd **23** (500 MHz. DMSO-*d*$_6$).

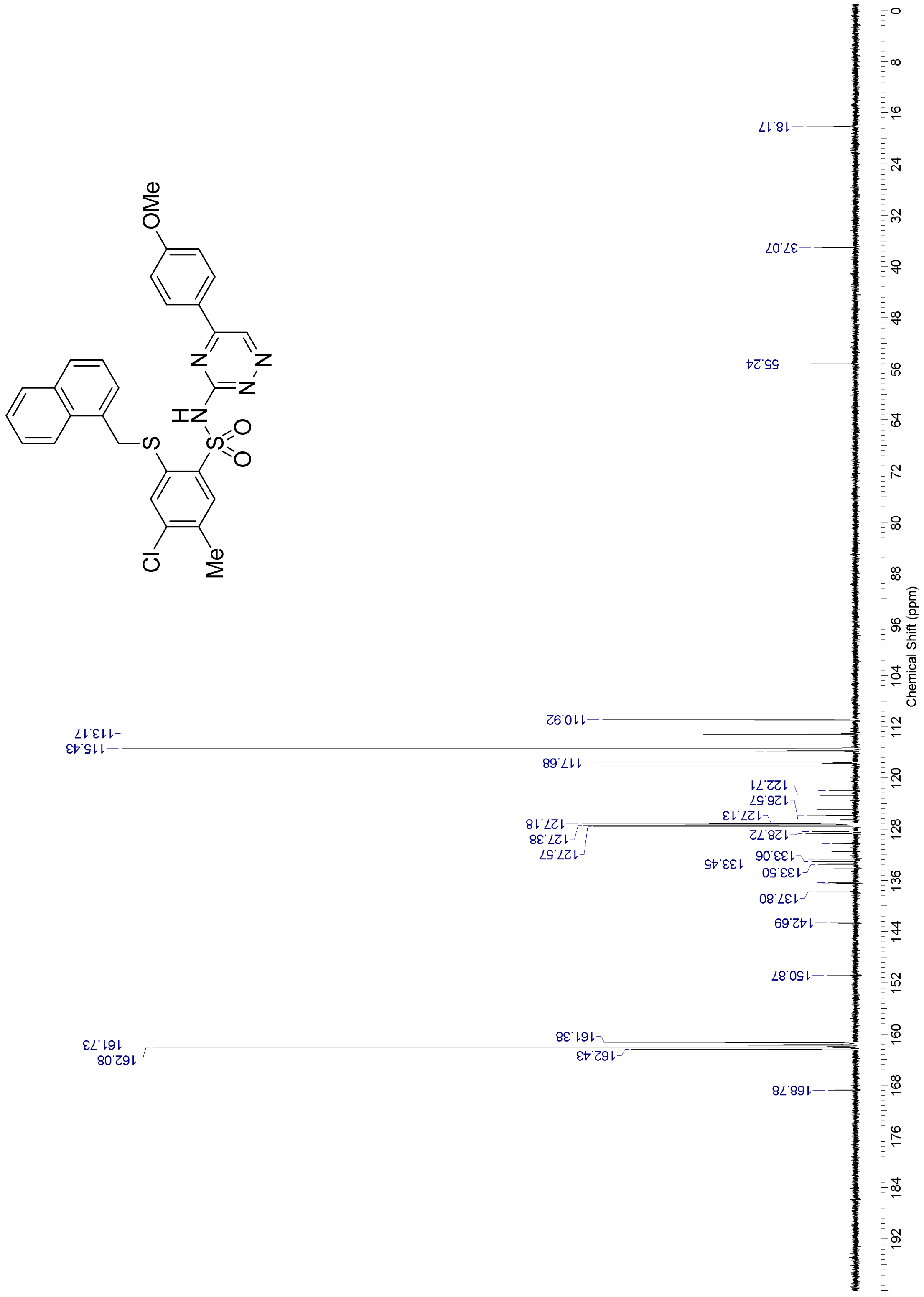

Spectrum S39. ^{13}C NMR of compd **23** (125 MHz. TFA).

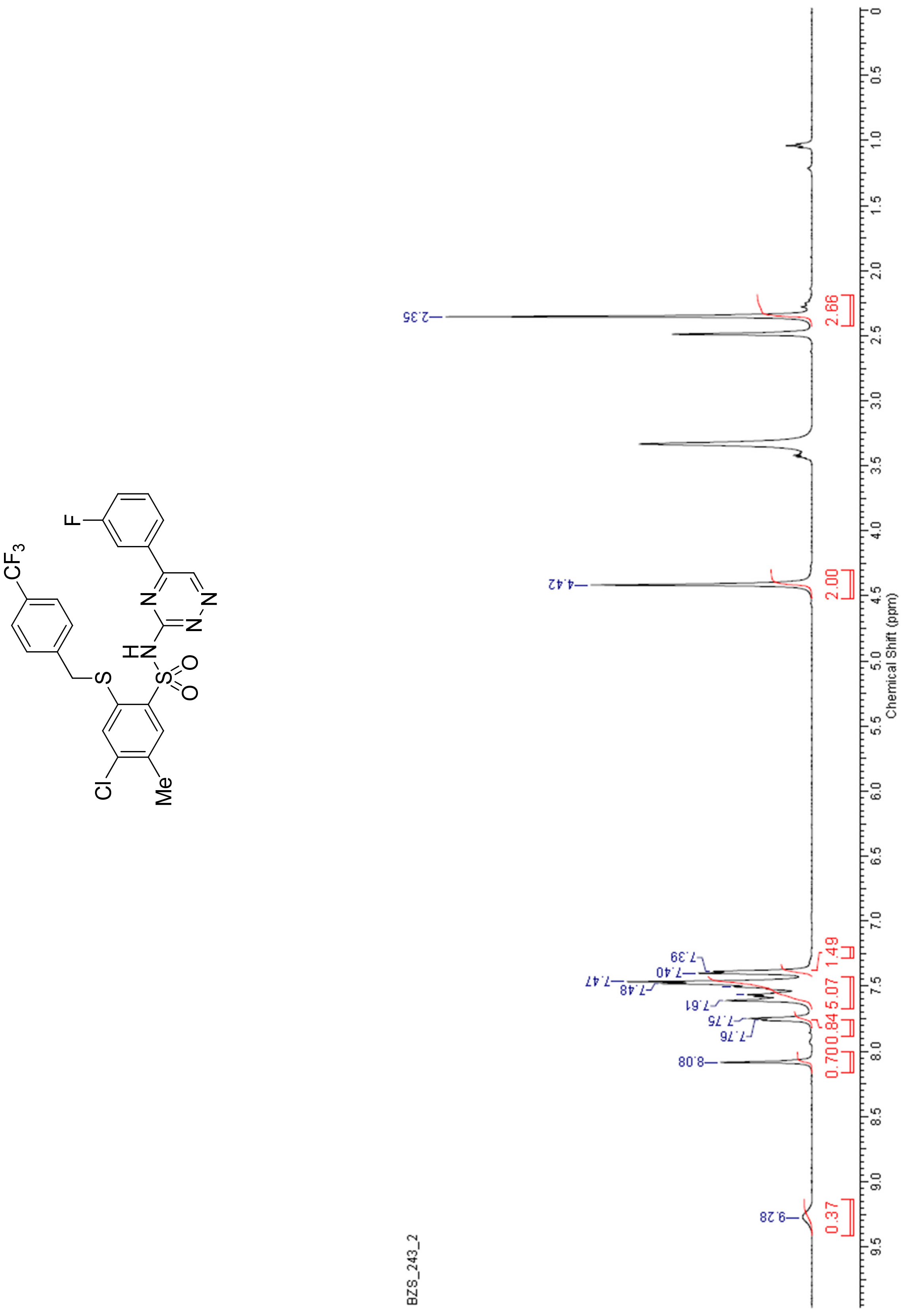

Spectrum S40. ^{1}H NMR of compd **24** (500 MHz. DMSO-d_6).

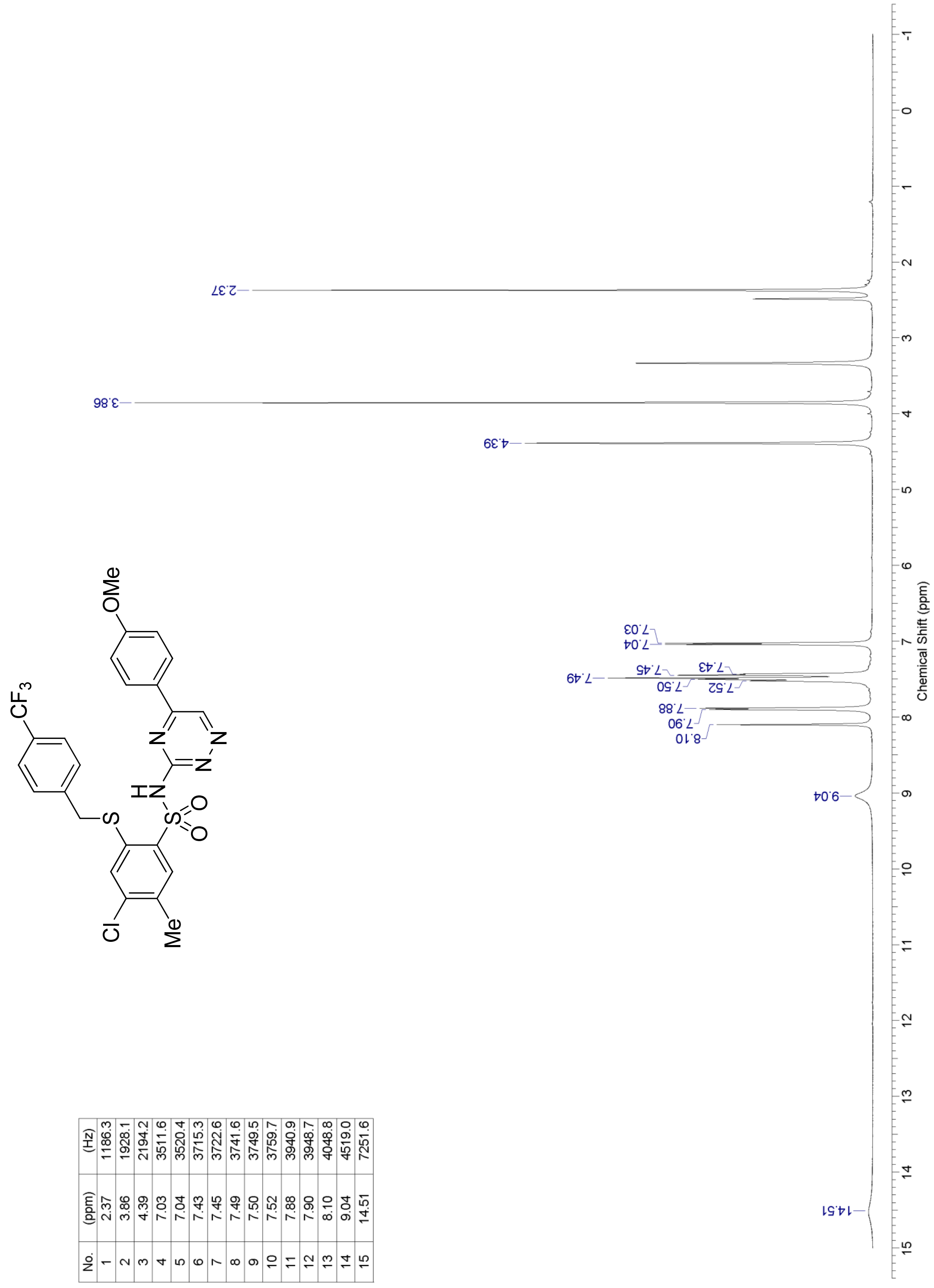

No.	(ppm)	(Hz)
1	2.37	1186.3
2	3.86	1928.1
3	4.39	2194.2
4	7.03	3511.6
5	7.04	3520.4
6	7.43	3715.3
7	7.45	3722.6
8	7.49	3741.6
9	7.50	3749.5
10	7.52	3759.7
11	7.88	3940.9
12	7.90	3948.7
13	8.10	4048.8
14	9.04	4519.0
15	14.51	7251.6

Spectrum S41. ^{1}H NMR of compd **25** (500 MHz. DMSO-d_6).

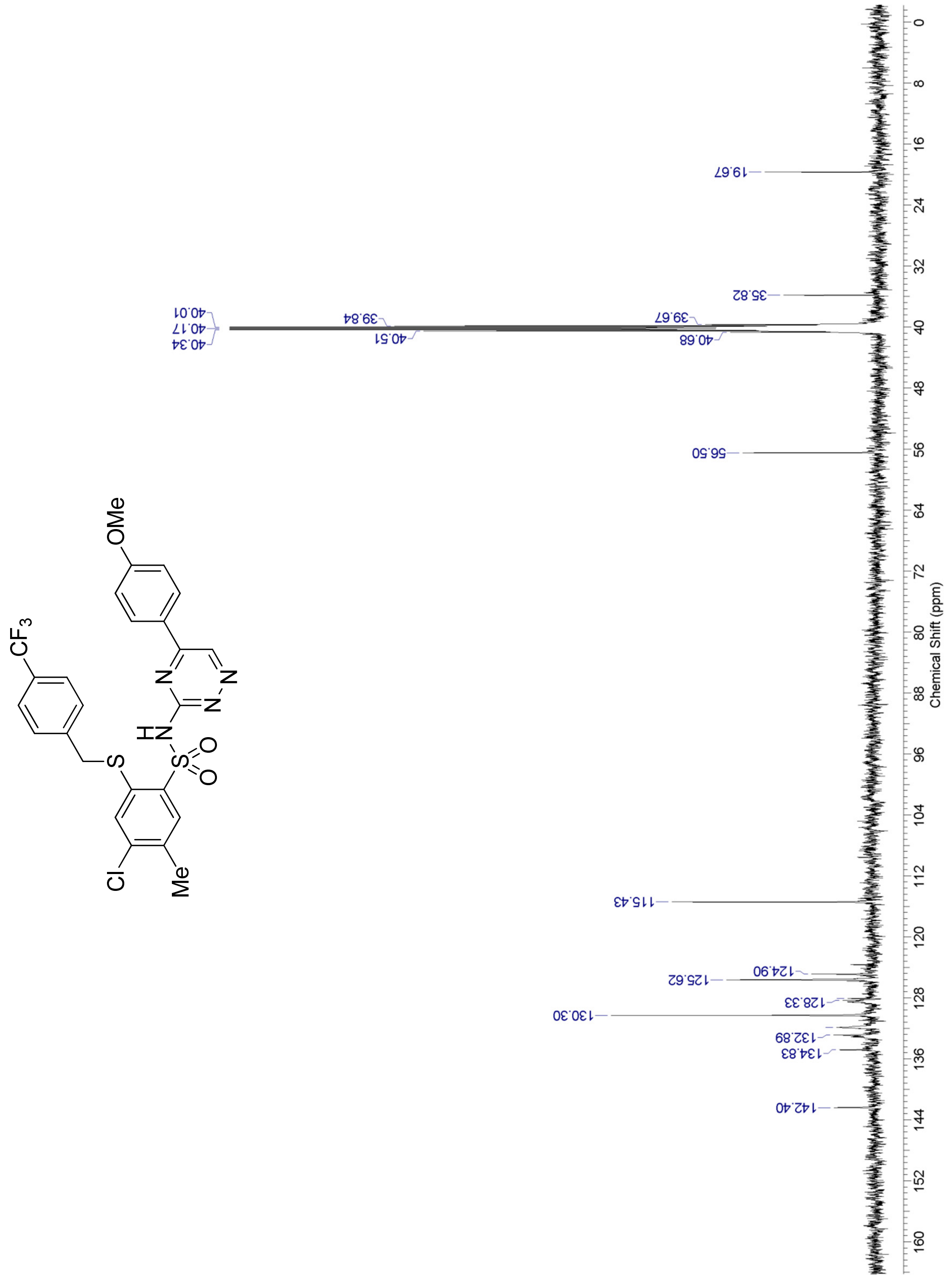

Spectrum S42. ^{13}C NMR of compd **25** (125 MHz. DMSO-*d*6).

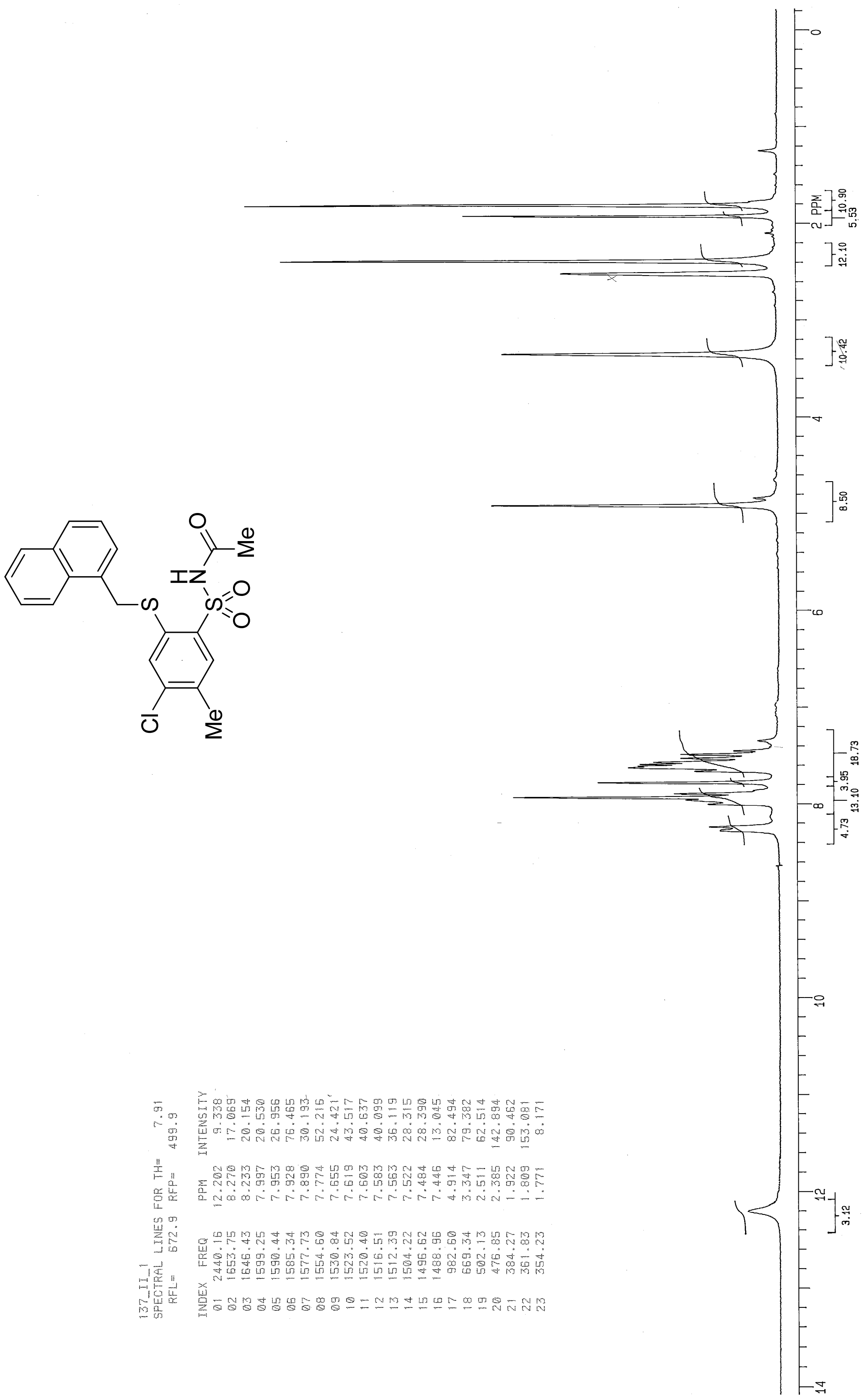

Spectrum S43. ^{1}H NMR of compd **26** (200 MHz. DMSO-d$_6$).

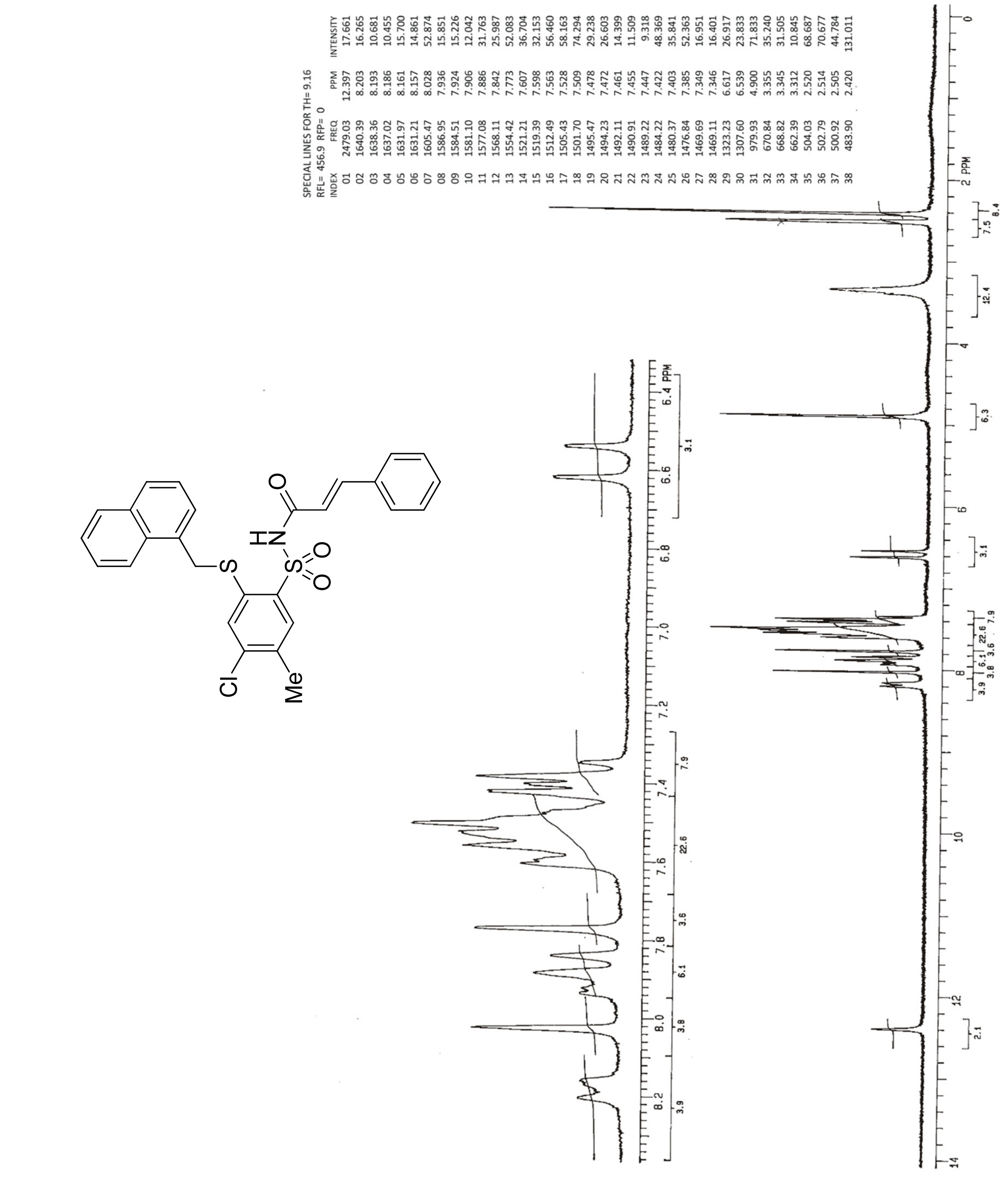

Spectrum S44. ^{1}H NMR of compd **27** (200 MHz. DMSO-d_6).

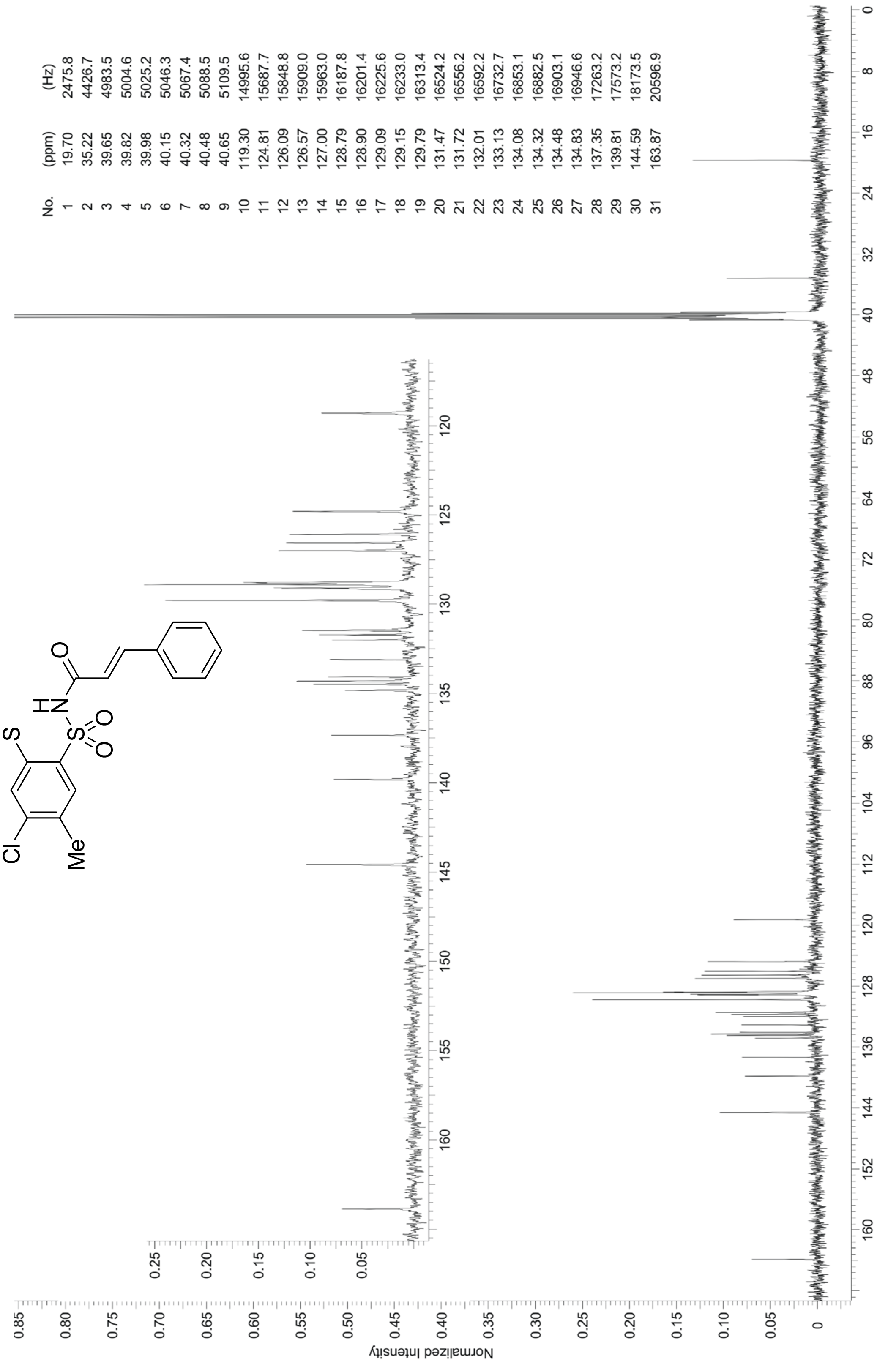

Spectrum S45. ^{13}C NMR of compd **27** (125 MHz. DMSO-d$_6$)

Method of synthesis and full structure analysis of compounds **1-27** have been published in:

*Compounds **1-7**;* B. Żołnowska. J. Sławiński. Z. Brzozowski. A. Kawiak. M. Belka. J. Zielińska. T. Bączek. J. Chojnacki. Synthesis. Molecular Structure. Anticancer Activity. and QSAR Study of N-(aryl/heteroaryl)-4-(1H-pyrrol-1-yl)Benzenesulfonamide Derivatives. Int. J. Mol. Sci. 19 (2018). https://doi.org/10.3390/ijms19051482.

Compounds ***8-12**;* B. Żołnowska. J. Sławiński. A. Pogorzelska. K. Szafrański. A. Kawiak. G. Stasiłojć. M. Belka. J. Zielińska. T. Bączek. Synthesis. QSAR studies. and metabolic stability of novel 2-alkylthio-4-chloro-N-(5-oxo-4.5-dihydro-1.2.4-triazin-3-yl)benzenesulfonamide derivatives as potential anticancer and apoptosis-inducing agents.. Chem. Biol. Drug Des. 90 (2017) 380–396. https://doi.org/10.1111/cbdd.12955.

Compounds ***13-25**;* B. Zołnowska. J. Sławiński. A. Pogorzelska. K. Szafrański. A. Kawiak. G. Stasiłojć. M. Belka. S. Ulenberg. T. Baczek. J. Chojnacki. Novel 5-substituted 2-(aylmethylthio)-4-chloro-N-(5-aryl-1.2.4-triazin-3-yl)benzenesulfonamides: Synthesis. molecular structure. anticancer activity. apoptosis-inducing activity and metabolic stability. Molecules. 21 (2016). https://doi.org/10.3390/molecules21060808.

Compounds ***26-27**;* B. Żołnowska. J. Sławiński. M. Belka. T. Bączek. A. Kawiak. J. Chojnacki. A. Pogorzelska. K. Szafrański. Synthesis. Molecular Structure. Metabolic Stability and QSAR Studies of a Novel Series of Anticancer N-Acylbenzenesulfonamides. Molecules. 20 (2015) 19101–19129. https://doi.org/10.3390/molecules201019101.
